# Association and risk prediction of 19 complex diseases with polygenic scores and socioeconomic status

**DOI:** 10.1038/s43856-026-01716-1

**Published:** 2026-06-11

**Authors:** Fiona A. Hagenbeek, Anne Richmond, Max Tamlander, Kira Detrois, Zhiyu Yang, Tuomo Hartonen, Daniel L. McCartney, FinnGen, Zhiyu Yang, Aarno Palotie, Mark Daly, Bridget Riley-Gills, Howard Jacob, Coralie Viollet, Slavé Petrovski, Alix Berton, Santha Ramakrishnan, Ellen Tsai, Zhihao Ding, Emily Holzinger, Robert Plenge, Joseph Maranville, Mark McCarthy, Rion Pendergrass, Jonathan Davitte, Chia-Yen Chen, Melis Atalar Aksit, Anna Vlahiotis, Katherine Klinger, Clement Chatelain, Jorg Blankenstein, Karol Estrada, Robert Graham, Dawn Waterworth, Chris O´Donnell, Nicole Renaud, Tomi P. Mäkelä, Jaakko Kaprio, Minna Ruddock, Lila Kallio, Antti Hakanen, Terhi Kilpi, Markus Perola, Jukka Partanen, Taneli Raivio, Eero Punkka, Teija Kekonen, Raisa Serpi, Kati Kristiansson, Sanna Siltanen, Veli-Matti Kosma, Arto Mannermaa, Jari Laukkanen, Tiina Jokela, Mervi Ahlroth, Johanna Mäkelä, Outi Tuovila, Jeffrey Waring, Fedik Rahimov, Ioanna Tachmazidou, Marc Jung, Hanati Tuoken, Shameek Biswas, Benjamin Sun, Neha Raghavan, Jae-Hoon Sul, Xinli Hu, Ma´en Obeidat, Jonathan Chung, Jonas Zierer, Mari Niemi, Johanna Schleutker, Tiina Wahlfors, Mikko Arvas, Olli Carpén, Reetta Hinttala, Johannes Kettunen, Katriina Aalto-Setälä, Mika Kähönen, Hanna Kujala, Triin Laisk, Natalia Pujol, Veikko Salomaa, Jaana Suvisaari, Satu Koskela, Jouni Lauronen, Kristiina Aittomäki, Pirkko Pussinen, Tuomo Meretoja, Heikki Joensuu, Peeter Karihtala, Emma Juuri, Aino Salminen, Tuula Salo, David Rice, Pekka Nieminen, Ulla Palotie, Fredrik Åberg, Daniel Gordin, Patrik Finne, Joni A. Turunen, Minna Raivio, Pentti Tienari, Martti Färkkilä, Jukka Koskela, Sampsa Pikkarainen, Kari Eklund, Paula Kauppi, Daniel Gordin, Juha Sinisalo, Marja-Riitta Taskinen, Tiinamaija Tuomi, Timo Hiltunen, Johanna Mattson, Eveliina Salminen, Terhi Ollila, Katariina Hannula-Jouppi, Oskari Heikinheimo, Ilkka Kalliala, Lauri Aaltonen, Erkki Isometsä, Antti Aarnisalo, Ilkka Immonen, Salla Ranta, Filip Scheperjans, Felix Vaura, Esa Pitkänen, Hannele Laivuori, Tuomo Kiiskinen, Katja Kivinen, Elisabeth Widen, Taru Tukiainen, Hanna Ollila, Elmo Saarentaus, Anne Kerola, Eero Vuoksimaa, Joni Lindbohm, Matthew Sampson, Michelle McNulty, Aoxing Liu, Joel Rämö, Austin Argentieri, Amanda Elliott, Elisa Rahikkala, Kirsi Sipilä, Valtteri Julkunen, Ville Leinonen, Sanna Toppila-Salmi, Mikko Hiltunen, Eino Solje, Hannu Kankaanranta, Antti Mäkitie, Iiris Hovatta, Niko Välimäki, Minttu Marttila, Anne Portaankorva, Eija Laakkonen, Heidi Silven, Eeva Sliz, Riikka Arffman, Susanna Savukoski, Riitta Kaarteenaho, Minna Karjalainen, Jaakko Tyrmi, Laura Kuusalo, Laura Pirilä, Tapio Hellman, Matti Vuori, Teemu Niiranen, Timo Blomster, Johanna Huhtakangas, Terttu Harju, Kaisa Tasanen, Laura Huilaja, Vuokko Anttonen, Marja Vääräsmäki, Outi Uimari, Laure Morin-Papunen, Maarit Niinimäki, Terhi Piltonen, Reetta Kälviäinen, Valtteri Julkunen, Hilkka Soininen, Mikko Kiviniemi, Oili Kaipiainen-Seppänen, Margit Pelkonen, Päivi Auvinen, Maria Siponen, Liisa Suominen, Päivi Mäntylä, Kai Kaarniranta, Jukka Peltola, Airi Jussila, Katri Kaukinen, Pia Isomäki, Jussi Hernesniemi, Annika Auranen, Hannu Uusitalo, Teea Salmi, Venla Kurra, Laura Kotaniemi-Talonen, Argyro Bizaki-Vallaskangas, Juha Rinne, Roosa Kallionpää, Markku Voutilainen, Antti Palomäki, Laura Pirilä, Riitta Lahesmaa, Kaj Metsärinne, Jenni Aittokallio, Klaus Elenius, Sirkku Peltonen, Leena Koulu, Ulvi Gursoy, Varpu Jokimaa, Tytti Willberg, Adam Ziemann, Nizar Smaoui, Anne Lehtonen, Apinya Lertratanakul, Relja Popovic, Mengzhen Liu, Anneke Den Hollander, Jan Freudenberg, Britney Milkovich, Andrew Blumenfeld, Tushar Kumar, Dirk Paul, Bram Prins, Eleanor Wheeler, Kousik Kundu, Santosh Atanur, Andrew Lowe, Thomas Spargo, Oliver Burren, Margarete Fabre, Fabio Baschiera, Hans van Leeuwen, Himanshu Manchanda, Karl Heilbron, Martin Rao, Nicole Schmidt, Samu Kurki, Johanna Mielke, Juho Immonen, Thomas Battram, Tobias Hogrebe, Susan Eaton, Ketian Yu, Stephanie Loomis, Coro Paisan-Ruiz, Elke Markert, Frank Li, Yao Hu, Christoph Ogris, Eric Simon, Julio Cesar Bolivar Lopez, Monika Frysz, Marla Hochfeld, Cara Carty, Michael Turchin, Neelakshi Jog, Corneliu Bodea, Janie Shelton, Chen Li, Kritika Singh, Peng Jiang, Stephanie Loomis, Elena Sanchez, Lilith Moss, Zijie Zhao, Anna Podgornaia, Natalie Bowers, Edmond Teng, Tim Lu, Hubert Chen, Jennifer Schutzman, Erich Strauss, Hao Chen, David Choy, Brian Yaspan, Cameron Adams, Michael Rothenberg, Sergio Dellepiane, Anubha Mahajan, Michael Holmes, Diana Chang, Tushar Bhangale, Fanli Xu, Laura Addis, John Eicher, Linda McCarthy, Jorge Esparza Gordillo, Joanna Betts, Rajashree Mishra, Audrey Chu, Diptee Kulkarni, Janet Kumar, Charli Harlow, Lea Sarow-Blat, Diana L. Cousminer, Jagtar Nijjar, Jessica Chao, Michal Magid, Shashank Jariwala, Chris Floyd, Dan Swerdlow, Erding Hu, Prerak Desai, Stephen Haddad, Damien Croteau-Chonka, Billy Fahy, Paola Bronson, Kirsi Auro, David Pulford, Sauli Vuoti, Dermot Reilly, Karen He, Ekaterina Khramtsova, Amy Hart, Meijian Guan, Alessandro Porello, P. Dunnmon, Sara Gale, Brice Keyes, John Kwon, Jonathan Sherlock, Matt Loza, Chris Whelan, W. Galpern, Yanfei Zhang, Mona Selej, Abolfazl Doostparast Torshizi, Qingqin S. Li, Sahar Mozzafari, Christopher Deboever, Jason Miller, Fabiana Farias, Andrey Loboda, Jorge Del-Aguila, Elisabeth Vollmann, Jozsef Karman, Julie Fiore, Rajesh Kamath, Andrei Popescu, Delphine Fagegaltier, Travis Barr, Aristide Merola, Oliver Freeman, Simonne Longerich, Enrico Ferrero, Nikos Patsopoulos, Nancy Finkel, Sabina Pfister, Shola Richards, Katherine Mccauley, Xiaobo Xia, Mike Mendelson, Majd Mouded, Debby Ngo, Kirsi Kalpala, Melissa Miller, Nan Bing, Jaakko Parkkinen, Heli Lehtonen, Stefan McDonough, Ying Wu, Erin Macdonald-Dunlop, Jessica Chung, Michael McLean, Joshua Chiou, Hye In Kim, Sivakumar Pitchumani, Sumedha Jassal, Madhurima Saxena, Catherine O’Riordan, Samuel Lessard, Suzanne Jacobs, Hamid Mattoo, David Habiel, Guanling Huan, Anu Jalanko, Risto Kajanne, Mervi Aavikko, Helen Cooper, Denise Öller, Tarja Laitinen, Rodos Rodosthenous, Sofia Kuitunen, Mitja Kurki, Juha Karjalainen, Pietro Della Briotta Parolo, Arto Lehisto, Juha Mehtonen, Reza Jabal, Mutaamba Maasha, Sanni Ruotsalainen, Samuel Jones, Raymond Walters, Paavo Häppölä, Topi Paavilainen, L. Elisa Lahtela, Johanna Paltta, Juulia Partanen, Olli K. Pietiläinen, Veera Timonen, Mari Kaunisto, Elina Kilpeläinen, Tianduanyi Wang, Timo P. Sipilä, Oluwaseun Alexander Dada, Awaisa Ghazal, Rigbe Weldatsadik, Jaska Uimonen, Kati Donner, Anu Loukola, Päivi Laiho, Susanna Lemmelä, Teemu Paajanen, Arto Pietilä, Aki Havulinna, Auli Toivola, Kristina Zguro, Mary Pat Reeve, Shanmukha Sampath Padmanabhuni, Harri Siirtola, Javier Gracia-Tabuenca, Marika Kaakinen, Shuang Luo, Vincent Llorens, Dawit Yohannes, Iina Laak, Pauli Wihuri, Tom Southerington, Meri Lähteenmäki, Nina Mars, Samuli Ripatti, Riccardo E. Marioni, Pekka Martikainen, Nina Mars, Andrea Ganna, Samuli Ripatti

**Affiliations:** 1 Institute for Molecular Medicine Finland (FIMM), Helsinki Institute of Life Science (HiLIFE), University of Helsinki, Helsinki, Finland; 2 Department of Biological Psychology, Vrije Universiteit Amsterdam, Amsterdam, The Netherlands; 3 Amsterdam Public Health (APH) Research Institute, Amsterdam, The Netherlands; 4 Institute of Genetics and Cancer, University of Edinburgh, Edinburgh, UK; 5 Helsinki Institute for Demography and Population Health, Faculty of Social Sciences, University of Helsinki, Helsinki, Finland; 6 Max Planck – University of Helsinki Center for Social Inequalities in Population Health, Helsinki, Finland; 7 Stanley Center for Psychiatric Research, Broad Institute of MIT and Harvard, Cambridge, MA USA; 8 Massachusetts General Hospital and Broad Institute of MIT and Harvard, Cambridge, MA USA; 9 Department of Public Health, University of Helsinki, Helsinki, Finland; 10 Abbvie, Chicago, IL USA; 11 AstraZeneca, Cambridge, UK; 12 Bayer AG, Leverkusen, Germany; 13 Biogen, Cambridge, MA USA; 14 Boehringer Ingelheim, Ingelheim am Rhein, Rhein, Germany; 15 Bristol Myers Squibb, New York, NY USA; 16 Genentech, San Francisco, CA USA; 17 GlaxoSmithKline, Collegeville, PA USA; 18 Merck, Kenilworth, NJ USA; 19 Pfizer, New York, NY USA; 20 Translational Sciences, Sanofi R&D, Framingham, MA USA; 21 Maze Therapeutics, San Francisco, CA USA; 22 Johnson & Johnson Innovative Medicine, Spring House, PA USA; 23 Novartis Institutes for BioMedical Research, Cambridge, MA USA; 24 Helsinki Institute of Life Science (HiLIFE), University of Helsinki, Helsinki, Finland; 25 Arctic biobank, University of Oulu, Oulu, Finland; 26 Auria Biobank, University of Turku, Turku, Finland; 27 University of Turku, Turku, Finland; 28 Wellbeing Services County of Southwest Finland, Turku, Finland; 29 THL Biobank, Finnish Institute for Health and Welfare (THL), Helsinki, Finland; 30 Finnish Institute for Health and Welfare (THL), Helsinki, Finland; 31 Finnish Red Cross Blood Service, Finnish Hematology Registry and Clinical Biobank, Helsinki, Finland; 32 Helsinki Biobank, Helsinki University and Hospital District of Helsinki and Uusimaa, Helsinki, Finland; 33 University of Helsinki, Helsinki, Finland; 34 Hospital District of Helsinki and Uusimaa, Helsinki, Finland; 35 Northern Finland Biobank Borealis, University of Oulu, Oulu, Finland; 36 University of Oulu, Oulu, Finland; 37 Wellbeing services county of North Ostrobothnia, Oulu, Finland; 38 Finnish Clinical Biobank Tampere, University of Tampere, Tampere, Finland; 39 University of Tampere, Tampere, Finland; 40 Wellbeing Services County of Pirkanmaa, Tampere, Finland; 41 Biobank of Eastern Finland, University of Eastern Finland, Kuopio, Finland; 42 University of Eastern Finland, Kuopio, Finland; 43 Wellbeing services county of North Savo, Kuopio, Finland; 44 Central Finland Biobank, University of Jyväskylä, Jyväskylä, Finland; 45 University of Jyväskylä, Jyväskylä, Finland; 46 Wellbeing Services County of Central Finland, Jyväskylä, Finland; 47 Finnish Biobank Cooperative – FINBB, Helsinki, Finland; 48 Business Finland, Helsinki, Finland; 49 Faculty of Medicine and Health Technology, Tampere University, Tampere, Finland; 50 Estonian biobank, Tartu, Estonia; 51 Helsinki University Hospital, Helsinki, Finland; 52 Folkhälsan Research Center, Helsinki, Finland; 53 Broad Institute of MIT and Harvard, Cambridge, MA USA; 54 Harvard Medical School, Cambridge, MA USA; 55 Northern Ostrobothnia Hospital District, Oulu, Finland; 56 Oulu University Hospital, Oulu, Finland; 57 Kuopio University Hospital, Kuopio, Finland; 58 University of Gothenburg, Gothenburg, Sweden; 59 Seinäjoki Central Hospital, Seinäjoki, Finland; 60 University of Lodz, Lodz, Poland; 61 GlaxoSmithKline, Brentford, UK; 62 GlaxoSmithKline, Espoo, Finland; 63 GlaxoSmithKline, Stevenage, UK; 64 Janssen-Cilag Oy, Espoo, Finland; 65 Johnson & Johnson Innovative Medicine, Boston, MA USA; 66 Johnson & Johnson Innovative Medicine, Titusville, NJ USA; 67 Novartis, Basel, Switzerland

**Keywords:** Diseases, Personalized medicine

## Abstract

**Background:**

Differences in disease risk are linked to inherited genetic variation and social circumstances, but how these factors jointly relate to multiple diseases is not fully understood. This study investigates the independent and combined associations of genetic predisposition and socioeconomic status with common diseases.

**Methods:**

We analyzed data from 743,194 adults from three biobank studies in Finland and the United Kingdom, including European and non-European ancestry individuals. Genetic predisposition was measured using polygenic scores for 19 common diseases. Socioeconomic status was assessed through education and occupation. We evaluated associations of genetic and socioeconomic factors with disease risk and whether genetic associations differed across education levels. Models including both genetic and educational factors were compared with models including only one factor and models including their interaction.

**Results:**

Here, we show that both genetic predisposition and socioeconomic status are associated with disease risk across most conditions. For seven diseases, genetic associations are stronger in highly educated groups, while occupation shows smaller differences. Patterns are generally consistent across ancestry groups, though education associations are less stable in non-European individuals. While both education and polygenic scores independently improved prediction, combining both provided additional modest improvement to prediction for 16 and 12 out of 19 diseases in FinnGen and the UK Biobank, respectively.

**Conclusions:**

Genetic variation and social circumstances are linked to differences in disease risk both independently, and in combination with one another. Considering both factors modestly improves identification of higher-risk groups and highlights the value of accounting for social context alongside inherited variation in population-level assessments.

## Plain Language Summary

This study explored how genetic predisposition and socioeconomic status relate to the risk of common diseases. Genes contribute to differences in disease susceptibility, while social factors, such as education and occupation, also vary across populations. Using data from over 700,000 adults in Finland and the United Kingdom, we combined polygenic scores (measures of inherited genetic variation) with information on education and occupation to examine their independent and joint associations with 19 diseases. We found that both genetics and education were linked to disease risk, and for seven conditions, the genetic associations were stronger in individuals with high education. Combining genetic and education information modestly improved disease prediction, highlighting their complementary roles in identifying high-risk groups.

## Abstract (web summary)

Hagenbeek et al. examine how genetic variation and socioeconomic status relate to the risk of 19 common diseases in over 700,000 adults from Finland and the UK. They show both genetics and education are linked to disease risk, with some genetic associations varying across education levels, and combining both modestly improves risk prediction.

## Introduction

Socioeconomic inequalities are linked to increased disease burden for many complex diseases^1–3^. Most diseases have a higher incidence among individuals with low socioeconomic status (SES), such as cardiometabolic and mental health disorders^1,2^. For example, individuals without formal schooling have a 30% higher overall disease burden than individuals with higher educational attainment^3^. In contrast, cancers, particularly breast cancer and skin melanoma, are more prevalent in individuals with high SES^2,4,5^, especially in older age groups^1^.

In addition to socioeconomic differences, genetic factors affect complex disease susceptibility, and genome-wide association studies (GWAS) have successfully identified thousands of common variant associations for complex traits and diseases^6^. The availability of large GWAS results has led to the development of polygenic scores (PGS), which sum the effects of many common genetic variants of small effect sizes into a single score predictive of inherited disease risk or complex trait outcomes^7,8^. PGSs significantly predict diseases^9,10^, and have been shown to provide additional information for disease risk when considered alongside clinical risk factors^11,12^ or family history^13^. Also, socioeconomic measures have substantial environmental influences but also genetic influences, particularly for educational attainment^14^. PGSs for educational attainment predict common diseases such as major depression, type 2 diabetes (T2D), and heart disease, and including the PGS for educational attainment, the disease-specific PGS, and their interaction substantially increases disease prediction^14^. These results show that genetic factors are associated with both socioeconomic factors and complex disease risks.

While PGSs capture inherited susceptibility, genetic risk does not imply determinism^15^. Evidence from coronary artery disease and T2D shows that lifestyle interventions can meaningfully reduce risk in genetically high-risk individuals^16,17^. These findings underscore the value of integrating genetic and environmental risk information to inform preventive strategies. In the current study, we aim to explore the associations between PGSs and SES, as measured by educational attainment and occupation, on complex disease risk for 19 high-burden diseases^18^ with known socioeconomic and genetic differences in risk across three studies in Finland and the United Kingdom (*N* = 729,928). We set out to test the following hypotheses of public health importance: 1) both PGSs and SES are independently associated with 19 common diseases, 2) the strength of the association between PGSs and diseases differs across levels of SES, and 3) models that include both PGSs and SES provide better predictive ability for complex diseases than models including only one of these factors.

Consistent with our hypotheses, we find that both PGSs and SES were independently associated with disease risk across most of the 19 diseases studied. For several diseases, including coronary heart disease (CHD), type 2 diabetes, atrial fibrillation (AF), osteoarthritis, asthma, and any cancer, the association of the PGSs with disease was stronger in the highly educated group, while occupation showed less pronounced modification. Integrating PGSs with educational attainment modestly improves disease prediction compared to models including only one factor, highlighting the complementary contribution of genetic and socioeconomic information. These results suggest that genetic susceptibility is context-dependent and that combining genetic and social measures may enhance population-level risk stratification, providing insights relevant for preventive strategies.

## Methods

### Participating studies

We analyzed data from 729,928 European ancestry participants from three biobank studies across Finland and the United Kingdom (FinnGen^19^, UK Biobank^20^, and Generation Scotland (GS)^21^), as well as from 13,266 non-European ancestry participants from the UK Biobank. Participants were included if they were aged 35 to 80 at the time of study entry, to ensure most individuals had completed their formal education.

### Selection and harmonization of complex diseases

We examined 19 diseases, selected based on their high burden in high-income countries according to the Global Burden of Disease (GBD)^18^ and the availability of well-powered GWAS. Eighteen of these diseases were previously analyzed by the INTERVENE consortium^22^. We additionally included alcohol use disorder (AUD), given its known socioeconomic disparities in outcomes and high public health burden^23^, particularly in Finland^18^. Moreover, large-scale GWAS summary statistics for alcohol consumption are available^24^, allowing comparable PGS estimation.

Disease case definitions followed Jermy et al. (2024)^22^: participants were classified as cases based on the presence of harmonized ICD-9 or ICD-10 codes, as curated by clinical experts in FinnGen^19^. Controls were defined as individuals without those codes. Diagnoses were extracted from the health registries or electronic health records and were thus not limited to only information from after study enrollment (for information on the health registries, see the section below).

### Registry data

#### FinnGen (Data Freeze 11)

The construction of phenotype data in FinnGen involves the aggregation of information from nationwide electronic health registers. This encompasses nearly all aspects of a patient’s interactions with the health service, including hospitalizations, medications, procedures, and deaths. The project’s 18 registers are presented below, organized by their follow-up times.

- Finnish Cancer Registry—From 1953
- Register of Congenital Malformations—From 1963
- Reimbursement—From 1964
- Population Register—From 1964
- Finnish Registry for Kidney Diseases—From 1964
- Causes of Death—From 1969
- Care Register for Health Care Inpatient Visits, HILMO—From 1969
- Socio-economic data—From 1970
- The Finnish Registry of Visual Impairment—From 1983
- Medical Birth Register—From 1987
- Finnish National Infectious Disease Register—From 1989
- Cervical Cancer Screening—From 1991
- Breast Cancer Screening—From 1992
- Drug Purchases—From 1995
- The Care Register for Social Welfare—From 1995
- Care Register for Health Care, specialist outpatient visits, HILMO —From 1998
- Register of Primary Health Care Visits, Avohilmo—From 2011
- The Finnish Vaccination Register—From 2011

Note: Although primary health care visits are covered in FinnGen, they are typically not included in the endpoints. Therefore, we only analyze secondary care data for our disease outcomes.

#### UK Biobank

The relevant columns used to define the phenotypes were:

- Cause of Death Primary (Column ID: 40001)
- Cause of Death Secondary (Column ID: 40002)
- Summary ICD10 Diagnoses (Column ID: 41270)
- Summary ICD10 Diagnoses Date (Column ID: 41280)
- Summary ICD9 Diagnoses (Column ID: 41271)
- Summary ICD9 Diagnoses Date (Column ID: 41281)

Such data are taken from the hospital episode statistics, which relate to hospital inpatient data. For more information, please see https://biobank.ndph.ox.ac.uk/showcase/showcase/docs/HospitalEpisodeStatistics.pdf. Registry coverage depends on the country, with follow-up beginning in 1997, 1998, and 1981 for England, Wales, and Scotland, respectively. The end of follow-up was stated as 31st January 2021.

#### GS

Disease outcomes were ascertained through linkage to primary (GP) and secondary (hospital) healthcare records. Individuals were subsetted to those registered at a GP who consented to the sharing of primary records. GP records consisted of Read2 codes, which were mapped to ICD-10. Hospital data were obtained from Scottish Morbidity Records (SMR), where disease outcomes were coded using ICD-9 (pre March 1997) or ICD-10 (post March 1997). The start of follow-up was considered to be the latest date between the date of birth or March 1980, the date of GP linkage. End of follow-up was considered to be October 2020 (the date to which GP data is available), date of death, or date of disease onset if after October 2020 (hospital data is available until March 2022).

### Harmonization educational attainment

To harmonize education across countries, we converted national education levels to the 1997 International Standard Classification of Education (ISCED)^25^. We then dichotomized education into low (ISCED ≤ 4) and high (ISCED ≥ 5) categories (see Supplementary Data 1). This cutoff was based on the distribution of education in FinnGen, chosen to ensure sufficient sample sizes and adequate case numbers within each group for all outcomes.

### Selection and harmonization occupation

#### FinnGen (Data Freeze 11)

Information about occupation in FinnGen was retrieved from Statistics Finland using the classification of socioeconomic groups, which in turn is based on the classification of occupations (for details see: https://stat.fi/en/luokitukset/sosioekon_asema/sosioekon_asema_1_19890101). Occupation was available for a maximum of 25 different years (1970-2020): 1970, 1975, 1980, 1990, 1993, 1995, 2000, and annually from 2004 onwards. Occupation was captured into 9 broad categories: 1) self-employed; 2) upper-level employees with administrative, managerial, professional, and related occupations; 3) lower-level employees with administrative and clerical occupations; 4) manual workers; 5) students; 6) pensioners; 7) others (including unemployed); 8) unknown occupation; and 9) missing occupation. For each individual, we selected the occupation closest to the year of the event for cases or the end of follow-up for controls. In case of ties, i.e., when the event/end-of-follow-up fell in between measurements, we selected the earlier occupation information. Whenever the occupation class closest to event/end-of-follow-up was ‘students’, ‘pensioners’, ‘other’, ‘unknown’, or ‘missing’, we iteratively extracted the occupation class at a previous census until an individual could be assigned to ‘self-employed’, ‘upper-level employees with administrative, managerial, professional and related occupations’, ‘lower-level employees with administrative and clerical occupations’, or ‘manuals workers’. Whenever this reassignment to an earlier census date was not possible, the individual was excluded from the analyses. To ensure easier comparison between the educational attainment and occupation analyses, we combined the ‘manual workers’ and ‘lower-level employees with administrative and clerical occupations’ into a single ‘lower-level’ occupation class and compared this group to individuals with ‘upper-level employees with administrative, managerial, professional and related occupations’, which we refer to as ‘upper-level’ occupation in the manuscript.

#### UK biobank

Information about occupation in the UK Biobank was retrieved from ‘Job code at visit’ (field 20277). Job codes were captured into 9 broad categories: 1) managers and senior officials; 2) professional occupations; 3) associate professional and technical occupations; 4) administrative and secretarial occupations; 5) skilled trades occupations; 6) personal service occupations; 7) sales and customer service occupations; 8) process, plant, and machine operatives; and 9) elementary occupations. To harmonize occupation information with FinnGen, we combined categories 4–9 into a single ‘lower-level’ and categories 1–3 into a single ‘upper-level’ occupation category.

#### GS

In the GS occupation was assessed by classifying job types. Job types were classified into 26 broad categories: 1) architecture and engineering; 2) arts, design, entertainment, sports, and media; 3) building and grounds cleaning and maintenance; 4) business and financial operations; 5) community and social services; 6) computer and mathematical; 7) construction and extraction; 8) education, training, and library; 9) farming, fishing, and forestry; 10) food preparation and serving related; 11) healthcare practitioner and technical; 12) healthcare support; 13) installation, maintenance, and repair; 14) legal; 15) life, physical, and social science; 16) management; 17) military specific; 18) office and administrative support; 19) personal care and service; 20) production; 21) protective service; 22) retail, sales and marketing; 23) transportation and material moving; 24) never worked; 25) other; and 26) not applicable. Individuals who had never worked, or had another occupation, or for whom the job type was not applicable were excluded from the analyses. For all other individuals, the job type categories 3, 7, 9–13, 18, 20, and 23 were combined into ‘lower-level’ and categories 1–2, 4–6, 8, 14–17, 19, and 21–22 into ‘upper-level’ occupation to harmonize with FinnGen and the UK Biobank.

### Genotyping

#### FinnGen (Data Freeze 11)

##### Genotyping and quality control (QC)

In FinnGen, there are prospectively recruited samples, as well as legacy cohorts with pre-existing genotype data^19^. Genotyping of prospective samples was done with the ThermoFisher Axiom custom array (*N* = 371,914), which covers 655,973 variants. Genotype calling was conducted with the Array Power Tools software (https://www.thermofisher.com/us/en/home/life-science/microarray-analysis/microarray-analysis-partners-programs/affymetrix-developers-network/affymetrix-power-tools.html). Various Illumina arrays were used to genotype legacy cohorts (*N* = 81,819, for details see ref. ^19^), and either GenCall or zCall algorithms were employed for genotype calling^26^.

The same QC metrics were applied to both prospective and legacy cohorts. QC was performed per genotyping batch, information on the number of samples and variants removed per batch is provided in Supplementary Data 2.

Samples were removed if:

- The proportion of identity-by-descent (PLINK’s v2^27^ Pi hat) was >0.9, and the samples were not monozygotic or replicates
- The reported sex did not match the genetically determined sex (*F* value ≤ 0.3 for female participants and ≥0.8 for male participants).
- Missingness was ≥5%
- Heterozygosity was ±4 standard deviations (SDs) from the population average
- Pi hat was >0.1 with 14 or more samples
- The samples deviated by ±4 SDs from the population average based on the first two genetic principal components.

Samples were tagged, should there be evidence of a Mendelian error, or contain replicate samples with over 50,000 discrepancies.

Variants were removed if:

- The variant did not pass the Hardy–Weinberg Equilibrium test (*p*-value < 10^−6^).
- The variant had a call rate <98%

#### Imputation

Pre-phasing was done with Eagle 2.4.1^28^, and imputation of samples was carried out using the SiSu v4.2 imputation reference panel^29^. The reference panel is designed specifically for the Finnish population, consisting of high-coverage (25–30×) whole-genome sequencing data from 8554 Finns and 20,175,454 variants with a minor allele count of at least 3. After imputation, 9,641,808 variants were imputed with good quality (INFO > 0.6).

#### Ancestry assignment

First, the FinnGen samples were integrated with the 1000 Genomes phase 3 dataset^30^. Genetic principal components were calculated using a subset of 180,042 pruned SNPs. Aberrant was used to identify and remove samples that deviated from the main cluster (*N* = 17,133)^31^. A probability of belonging to either a North-Western European or Finnish population was calculated by firstly performing Principal Component Analysis (PCA) in PLINK v2^27^ with individuals belonging to these ancestries from 1000 genomes data. FinnGen samples were then projected onto this PCA space, and Mahalanobis distances were calculated for each sample against each of the two ancestries. Samples were retained if there was ≥95% probability of belonging to the Finnish ancestry cluster (22 individuals removed).

### UK Biobank

#### Genotyping and QC

Two arrays were used to genotype UK Biobank participants^20^. The UK Biobank Lung Exome Variant Evaluation (UKBiLEVE) Axiom array was used to genotype 49,950 participants. The remaining 438,427 participants were genotyped using the Applied Biosystems UK Biobank Axiom Array.

PCA (fastPCA^32^) was performed on the genetic data, and centralized QC on variants was performed on individuals identified to belong to the largest cluster (*N* = 463,844) according to Aberrant—an unsupervised clustering algorithm^31^. Variants were assessed for evidence of allele frequency variation across batch, plate, sex, or array, and genotypes were largely consistent with Hardy–Weinberg Equilibrium expectations (all *p*-value thresholds < 10^−12^). If a variant failed one or more tests within a given batch, it was set to missing. Previous research provides more detailed information on testing^20^.

#### Imputation

For 487,442 individuals, imputation was performed using the IMPUTE4 software^33^. Genetic variation from the Haplotype Reference Consortium (HRC)^34^ and merged UK10K + 1000 Genomes were used as a reference panel^35^. Single-nucleotide polymorphisms (SNPs) were only included in the final imputation if they were present in both reference panels, giving a total of 96,959,328 SNPs.

#### Ancestry assignment

Ancestry assignment uses methodology and scripts from GenoPred (https://opain.github.io/GenoPred/DiverseAncestry.html)^36^. Individuals were stratified into one of five super populations: African (AFR), American (AMR), South Asian (SAS), East Asian (EAS), and European (EUR). The 1000 Genomes data acted as a reference, given the individuals are known to belong to one of the 5 super populations^30^. Only unambiguous SNPs also present in both the HapMap3 consortium^37^ and the imputed UK Biobank data were retained for PCA. SNPs within both the reference (1000 Genomes) and target (UK Biobank) samples underwent QC such that the minor allele frequency (MAF) > 5% (97,172 SNPs removed), variant missingness > 2% (57,916 SNPs removed), and Hardy–Weinberg Equilibrium *p*-value > 1e^−6^ (126,195 SNPs removed). The 467,970 autosomal SNPs remained following QC and were in the intersection of the reference and target samples. Regions with long-range linkage disequilibrium (e.g., inversions, for details see refs. ^20,38^) were excluded, and independent SNPs (SNPs greater than 1000 kb apart and *r*^2^ < 0.2) were retained. PCA was then performed in the reference sample using PLINK v2^27^, and a multinomial elastic-net regression was trained using 5-fold cross-validation, superpopulation as the outcome, and the first 10 PCs as covariates. PCs from the target sample were then projected into the reference space, and a prediction on super population made. Classifications were made according to the superpopulation with the greatest probability. To be classified, the max probability must be over 0.5; it was set to missing (*N* = 4899).

PCA was performed using a random subset of 1000 individuals per superpopulation, and PC’s from the rest of the superpopulation sample were projected onto this space. Distances from the centroid were calculated and outliers removed. Outliers were classified as having a distance > 75 percentile + 30 × Interquartile Range. Following within-ancestry QC, 8414; 1177; 2951; 458,715 and 11,247 individuals were allocated to AFR, AMR, EAS, EUR and SAS super populations, respectively.

### GS

#### Genotyping and QC

GS consists of ~24,000 individuals from across Scotland aged between 18 and 99 years. Phenotypic data were obtained at baseline along with whole blood samples for DNA quantification.

Genotype data were assayed for 20,195 participants in two batches, with 9863 participants in the first batch and the remainder in the second. The genotyping was performed using the Illumina HumanOmniExpressExome-8 v1.0 BeadChip and the Illumina HumanOmniExpressExome-8 v1.2 BeadChip, respectively, comprising 936,957 variants prior to QC. Individuals or SNPs with a low call rate (<98%) and SNPs with MAF < 0.01 and Hardy–Weinberg *p*-value < 1 × 10^−6^ were removed. Mendelian errors were removed by setting the individual-level genotypes at erroneous SNPs to missing. In total, 163 samples and 332,099 variants were removed during QC, resulting in 604,858 variants prior to imputation.

#### Imputation

Genotyped data were imputed using the HRC panel v1^34^ with the positional Burrows-Wheeler transform software (v. 3)^39^ on the Sanger server. Autosomal haplotypes were checked to ensure consistency with the reference panel (strand orientation, reference allele, position). Pre-phasing was performed using Shapeit2 v2r837^40,41^ using the Shapeit2 duohmm option^42^ and cohort family structure in order to improve imputation quality^39^. Variants with low imputation quality (INFO < 0.4), as well as monogenic variants, were removed from the imputed set, resulting in 24,111,857 variants for downstream analysis.

#### Ancestry assignment

Ancestry outliers were removed from the dataset. These were defined as individuals who were more than six SDs away from the mean in a principal component analysis, as performed with ACTA (v. 0.9)^43^, of GS merged with 1092 participants from the 1000 Genomes Project^30^.

### Estimating polygenic scores

PGSs for 18 diseases were sourced from the INTERVENE consortium^22^ and are available via the PGS Catalog^44^ (publication ID PGP000618; score IDs PGS004869–PGS004886, Supplementary Data 3). For AUD, we used the same approach as INTERVENE to create a new PGS based on the ‘drinks per week’ GWAS^24^: (1) we selected SNPs present in HapMap phase 3^37,45^ and 1000 genomes phase 3^30^; (2) SNP weights were calculated with MegaPRS^46^ with the BLD-LDAK heritability model^47^, selecting the optimal PGS tool and hyperparameters (‘mega’ argument, for AUD: LDAK-Ridge with heritability of 0.70); and (3) individual-level PGSs were computed with PLINK (v2.00a5LM)^27,48^ and standardized (mean = 0, SD = 1) within each study.

### Statistics and reproducibility

#### Survival analysis models

We performed Cox proportional hazard (Cox-PH) regression with age as the time scale. Follow-up began at birth and ended with the earliest disease diagnosis (cases), death, last registry follow-up, or age 80. For UK Biobank and GS, we excluded diseases for which those cohorts contributed to the discovery GWAS; in FinnGen, overlapping discovery samples were excluded from analyses (Supplementary Data 3).

All models were adjusted for sex (except for breast and prostate cancer, which were respectively restricted to only female participants or only male participants), birth decade (in education, not occupation models), and genetic principal components (PCs, 5 PCs in models without PGSs, 10 PCs in models with PGSs). Proportional hazards assumptions were tested with scaled Schoenfeld residuals (Supplementary Fig. 1-2).

We evaluated four models for each disease in each study: 1) association of the disease-specific PGS or education/occupation alone with disease risk; 2) association of the disease-specific PGS and education/occupation together; 3) association of the disease-specific PGS stratified by education/occupation; and 4) association of the disease-specific PGS, education/occupation, and their interaction. By comparing models 1 and 2, we assessed the potential overlapping effects between genetic and educational/occupational risk factors. Because differences in the strength or direction of associations can occur even if the main effects are (largely) independent, models 3 and 4 evaluated whether interaction effects were present.

#### Meta-analyses

We conducted fixed- and random-effects meta-analyses of log HR (i.e., beta coefficients) across cohorts with the ‘metafor’ (v. 4.6-0) R package (v4.4.1)^49,50^. Exceptions were AF (education and occupation), colorectal cancer (education and occupation), and skin melanoma (occupation), where discovery GWAS overlap or small case counts precluded analysis in some cohorts, so results are based on FinnGen alone. In the manuscript, we present the results of the fixed-effect meta-analyses, while the results for the random-effects meta-analyses have been provided in the accompanying supplemental figures and data files. To assess heterogeneity across studies, we estimated Cochran’s *Q*-statistic^51^. To test for attenuation of PGS or education/occupation effects after mutual adjustment, we compared effect sizes using two-sided Wald tests. We applied a Bonferroni-adjusted significance threshold of *p* < 2.63 × 10^−03^ to account for 19 outcomes.

#### Cumulative incidence estimation

Cumulative incidence up to age 80 was estimated using age-, sex-, and country-specific incidence, prevalence, and mortality data from the GBD^18^, accounting for the competing risk of death. Following previous approaches^22,52,53^, we applied a piecewise constant hazard model across 5-year age bins to convert incidence rates into probabilities of disease. These were combined with survival probabilities to estimate cumulative incidence.

For PGS-stratified incidence estimates, we used Cox-PH models in FinnGen to estimate HRs across five PGS groups (<20%, 20–40%, 40–60% [reference], 60–95%, >95%), stratified by education/occupation. These HRs were then applied to GBD-derived baseline rates. Confidence intervals (CIs) were derived using bootstrapping, and age-specific HRs were interpolated across age quartiles as needed. Cumulative incidence estimations were only generated for outcomes where a significant interaction between the disease-specific PGS and educational attainment was observed in the FinnGen.

#### Evaluating predictive performance

In a random 80% training subset of FinnGen participants, we fitted five Cox-PH models: (1) a baseline model including basic covariates (2 models were run, both included sex [except for breast and prostate cancer], birth year and the first 5 genetic PCs, the second model also included genetic PCs 6-10); (2) the baseline model including all 10 genetic PCs plus the disease-specific PGS; (3) the baseline model including the first 5 genetic PCs plus educational attainment; (4) the baseline model including all 10 genetic PCs plus the disease-specific PGS and educational attainment; and (5) the baseline model including all 10 genetic PCs plus the disease-specific PGS, educational attainment, and their interaction. Model performance for predicting case status by the end of follow-up was evaluated in the held-out 20% FinnGen test set. To assess generalizability, model coefficients estimated in the FinnGen training set were applied to independent participants in the UK Biobank to generate predicted risks, and predictive performance was evaluated in this cohort without re-estimating model parameters. Discrimination of the models was assessed with the area under the curve (AUC)^54^, continuous net reclassification index (NRI), and continuous integrated discrimination index (IDI)^55^. Here, we made three comparisons of the model performance: (1) compared models with basic covariates to models that also included the disease-specific PGS or educational attainment; (2) compared models with basic covariates, disease-specific PGS, and educational attainment to model that only included the basic covariates and the disease-specific PGS or educational attainment; and (3) compared model with basic covariates, disease-specific PGS, educational attainment, and their interaction with model that did not include the interaction between the disease-specific PGS and educational attainment.

The AUC quantifies a model’s ability to discriminate between cases and controls, where an AUC of 0.5 indicates no discriminative ability (i.e., equivalent to random chance), while an AUC of 1 indicates perfect discrimination. To compare the discriminative performance of the different models, we tested whether their AUCs were significantly different with DeLong’s test^56^. After assessing the differences in the AUC, calculated with the ‘pROC’ (v. 1.18.5) R package (v. 4.5.0)^57^, between the models in 20% of FinnGen, we validated these findings in the UK Biobank.

The NRI assesses whether a more complex model improves the risk classification compared to a less complex reference model. Thus, it measures how often individuals are correctly reclassified as cases or controls in both models. A positive NRI indicates the more complex model improves classification, while negative NRI estimates indicate the more complex model worsens classification as compared to the less complex reference model. In contrast, the IDI measures the overall improvement in risk prediction by comparing the difference in average predicted probabilities between cases and controls between two models. Here, a positive IDI indicates the more complex model has better discrimination ability than the less complex reference model, and a negative IDI indicates the more complex model is worse at distinguishing cases from controls than the less complex reference model. The NRI and IDI were calculated with the ‘nricens’ (v. 1.6) R package (v. 4.5.0)^58^.

### Sensitivity analyses

#### Assessing the robustness of educational attainment assessment on interaction associations

In our main interaction analyses (i.e., models including educational attainment, the disease-specific PGS, and their interaction), we assessed educational attainment as a dichotomous variable based on the optimal cutoff in the FinnGen study (see “Harmonization educational attainment” section). To assess the robustness of this cutoff, we first determined the median ISCED education category in the UK Biobank and GS. The median ISCED education category in the UK Biobank was ISCED 4, and in GS, ISCED 5. As the median was lower in the UK Biobank than in FinnGen and GS, we re-ran the cohort-level Cox-PH interaction models while applying a cohort-specific cutoff in the UK Biobank, i.e., ISCED ≤ 3 (low) vs ISCED ≥ 4 (high). We then performed the fixed- and random-effects meta-analyses using the original cohort-level estimates for FinnGen and GS and the new UK Biobank results. To test for possible attenuation of the disease-specific PGS, education, or interaction effects, we compared effect sizes of the original and new meta-analysis with two-sided Wald tests. We applied a Bonferroni-adjusted significance threshold of *p* < 2.63 × 10^−03^ to account for 19 outcomes. Similarly, we also conducted cohort-level Cox-PH interaction models without dichotomizing educational attainment and compared these meta-analytical estimates to the original dichotomous results.

#### Assessing the influence of delayed entry and mutually exclusive case definitions on interaction associations

In our primary analyses, Cox-PH models were specified with age as the time scale. Follow-up was defined from birth, and disease outcomes were ascertained from nationwide health registries, which may include diagnoses occurring before cohort enrollment. To address potential bias due to inclusion of prevalent cases and differences in timing of cohort entry, we performed sensitivity analyses in FinnGen restricting to incident events occurring after cohort entry (i.e., after donating blood sample for genotyping). In these analyses, follow-up was defined using age as the time scale with delayed entry at age at cohort entry (left truncation), and individuals with a diagnosis of the outcome prior to cohort entry were excluded. Follow-up continued from age at entry until first diagnosis, death, end of registry follow-up, or age 80 years, whichever occurred first. This approach ensures that risk sets are defined conditional on survival and event-free status at cohort entry. We compared these incident-only estimates to the primary analyses with two-sided Wald tests (Bonferroni-adjusted for 19 outcomes) to assess the robustness of the associations and disease-specific PGS by educational attainment interaction effects.

Moreover, to evaluate the influence of potential overlap between disease definitions, we repeated analyses in FinnGen using mutually exclusive case definitions. Individuals with diagnoses for more than one disease were excluded from case groups, such that each individual could contribute as a case to only one disease outcome. We made exceptions for the cancer outcomes, for the specific cancers, we excluded individuals with another non-cancer or specific-cancer diagnosis, but not a ‘any cancer’ diagnosis. This approach reduces potential bias due to shared comorbidity across outcomes. Results from these sensitivity analyses were also compared (two-sided Wald tests, Bonferroni-adjusted for 19 outcomes) with the primary analyses to assess the consistency of the estimated associations and the disease-specific PGS by education interaction effects.

#### Adjustment for educational attainment polygenic score in interaction associations

To assess the extent to which the observed interaction of disease-specific PGSs by educational attainment reflects genetic confounding or shared genetic architecture, we conducted additional analyses in FinnGen accounting for the genetic component of education. Specifically, we included an educational attainment PGS (EA-PGS)^59^ as a covariate and simultaneously modeled interaction terms between the disease-PGSs and both observed educational attainment and the EA-PGS within the same model. This approach allows evaluation of whether modification of disease-PGS effects is attributable to measured socioeconomic context or to genetically correlated variation in education. Effect sizes for educational attainment, the disease-specific PGS, and their interaction were compared with the original FinnGen results with two-sided Wald tests Bonferroni-adjusted for 19 outcomes).

#### Fine-gray competing risk models

For outcomes where a significant interaction between the disease-specific PGS and educational attainment was observed in the fixed-effect meta-analysis (any cancer, asthma, AF, CHD, hip osteoarthritis, knee osteoarthritis, and type 2 diabetes), we performed additional sensitivity analyses using Fine-Gray competing risk models in the FinnGen study^60^. In survival analysis, the presence of competing risks, events such as death from other causes that preclude the event of interest, can bias standard Cox-PH models by overestimating the cumulative incidences. The fine-gray model addresses this by directly modeling the subdistribution hazard, allowing for estimation of the cumulative incidence function of the disease outcome while accounting for the competing risk of death from other causes.

#### Analyses in non-European ancestries

In the UK Biobank, we explored whether the associations observed in individuals of European ancestry for the disease-specific PGS and educational attainment with type 1 diabetes, prostate cancer, gout, rheumatoid arthritis, breast cancer, epilepsy, and AUD replicated in individuals of non-European ancestry (see “Genotyping” section). Given the relatively low sample sizes for non-European ancestries, we only had sufficient cases and controls by education level available for breast and prostate cancer in South Asian (SAS), East Asian, and African individuals, and for gout, rheumatoid arthritis, and epilepsy only in SAS individuals. All other trait-ancestry combinations could not be assessed.

### Ethical approval

Study subjects in FinnGen provided (written) informed consent for biobank research, based on the Finnish Biobank Act. Alternatively, separate research cohorts, collected prior to the Finnish Biobank Act coming into effect (in September 2013) and the start of FinnGen (August 2017), were collected based on study-specific consents and later transferred to the Finnish biobanks after approval by Fimea (Finnish Medicines Agency), the National Supervisory Authority for Welfare and Health. Recruitment protocols followed the biobank protocols approved by Fimea. The Coordinating Ethics Committee of the Hospital District of Helsinki and Uusimaa (HUS) statement number for the FinnGen study is Nr HUS/990/2017.

The FinnGen study is approved by Finnish Institute for Health and Welfare (permit numbers: THL/2031/6.02.00/2017, THL/1101/5.05.00/2017, THL/341/6.02.00/2018, THL/2222/6.02.00/2018, THL/283/6.02.00/2019, THL/1721/5.05.00/2019 and THL/1524/5.05.00/2020), Digital and population data service agency (permit numbers: VRK43431/2017-3, VRK/6909/2018-3, VRK/4415/2019-3), the Social Insurance Institution (permit numbers: KELA 58/522/2017, KELA 131/522/2018, KELA 70/522/2019, KELA 98/522/2019, KELA 134/522/2019, KELA 138/522/2019, KELA 2/522/2020, KELA 16/522/2020), Findata permit numbers THL/2364/14.02/2020, THL/4055/14.06.00/2020, THL/3433/14.06.00/2020, THL/4432/14.06/2020, THL/5189/14.06/2020, THL/5894/14.06.00/2020, THL/6619/14.06.00/2020, THL/209/14.06.00/2021, THL/688/14.06.00/2021, THL/1284/14.06.00/2021, THL/1965/14.06.00/2021, THL/5546/14.02.00/2020, THL/2658/14.06.00/2021, THL/4235/14.06.00/2021, Statistics Finland (permit numbers: TK-53-1041-17 and TK/143/07.03.00/2020 (earlier TK-53-90-20) TK/1735/07.03.00/2021, TK/3112/07.03.00/2021) and Finnish Registry for Kidney Diseases permission/extract from the meeting minutes on 4th July 2019.

The Biobank Access Decisions for FinnGen samples and data utilized in FinnGen Data Freeze 11 include: THL Biobank BB2017_55, BB2017_111, BB2018_19, BB_2018_34, BB_2018_67, BB2018_71, BB2019_7, BB2019_8, BB2019_26, BB2020_1, BB2021_65, Finnish Red Cross Blood Service Biobank 7.12.2017, Helsinki Biobank HUS/359/2017, HUS/248/2020, HUS/430/2021 §28, §29, HUS/150/2022 §12, §13, §14, §15, §16, §17, §18, §23, §58, §59, HUS/128/2023 §18, Auria Biobank AB17-5154 and amendment #1 (August 17 2020) and amendments BB_2021-0140, BB_2021-0156 (August 26 2021, Feb 2 2022), BB_2021-0169, BB_2021-0179, BB_2021-0161, AB20-5926 and amendment #1 (April 23 2020) and it´s modifications (Sep 22 2021), BB_2022-0262, BB_2022-0256, Biobank Borealis of Northern Finland_2017_1013, 2021_5010, 2021_5010 Amendment, 2021_5018, 2021_5018 Amendment, 2021_5015, 2021_5015 Amendment, 2021_5015 Amendment_2, 2021_5023, 2021_5023 Amendment, 2021_5023 Amendment_2, 2021_5017, 2021_5017 Amendment, 2022_6001, 2022_6001 Amendment, 2022_6006 Amendment, 2022_6006 Amendment, 2022_6006 Amendment_2, BB22-0067, 2022_0262, 2022_0262 Amendment, Biobank of Eastern Finland 1186/2018 and amendment 22§/2020, 53§/2021, 13§/2022, 14§/2022, 15§/2022, 27§/2022, 28§/2022, 29§/2022, 33§/2022, 35§/2022, 36§/2022, 37§/2022, 39§/2022, 7§/2023, 32§/2023, 33§/2023, 34§/2023, 35§/2023, 36§/2023, 37§/2023, 38§/2023, 39§/2023, 40§/2023, 41§/2023, Finnish Clinical Biobank Tampere MH0004 and amendments (21.02.2020 & 06.10.2020), BB2021-0140 8§/2021, 9§/2021, §9/2022, §10/2022, §12/2022, 13§/2022, §20/2022, §21/2022, §22/2022, §23/2022, 28§/2022, 29§/2022, 30§/2022, 31§/2022, 32§/2022, 38§/2022, 40§/2022, 42§/2022, 1§/2023, Central Finland Biobank 1-2017, BB_2021-0161, BB_2021-0169, BB_2021-0179, BB_2021-0170, BB_2022-0256, BB_2022-0262, BB22-0067, Decision allowing to continue data processing until 31st Aug 2024 for projects: BB_2021-0179, BB22-0067,BB_2022-0262, BB_2021-0170, BB_2021-0164, BB_2021-0161, and BB_2021-0169, and Terveystalo Biobank STB 2018001 and amendment 25th Aug 2020, Finnish Hematological Registry and Clinical Biobank decision 18th June 2021, Arctic biobank P0844: ARC_2021_1001.

Ethics approval for the UK Biobank study was obtained from the North West Centre for Research Ethics Committee (11/NW/0382). UK Biobank data used in this study were obtained under approved application 78537. Ethical approval for the GS:SFHS study was obtained from the Tayside Committee on Medical Research Ethics (on behalf of the National Health Service; REC Reference Number: 05/S1401/89).

## Results

### Association of PGSs, education, and their interaction with 19 diseases

We harmonized disease and education definitions across FinnGen (*N* = 360,663), UK biobank (*N* = 353,294 European and 13,266 non-European ancestry), and GS (*N* = 15,971), and calculated PGSs using a previously reported pipeline^22^ (see “Methods” section). Unless otherwise specified, all results are provided for participants of European ancestry. Descriptive statistics for participants of European ancestry are shown in Supplementary Data 4.

**Fig. 1:**
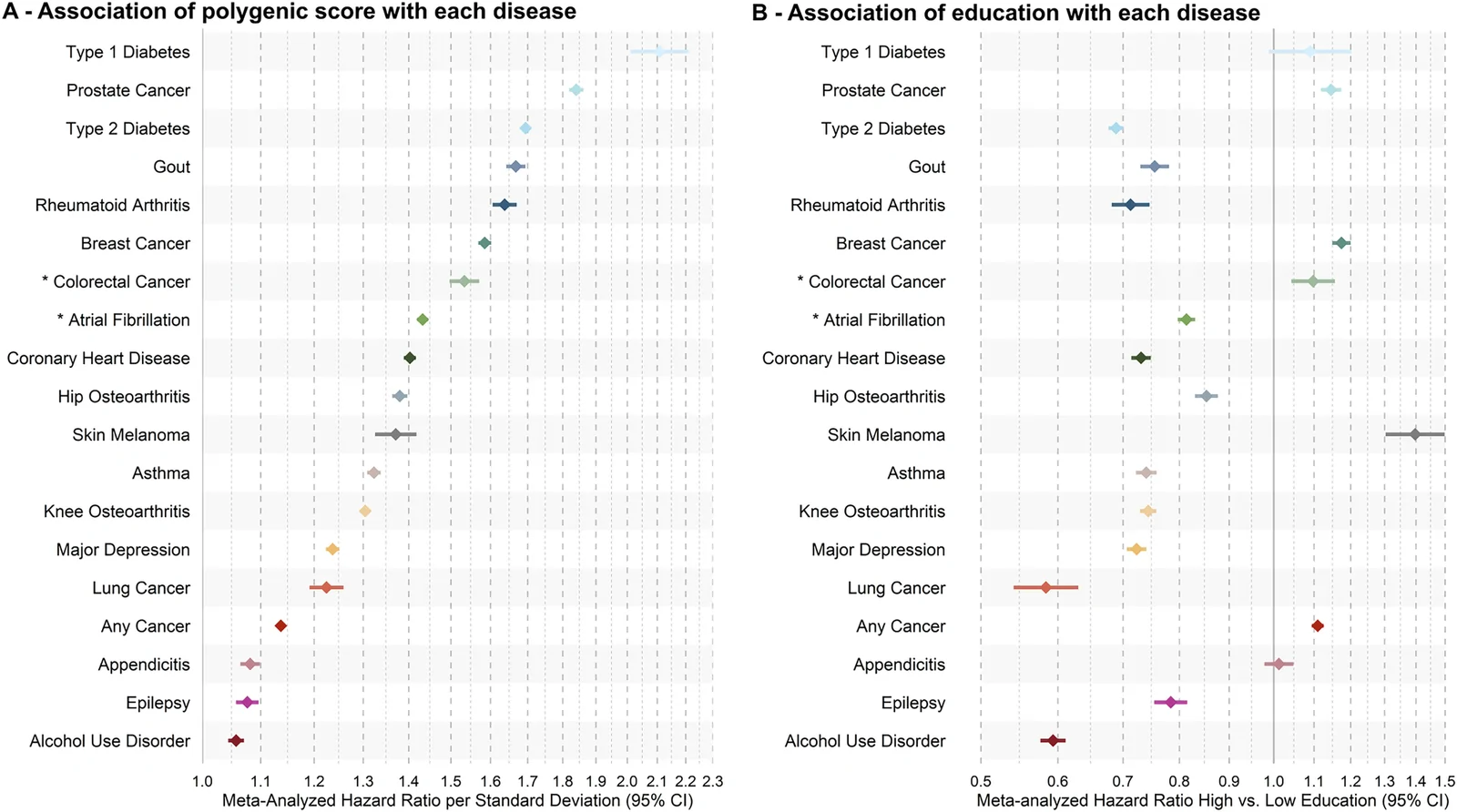
Meta-analyzed HR for the relative risk of educational attainment and disease-specific polygenic scores on 19 complex diseases. **A** Fixed-effects meta-analysis of the log of the HR per SD with 95% CIs of the disease-specific polygenic scores on risk of complex diseases. **B** Fixed-effects meta-analysis of the log of the HR with 95% CIs for individuals with high education relative to individuals with low education on risk of complex diseases. The asterisk indicates the complex disease was not meta-analyzed and assessed only in the FinnGen study. The exact values are in Supplementary Data 5. Sample sizes by case-control status and educational attainment for each complex disease in each biobank study are in Supplementary Data 4.

We evaluated the association between PGSs, education, and their interaction with 19 diseases with Cox-PH models in each study, followed by fixed-effect meta-analysis across the studies. All PGSs were significantly associated with their respective diseases (Fig. 1A and Supplementary Data 5), with HR per 1 SD of PGS ranging from 2.11 (95% CI: 2.01–2.21) for type 1 diabetes (T1D) to 1.06 (95% CI: 1.04–1.07) for AUD. Associations were largely consistent across cohorts, though we observed heterogeneity for several diseases, including knee osteoarthritis (*Q* = 11.97, *p* = 5.40 × 10^−04^), T2D (*Q* = 13.61, *p* = 2.25 × 10^−04^), prostate cancer (*Q* = 16.67, *p* = 2.41 × 10^−04^), gout (*Q* = 44.78, *p* = 2.21 × 10^−11^), asthma (*Q* = 45.28, *p* = 1.70 × 10^−11^), and rheumatoid arthritis (RA, *Q* = 60.43, *p* = 7.62 × 10^−15^), based on Cochran’s *Q* test (Bonferroni-adjusted *p* < 2.63 × 10^−03^, Supplementary Data 5 and Supplementary Fig. 3). Associations were not attenuated after adjustment for high education (Supplementary Data 5–7, Supplementary Figs. 3, 4A, and 5).

High education was associated with lower risk for 12 diseases, including T2D, CHD, asthma, and osteoarthritis (HR range, 0.58–0.85 [95% CI range, 0.54–0.88]), and with increased risk for skin melanoma, breast, colorectal, prostate, and any cancer (HR range, 1.10–1.40 [95% CI range, 1.04–1.50]), and was not associated with risks of T1D or appendicitis (Fig. 1A and Supplementary Data 5). These associations also showed heterogeneity for T2D (*Q* = 13.44, *p* = 2.46 × 10^−04^), prostate cancer (*Q* = 15.45, *p* = 4.41 × 10^−04^), gout (*Q* = 19.96, *p* = 7.91 × 10^−06^), AUD (*Q* = 55.59, *p* = 8.49 × 10^−13^), and breast cancer (*Q* = 80.16, *p* = 3.93 × 10^−18^, Supplementary Data 5 and Supplementary Fig. 3). Adjusting for PGSs had minimal impact on education associations, except for T2D (HR difference = 0.03, *p* = 0.0001; Fig. 1B, Supplementary Data 5–7, Supplementary Fig. 3, 4B, and 5), suggesting independent contributions of genetic risk and education to disease risk.

**Fig. 2:**
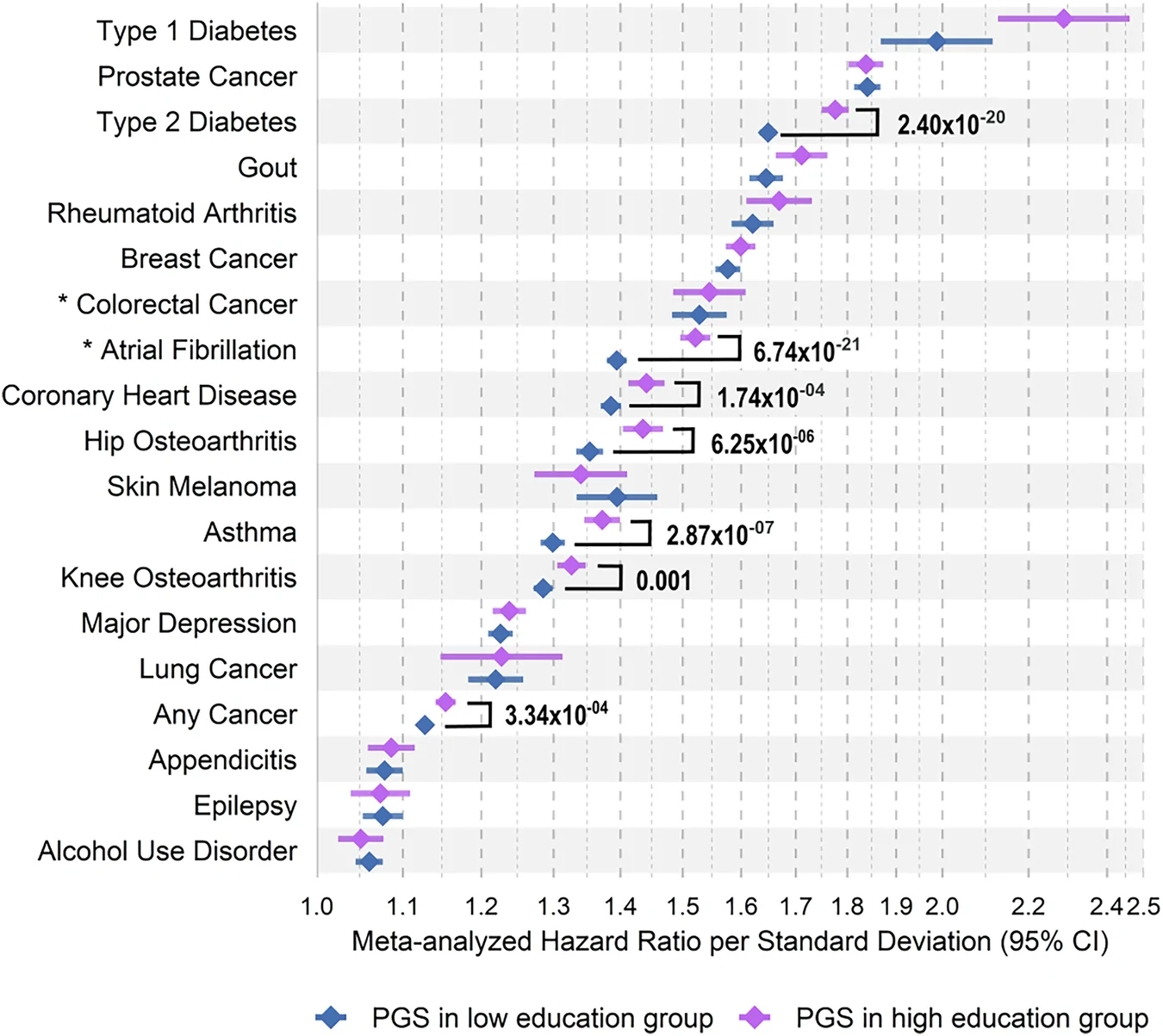
Meta-analyzed HR for the relative risk of the disease-specific polygenic scores by educational attainment on 19 complex diseases. Fixed-effects meta-analysis of the log HR per SD with 95% CIs (95% CI) of the disease-specific polygenic score in the low (blue) or high (purple) education group. The asterisk indicates the complex disease was not meta-analyzed and assessed only in the FinnGen study. Significance of the differences in the effect of the disease-specific polygenic score per education group evaluated by the statistical significance of the interaction term between the disease-specific PGS and education level (obtained from fixed-effects meta-analysis of the log HR, see “Methods” section) after Bonferroni correction for multiple testing of 19 outcomes (*p* < 2.63 × 10^−03^). The exact values are in Supplementary Data 8 and 9. Sample sizes by case-control status and educational attainment for each complex disease in each biobank are in Supplementary Data 4.

We next estimated the association of PGSs by stratifying on education level and found that for seven diseases, including AF, T2D, CHD, osteoarthritis, asthma, and any cancer, PGS effect sizes were larger among individuals with high education (Bonferroni-adjusted *p* < 2.63 × 10^−03^; Fig. 2, Supplementary Data 8 and 9). For example, for AF, the PGS HRs per SD were 1.39 (95% CI: 1.38–1.41) among individuals with low education and 1.52 (95% CI: 1.50–1.55) among those with high education. For T2D, HRs were 1.65 (95% CI: 1.63–1.66) and 1.78 (95% CI: 1.75–1.80), respectively. Heterogeneity across cohorts in these stratified analyses was evident in the low education group for asthma (*Q* = 26.65, *p* = 2.43 × 10^−07^), prostate cancer (*Q* = 26.88, *p* = 1.46 × 10^−06^), gout (*Q* = 32.11, *p* = 1.45 × 10^−08^), and RA (*Q* = 46.08, *p* = 1.13 × 10^−11^), and in the high education group for gout (*Q* = 9.92, *p* = 1.64 × 10^−03^), appendicitis (*Q* = 10.75, *p* = 1.04 × 10^−03^), RA (*Q* = 13.98, *p* = 1.85 × 10^−04^), and asthma (*Q* = 21.87, *p* = 2.91 × 10^−06^, Supplementary Data 8 and Supplementary Fig. 6).

### Cumulative incidence estimation stratified by PGS and education level

**Fig. 3:**
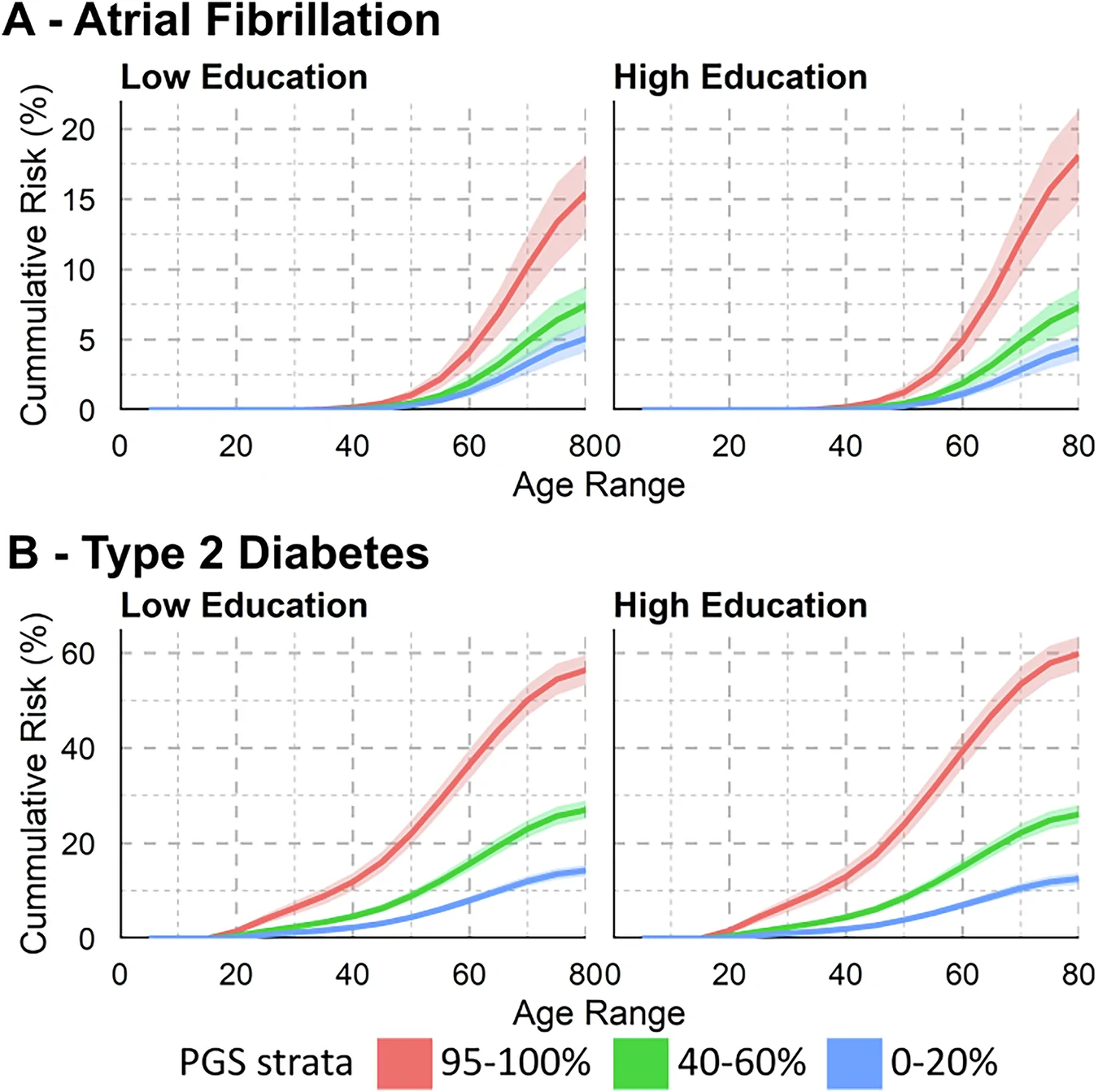
Educational attainment-specific cumulative incidence estimates in FinnGen. Bootstrapped 95% CIs reflect the uncertainty of the cumulative incidence estimates for the top, median, and bottom of the disease-specific PGS distribution for **A** AF, and **B** type 2 diabetes. The exact values are in Supplementary Data 12, the exact values of the underlying Cox-PH model are in Supplementary Data 11, and the sample sizes by case-control status and educational attainment are in Supplementary Data 10.

For each disease for which we observed a significant interaction between PGSs and education in FinnGen, we derived Finnish-specific estimates of the cumulative incidence by PGS quantiles, stratified by education level, where the baseline risk was calibrated using the GBD^18^. Cumulative incidence curves demonstrated that disease risk increased with higher PGS levels and differed by education (Fig. 3, Supplementary Data 10–12, and Supplementary Fig. 7). For instance, individuals in the top 5% of the PGS distribution for AF or T2D with high education had higher cumulative incidence by age 80 compared to those with low education (Fig. 3, AF: 18.09% [95% CI: 14.79–21.36%] vs 15.41% [95% CI: 12.61–18.16%], T2D: 59.83% [95% CI: 56.31–63.47%] vs 56.44% [95% CI: 53.32–59.62%]).

### Predictive value of PGSs, education, and their interaction on disease risk

**Fig. 4:**
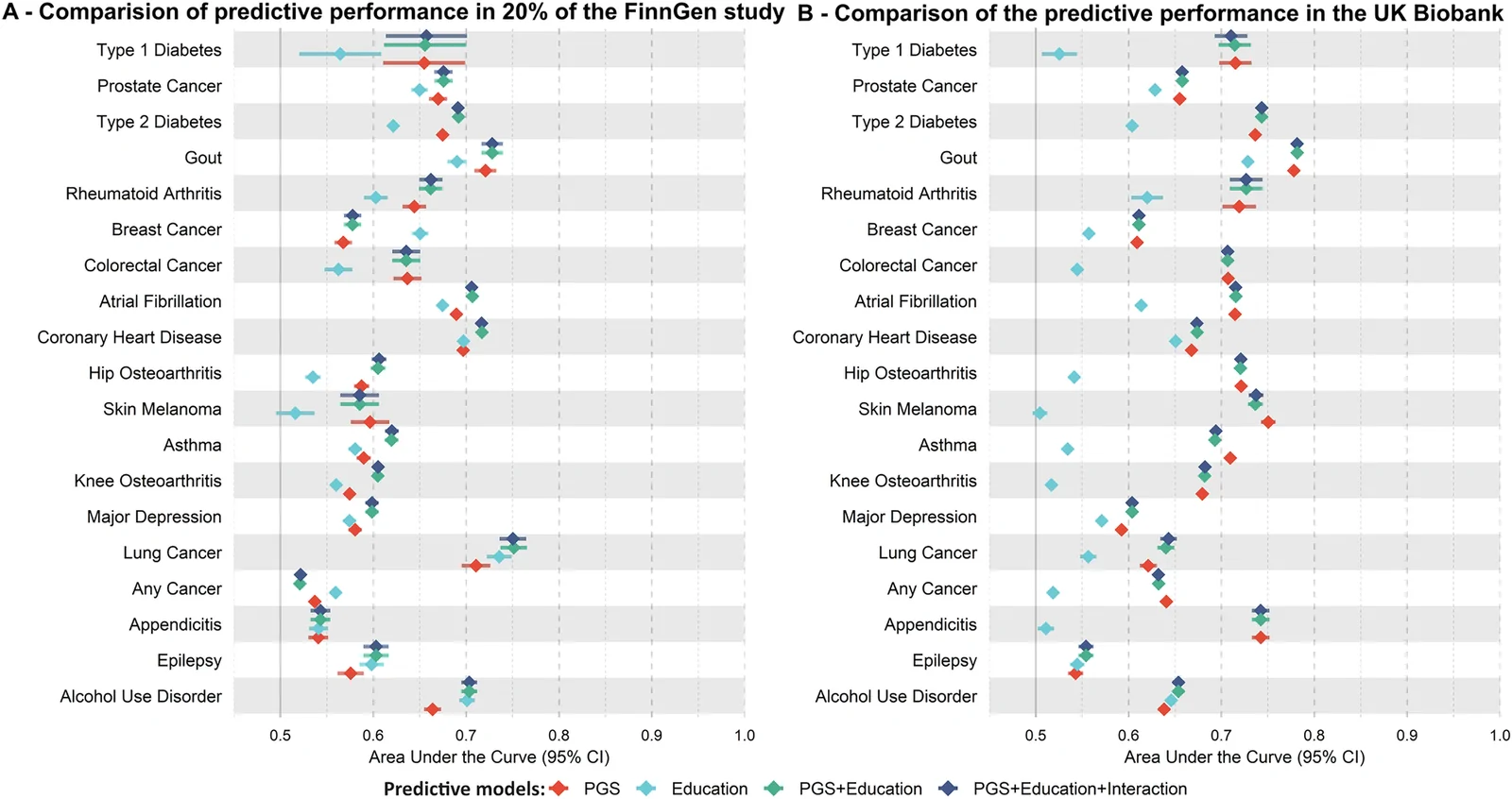
Predictive performance of disease-specific polygenic scores, educational attainment, and their combination. AUC with 95% CI (95% CI) are shown for predictive models in 20% of the FinnGen study (**A**) and the UK Biobank (**B**). Models include: (1) disease-specific polygenic score (PGS) only, (2) educational attainment (EA) only, (3) disease-specific PGS + EA, and (4) disease-specific PGS + EA + interaction of disease-specific PGS and EA. AUCs were estimated from Cox-PH models trained in 80% of the FinnGen study (see “Methods” section, Supplementary Data 14–17). Descriptive statistics by case-control status and educational attainment for each complex disease in 20% of the FinnGen study and all UK Biobank traits are in Supplementary Data 18. Full model estimates, including covariate-only baselines, are reported in Supplementary Data 19.

To assess the added predictive value of combining genetic and education information, we trained Cox-PH models in FinnGen and evaluated model performance in held-out data, with replication in the UK Biobank (see “Methods” section). In FinnGen, models including both PGS and education outperformed models with education alone for all diseases except breast or any cancer, while the combined model outperformed PGS alone models for 16 out of the 19 diseases (Fig. 4 and Supplementary Data 13–19). For example, in AF, education and PGS together had an AUC of 0.707 (95% CI: 0.701–0.713), compared to 0.689 (95% CI: 0.683–0.696) for PGS alone and 0.675 (95% CI: 0.669–0.680) for education alone. Although absolute improvements in AUC were modest (e.g., +0.017 for PGS + education vs PGS alone in AF), they were consistent across most diseases and confirmed by continuous net reclassification and integrated discrimination improvement metrics (Supplementary Data 20). We also observed improved prediction of the combined PGS and education model compared to education only in the UK Biobank for all studied diseases, though the combined model outperformed PGS-only models for fewer diseases in the UK Biobank (12 out of 19). Adding a PGS by education interaction term provided no predictive benefit across diseases in either FinnGen or the UK Biobank. For instance, in FinnGen, the AUC for AF was 0.706 (95% CI: 0.700–0.712) with and 0.707 (95% CI: 0.701–0.713) without the interaction term. Together, these findings indicate that education contributes complementary, though incremental, predictive information beyond polygenetic risk for most complex diseases.

### Occupation, PGSs, and disease risk across 19 diseases

We next examined occupation as an alternative socioeconomic indicator by modeling its association with 19 diseases alongside disease-specific PGSs and their interaction, using Cox-PH models in FinnGen, the UK Biobank, and GS, followed by fixed-effect meta-analysis. Consistent with analyses of educational attainment, all PGSs were significantly associated with their respective diseases (Supplementary Fig. 8 and Supplementary Data 21 and 22). Evidence of between-cohort heterogeneity was observed for gout (*Q* = 45.85, *p* = 1.27 × 10^−11^), asthma (*Q* = 9.78, *p* = 1.77 × 10^−03^), RA (*Q* = 47.57, *p* = 5.32 × 10^−12^), and major depression (*Q* = 11.44, *p* = 7.18 × 10^−04^. Adjustments for occupation did not attenuate PGS associations (Supplementary Fig. 9 and Supplementary Data 21–24), which were also comparable to those observed in the educational attainment analyses (Supplementary Figs. 10 and 11, and Supplementary Data 25).

Upper-level occupation was associated with lower risk for 9 diseases, including T2D, CHD, RA, and lung cancer (HR range, 0.77–0.93 [95% CI: 0.69–0.97]), and with higher risk for 7 diseases, including skin melanoma, breast, colorectal, prostate, and any cancer (HR range, 1.11–1.72 [95% CI: 1.06–1.91]), while no associations were observed for T1D, asthma or hip osteoarthritis (Supplementary Fig. 8, and Supplementary Data 21 and 22). These associations showed evidence of heterogeneity for prostate cancer (*Q* = 38.63, *p* = 4.09 × 10^−09^), epilepsy (*Q* = 36.24, *p* = 1.75 × 10^−09^), AUD (*Q* = 51.92, *p* = 5.32 × 10^−12^), and breast cancer (*Q* = 95.11, *p* = 2.22 × 10^−21^). As in the education-based analyses, additional adjustment for PGSs had minimal impact on occupational associations (Supplementary Fig. 9 and Supplementary Data 21–24). However, effect estimates differed from those based on educational attainment for 13 out of 19 diseases, generally reflecting attenuation of protective associations and stronger risk-increasing effects (Supplementary Figs. 10 and 11, and Supplementary Data 25). Notably, whereas higher educational attainment was associated with reduced risk of major depression, upper-level occupation was associated with increased risk.

Stratified analyses showed no evidence of differential PGS effects between lower- and upper-level occupation groups (Supplementary Fig. 12, and Supplementary Data 26 and 27). Moreover, PGS associations within occupational strata closely resembled those observed across educational strata, with similar effect sizes for most diseases (Supplementary Figs. 10 and 11, and Supplementary Data 28).

For diseases showing significant PGS by education interactions in FinnGen, we additionally estimated cumulative incidence across PGS quantiles stratified by occupation using Finnish-specific baseline risks from the GBD^18^. These analyses revealed qualitatively similar patterns to those observed for education, with risk increasing across PGS strata and modest differences by occupation, although effect differences were generally smaller (Supplementary Fig. 13 and Supplementary Data 29–31).

### Sensitivity analyses

To assess the robustness of our findings for educational attainment, we conducted several sets of sensitivity analyses. First, to assess the robustness of our dichotomized educational attainment assessment on the interaction associations, we first re-ran our meta-analyses after determining cohort-specific cutoffs (see “Methods” section). By applying cohort-specific cutoffs, we observed significant differences in the interaction associations for AUD, RA, gout, T1D, breast and prostate cancer, as well as in the main education effect for AUD, epilepsy, RA, gout, T1D, breast and prostate cancer (Supplementary Fig. 14 and Supplementary Data 32–34). Across these traits, the education and interaction associations were stronger after applying the cohort-specific threshold. Second, we also compared the interaction associations with meta-analytical models in which education was not dichotomized (see “Methods” section). By not dichotomizing education, we observed significant attenuation in the interaction associations for any cancer, hip and knee osteoarthritis, asthma, CHD, AF, and T2D, as well as in the main education association for all traits except for T1D and appendicitis, and in the main disease-specific PGS association for AF and T2D (Supplementary Fig. 15, and Supplementary Data 35 and 36).

Third, to address potential bias from prevalent cases and cohort entry timing on the interaction associations, we restricted analyses in FinnGen to incident events occurring after cohort entry (see “Methods” section). While we generally observed small attenuation of effect sizes in the interaction, as well as for the main education and disease-specific associations, the interaction associations were not significantly different when accounting for case prevalence and cohort entry timing (Supplementary Fig. 16 and Supplementary Data 37–39). Fourth, to minimize bias from shared comorbidity across outcomes, we restricted analyses in FinnGen to mutually exclusive case definitions (see “Methods” section). By excluding mutually exclusive case definitions, we observed no significant differences in the interaction associations. However, the associations of educational attainment with AUD and AF and of the disease-specific PGS for AF and T2D did differ significantly (Supplementary Fig. 17 and Supplementary Data 40–42). Fifth, when accounting for the EA-PGS and its interaction with disease-specific PGSs in FinnGen, neither the interaction associations nor the associations for the main education or disease-specific PGSs changed (Supplementary Fig. 18 and Supplementary Data 43 and 44).

Sixth, sensitivity analyses in FinnGen tested the robustness of the significant interaction findings by accounting for non-cause-specific mortality as a competing risk. The interaction associations remained unchanged, though the main effects of PGS and education were modestly attenuated (Supplementary Fig. 19 and Supplementary Data 9, 45–47). Finally, in the UK Biobank, we compared results between individuals of European and non-European ancestry. PGS effects were generally consistent across ancestries, while education showed inconsistent associations in non-European ancestries, with wide CIs (Supplementary Fig. 20 and Supplementary Data 5, 48 and 49).

## Discussion

In this study, we assessed the association between SES, using educational attainment and occupation as proxies, and PGSs for 19 complex diseases on the risk of these diseases and evaluated their predictive ability using data from 743,194 study participants across three biobank studies from Finland and the United Kingdom. We quantified the independent associations of both SES and PGSs with the 19 common diseases. We showed that for CHD, T2D, AF, osteoarthritis, asthma, and any cancer, the association of disease-specific PGSs varies by education, but not occupation, level, with stronger PGS associations in the higher education group. Additionally, combining PGSs and educational attainment improved disease prediction compared to using either factor alone for most diseases, but their interaction did not enhance prediction models.

These findings allow us to draw several conclusions. First, SES, as measured by educational attainment and occupation, and PGSs are both strongly associated with disease risk across many common diseases. This is consistent with previous work linking lower SES to increased risk of premature mortality^61^ and chronic diseases such as cardiovascular disease^62,63^, and higher SES to increased incidences of many types of cancer, including prostate cancer^64^, potentially reflecting longer life expectancies^65^, which increases exposure to age-related cancer risk^66^, as well as differential screening and detection practices^67^. Second, our findings show that both PGSs and SES are independently associated with the risk of most diseases. Third, the association of PGSs with disease risk varies across educational strata, consistent with previous findings for CHD^68,69^, suggesting that genetic risk is not uniformly expressed across populations but may depend on contextual factors, including SES, lifestyle, and environmental exposures. Finally, integrating genetic and education information modestly improves disease prediction, highlighting their complementary roles in stratifying disease risk.

The magnitude of predictive improvement and the relative contribution of education varied across cohorts, with somewhat greater gains observed in FinnGen compared to the UK Biobank. These differences may reflect variation in cohort composition, including the “healthy volunteer” selection profile of the UK Biobank^70^, which may attenuate socioeconomic gradients, as well as differences in outcome ascertainment and baseline disease risk. In addition, although educational attainment was harmonized using ISCED categories, the dichotomization threshold was defined based on the FinnGen distribution and applied across cohorts, which may reduce sensitivity to socioeconomic variation in the UK Biobank. Consistent with this, applying cohort-specific thresholds resulted in differences in interaction estimates, although the overall conclusions remained robust.

Several mechanisms may underlie the observed variation in genetic risk across socioeconomic strata. Individuals with lower SES tend to encounter more challenging environmental and social circumstances, potentially magnifying their genetic susceptibility to disease due to increased exposure to stress^71,72^, poorer access to healthcare, and limited health-promoting resources^73,74^. For example, SES-related differences in behaviors such as smoking and alcohol consumption contribute to many diseases, including CHD^75^ and T2D^76^, and have been shown to modify the effects of genetic risk^16,17^. Conversely, individuals with higher SES are more likely to access health-promoting resources, adopt healthier lifestyles^73,74^, have greater health literacy^77,78^, and make more use of preventive healthcare^67,73,74^, which can mitigate genetic risk. At the same time, these differences in healthcare utilization may also influence disease detection. Individuals with higher SES are more likely to participate in screening programs and undergo more frequent or earlier diagnostic evaluations, which may increase the likelihood of disease detection independent of underlying biological risk^79–81^. As a result, some of the observed differences in hazard across educational strata may partly reflect differential ascertainment rather than true effect modification, particularly for conditions with substantial diagnostic discretion. While many of the outcomes in our study are primarily based on hospital and registry data, which may reduce such biases, differential detection cannot be fully excluded and should be considered when interpreting these findings.

Importantly, our primary analyses are conducted on the relative scale (hazard ratios (HR)), and differences in baseline risk across educational strata may contribute to the observed interaction patterns. For diseases with lower baseline risk among individuals with higher education, a similar absolute increase in risk associated with genetic predisposition may translate into a larger relative effect estimate. This could partly explain why stronger PGS associations are observed in higher-education groups. However, cumulative incidence analyses showed similar qualitative patterns across educational strata, suggesting that the observed interactions are not solely driven by scaling differences, although effects on the absolute scale were generally smaller.

Interpretation of these findings is further complicated by the fact that educational attainment itself is a genetically influenced trait^14,59,82^. As such, we cannot fully exclude genetic confounding in the observed PGS by education interactions. Although these interactions were robust to additional adjustment for an educational attainment PGS and its interaction with the disease-specific PGS, this approach does not fully disentangle environmental from genetic sources of effect modification. Educational attainment PGSs capture not only direct genetic influences, but also gene-environment correlations, whereby individuals with higher PGSs are more likely to experience favorable social and environmental conditions^83^. In addition, parental genotypes can shape offspring environments through genetic nurture (or dynastic effects), whereby parental alleles influence offspring outcomes via environmental pathways independent of direct genetic transmission^84,85^. Together, these processes suggest that the observed effect modification by education may reflect a combination of environmental stratification and shared genetic architecture, rather than a purely environmental interaction, and should therefore be interpreted with appropriate caution.

Future work aimed at strengthening causal inference could leverage genetically informed family-based designs, such as within-family models, which can help separate direct genetic effects from genetic nurture and shared environmental influences^86^. Extensions incorporating quasi-experimental variation in educational exposure^87^, for example, policy reforms, or longitudinal designs with time-varying SES measures^88^ may further clarify the extent to which SES modifies genetic risk through environmental pathways. While SES contributes to the identification of high-risk individuals, its causal role remains uncertain and should be carefully considered when translating these findings into clinical or public health applications.

Our study has some limitations. First, our results were primarily based on European ancestry individuals living in Europe and need to be more extensively tested in non-European countries and more diverse genetic ancestries to evaluate generalizability across diverse populations^89^. This is particularly relevant for countries with significant immigrant populations with non-European ancestry, as PGSs are less developed for non-Europeans^9^ but also as immigrants are often exposed to increased stress and adversities, regardless of their educational attainment^90^. However, while our results in non-European ancestry individuals in the UKB were mostly in line with our observations in European ancestry individuals, further studies are needed to ensure the portability of PGSs developed in European ancestry individuals to non-European ancestries^91,92^. Second, while we cover two different health care and educational systems, Finland and the UK are relatively equal in their health care access, with both countries having nationalized publicly available health care^93,94^, and universal educational access^95^. The dynamics between education and polygenic risk may be different in countries with varying degrees of healthcare coverage and educational accessibility^96,97^. Third, we primarily used educational attainment as our measure of SES, as it is relatively simple to standardize across countries and educational systems^25^. However, we dichotomized education into high vs low attainment, which may oversimplify the spectrum of educational experiences and obscure more nuanced associations with polygenic risk. While we observed similar results when analyzing occupation, different socioeconomic indices, such as income or neighborhood deprivation, may capture other facets of socioeconomic environments^98^, providing insight into the overlapping and unique aspects of the socioeconomic environment and how they might influence disease risk. Thus, our results are to be interpreted in the context of our operationalization of educational attainment and occupation as proxies for SES.

In summary, both genetic and socioeconomic factors are independently associated with disease risk, with evidence that their effects may vary across social strata. Integrating these factors modestly improves risk prediction, and the gains are context-dependent. Caution is warranted in extrapolating these results to claims about broader health equity, and future efforts should prioritize more diverse populations to ensure generalizability across different socioeconomic groups. While socioeconomic indicators such as educational attainment and occupation are useful for population-level risk stratification, their potential joint use to guide individual clinical decisions needs further studies to ensure that they will not worsen health inequities or contribute to stigmatization.

## Supplementary information

Transparent Peer Review file

Supplemental Information

Description of Additional Supplementary Files

Supplementary Data 1

Supplementary Data 2

Supplementary Data 3

Supplementary Data 4

Supplementary Data 5

Supplementary Data 6

Supplementary Data 7

Supplementary Data 8

Supplementary Data 9

Supplementary Data 10

Supplementary Data 11

Supplementary Data 12

Supplementary Data 13

Supplementary Data 14

Supplementary Data 15

Supplementary Data 16

Supplementary Data 17

Supplementary Data 18

Supplementary Data 19

Supplementary Data 20

Supplementary Data 21

Supplementary Data 22

Supplementary Data 23

Supplementary Data 24

Supplementary Data 25

Supplementary Data 26

Supplementary Data 27

Supplementary Data 28

Supplementary Data 29

Supplementary Data 30

Supplementary Data 31

Supplementary Data 32

Supplementary Data 33

Supplementary Data 34

Supplementary Data 35

Supplementary Data 36

Supplementary Data 37

Supplementary Data 38

Supplementary Data 39

Supplementary Data 40

Supplementary Data 41

Supplementary Data 42

Supplementary Data 43

Supplementary Data 44

Supplementary Data 45

Supplementary Data 46

Supplementary Data 47

Supplementary Data 48

Supplementary Data 49

## Data Availability

The raw individual-level data are protected and are not publicly available due to data privacy laws, but they can be accessed through individual participating biobanks. The Finnish biobank data can be accessed through the Fingenious® services (https://site.fingenious.fi/en/) managed by FINBB. UK Biobank data are available through a procedure described at http://www.ukbiobank.ac. GS data may be accessed through an application process described here: https://www.ed.ac.uk/generationscotland/for-researchers/access. The GWAS data used in this study are available in the GWAS catalog database under accession codes listed in Supplementary Data 1. The PGS scores used in this study are available in the PGS Catalog under publication ID: PGP000618 and score IDs: PGS004869-PGS004886. The GBD (2019)^18^ data are publicly available (https://vizhub.healthdata.org/gbd-results/) and available via the GitHub repository of Jermy et al. (2024)^22,99^. All other data generated during this study are included in this published article and its supplementary files.

## Code availability

Polygenic score weights were computed with MegaPRS (https://dougspeed.com/megaprs/)^46^, and individual-level PGSs were computed with PLINK v2 (https://www.cog-genomics.org/plink/2.0/)^27,48^. All analyses were conducted in R v. 4.5.0 (https://www.r-project.org/)^50^, using the ‘survival’ v. 3.7.0 R package (https://github.com/therneau/survival) for time-to-event models, the the ‘metafor’ v. 4.6-0 R package (https://www.metafor-project.org/doku.php/metafor)^49^ for meta-analyses, the ‘pROC’ v. 1.18.5 R package (https://xrobin.github.io/pROC/)^57^ for Receiver Operating Characteristic curves, and the ‘nricens’ v. 1.6 R package (https://github.com/cran/nricens/tree/master)^58^ for continuous NRI and continuous IDI analyses. The code used for the analyses is available at https://github.com/intervene-EU-H2020/GxE_SESDisease.
