# Supplementary material for: Association and risk prediction of 19 complex diseases with polygenic scores and socioeconomic status
Source: Commun Med (Lond). 2026 Jun 11;6:503. doi: 10.1038/s43856-026-01716-1 (PMC13616955; doi:10.1038/s43856-026-01716-1)
Supplement: Supplementary file 1 [file 43856_2026_1716_MOESM1_ESM.pdf]

nature portfolio

Peer Review File

Association and risk prediction of 19 complex diseases with polygenic scores and socioeconomic status

Corresponding Author: Professor Samuli Ripatti

This file contains all reviewer reports in order by version, followed by all author rebuttals in order by version.

Version 0:

Reviewer comments:

Reviewer #1

(Remarks to the Author)
Summary
This manuscript evaluates how disease-specific polygenic scores (PGSs) and socioeconomic status (SES), operationalized primarily as educational attainment (high vs low), relate to risk of 19 complex diseases across FinnGen, UK Biobank, and Generation Scotland, with an additional non-European ancestry analysis in UK Biobank. The authors fit Cox proportional hazards models (age as the time scale) and meta-analyze effects across cohorts, testing independent contributions of PGS and education and also PGS×education interaction/stratified effects. They additionally assess whether adding education to PGS improves disease prediction in held-out data, reporting AUC and reclassification metrics.

Overall assessment
This is an interesting and well-powered cross-biobank analysis addressing a broadly relevant question: whether and how SES modifies genetic risk, and whether SES and genetic risk provide complementary information for prediction. Strengths include the multi-cohort design, standardized approach across 19 outcomes, explicit interaction/stratified analyses, and multiple sensitivity checks. The main areas that would benefit from clarification/strengthening are (i) definition of time-at-risk and handling of diagnoses relative to cohort entry, (ii) interpretation and robustness of the education (SES) operationalization, (iii) potential ascertainment/detection differences by education that could mimic effect modification, and (iv) alignment of the prediction modeling with the time-to-event framework.

MAJOR COMMENTS

1) Time-at-risk definition and potential left-truncation / selection bias
The Methods state Cox-PH models with age as the time scale and that “follow-up began at birth,” with follow-up ending at earliest diagnosis, death, last registry follow-up, or age 80. It is also noted that diagnoses are not limited to information after cohort enrollment. This raises a key interpretability issue: are these Cox models implemented with delayed entry at cohort entry age (left truncation), and are prevalent cases handled consistently? If events prior to cohort entry are included without careful treatment of delayed entry and prevalence, selection mechanisms (survival to enrollment, linkage availability) can bias hazard ratios and, importantly, can induce or distort apparent PGS * education interaction patterns.

Requested action: Please add an explicit schematic/model definition specifying: (a) entry time (age at cohort entry?) and whether delayed entry is used; (b) whether individuals with pre-entry diagnoses are excluded for each outcome; (c) how see-through “cases” are defined relative to entry; and (d) cohort-specific availability of outcome data. A strong sensitivity analysis would restrict to incident-only events after entry (with delayed entry), excluding prior diagnoses, to test whether key PGS×education conclusions persist.

2) Interpretation of “education” (SES) and robustness of the binary split
Education is dichotomized at ISCED ≥5 vs ≤4, with the cutoff chosen based on the FinnGen distribution to preserve sample size/case counts across all outcomes. This is pragmatic for power, but it may (i) mask nonlinear gradients, (ii) create cross-country comparability issues where ISCED categories may not map to equivalent social/economic meaning, and (iii) contribute to between-cohort heterogeneity.

Requested action: Please add sensitivity analyses using (a) 3–4 education categories (or ordinal ISCED) to assess

nonlinearity; and (b) cohort-specific splits (e.g., within-cohort quantiles or country-specific thresholds) to test robustness of interaction/stratified PGS effects. You already explore an alternative SES proxy (occupation) in FinnGen; I suggest elevating that as a key robustness check and being explicit that the "SES" conclusions are conditional on these operationalizations.

ONE MORE THING (genetics of education / interpretation of interaction):
Educational attainment is partly heritable and genetically correlated with many health-related traits. Therefore, treating education purely as an environmental "SES exposure" can complicate interpretation of the Disease-PGS * Education interaction as a gene–environment interaction. The observed effect modification could reflect gene–environment correlation and/or shared genetic architecture (pleiotropy), and (if modeled explicitly) could be closer to PGS×PGS (G×G) than a clean G×E contrast. I recommend clarifying the intended causal interpretation and adding a sensitivity analysis that accounts for the genetic component of education—for example, including an educational attainment PGS as a covariate and/or testing Disease-PGS × Edu-PGS alongside Disease-PGS × observed education. At minimum, reporting the association/correlation between Disease PGS and education (and discussing genetic nurture/dynastic effects as alternative explanations) would help readers interpret whether the apparent effect modification reflects environment/SES versus genetic confounding.

3) Main pattern "PGS effects larger among high education" may reflect differential ascertainment/detection
The manuscript reports that for several diseases, the PGS hazard ratio is larger among individuals with high education. While plausible, this pattern can also arise from differential diagnosis/ascertainment by education (e.g., higher health care utilization, screening, or differential coding), especially when outcomes are EHR/registry-based and data sources differ across cohorts.

Requested action: Please add at least one robustness check aimed at ascertainment. For example:
- For diseases sensitive to screening/detection (notably cancers), restrict to more severe endpoints (e.g., hospitalizations/procedures) to reduce detection bias.
- Expand the Discussion to explicitly consider differential detection/healthcare utilization as an alternative explanation for the direction of interaction.

4) Meta-analysis strategy: fixed-effects despite non-trivial heterogeneity
Fixed-effects meta-analysis is used, with heterogeneity assessed via Cochran's Q. Several outcomes show meaningful heterogeneity. When heterogeneity is present, fixed-effects estimates can be misleading as a "universal effect," and interpretation should be more cautious.

Requested action: Please provide random-effects meta-analysis estimates (or at minimum prediction intervals) as sensitivity analyses for the main claims, especially for outcomes with significant heterogeneity. If conclusions differ materially, that should be highlighted.

5) Prediction modeling is not fully aligned or consistent with the time-to-event framework
The prediction analysis evaluates discrimination and reclassification (AUC, continuous NRI, continuous IDI) using logistic regression models trained in 80% of FinnGen and tested in 20% (with validation in UK Biobank). However, the primary outcomes are time-to-event with censoring and varying follow-up, and the prediction target/horizon is not fully explicit.

Requested action: Please clarify the prediction target (e.g., "diagnosis by age 80," "ever diagnosed in registry," or another fixed horizon). Consider adding survival-appropriate prediction metrics (e.g., C-index, time-dependent AUC at pre-specified horizons, calibration plots/Brier score), or a sensitivity analysis demonstrating that the logistic approach yields consistent qualitative conclusions under a clearly defined time horizon.

6) Clarify proportional hazards assumption checks across cohorts
Proportional hazards checks are described (scaled Schoenfeld residuals) but appear to be reported for FinnGen specifically. It is unclear whether comparable checks were performed in other cohorts and/or for the outcomes that drive the main interaction claims.

Requested action: Please clarify whether PH assumptions were checked in UK Biobank and Generation Scotland (at least for the main outcomes with significant interactions). If not, provide reason and/or a limited set of additional checks.

MINOR COMMENTS

1) Please ensure consistent terminology for the meta-analysis approach: figure legends refer to "mixed-effects meta-analyzed" hazard ratios, while Methods describe fixed-effects. Is "mixed-effects" referring to a different model?

2) Non-European ancestry analyses in UK Biobank: please emphasize these results as exploratory given limited sample size and known portability limitations of PGS across ancestries, and avoid overgeneralizing.

STATISTICAL AND REPRODUCIBILITY SUMMARY
The overall statistical plan is reasonable (Cox PH with age as time scale, multiple outcomes, stratified/interaction models, multiple testing control). The main statistical concerns are (i) clear definition of time-at-risk and handling of diagnoses relative to cohort entry (left truncation/prevalent cases), (ii) interpretation of PGS×education interactions in the presence of potential ascertainment differences by education, and (iii) alignment of the prediction analysis with censoring/time-to-event outcomes. Addressing these points would substantially strengthen causal interpretation and the robustness of the key

conclusions.

Reviewer #2

(Remarks to the Author)
Summary:

This is an interesting and informative study that systematically evaluates the contrasting associations of socioeconomic status and genetic risk for prediction of disease incidence.

Firstly, and overwhelmingly, there are many strengths to this work: the study design involves implementing a complex statistical genetic workflow for polygenic score evaluation across multiple disease areas, with model training and validation across very large well-characterised cohorts with substantial follow-up (UKB, FinnGen, GS), using time-to-event analysis to assess prediction of disease incidence, with independent and interactive associations. The main manuscript figure designs are particularly strong in communicating the findings across multiple disease areas with precision and clarity, and the overall quality and conciseness of report is impressive. Existing model selection, sensitivity analyses and testing of assumptions within the manuscript (e.g., for competing risks, proportional hazards) are comprehensive.

For developing further – I would suggest the framing into causal language at times exceeds what the study design supports. This would mean reframing conclusions to be primarily (but not necessarily exclusively) predictive/associational. Current literature standards for distinguishing causal/non-causal interpretations are increasingly high. In my view, using causal speculations to interpret the results should be more limited and secondary to not distract from this.

Specific comments:

Causal overinterpretation

Title and Abstract
1. The title is potentially inconsistent in using both a causal term “impact” (because in plain English if I say A impacts B that naturally speaks to a causal connection) and the non-causal “predicting risk” (factors can predict risk without being causes of it). Page 2, Line 34, the phrase “contributions.. to disease risk” in the Abstract is speaking of causality, because if we are arguing that “factor X (education, genetics) contributes to risk of outcome Y (disease)” this is already stronger that e.g. “contributes to predictive performance or variance explained”, but the study design is the latter.

Results

2. Throughout the results we have both “education effects” and “prediction” e.g. the subtitle “Effects of PGSs, education, and their interaction on disease prediction” which mixes causal language “effects” with non-causal “prediction”.

Discussion

3. In the Discussion we have the primary conclusions immediately drawn in causal language: Page 7, 157 “education attainment, and PGSs both strongly affect the disease risk..”, with “affect” being a causal term. Likewise, Page 7, Line 157 “influence of SES on health outcomes.. our findings are consistent… demonstrating how lower SES contributes to susceptibility for premature mortality…”. Here, “influence” is causal, and “contributes to susceptibility” also would refer to SES lying on the causal pathway. These statements imply consistency of results with causal literature, though the literature cited is generally more of association tests and prediction. Only after a full account of possible causal explanations is given that the authors return to interpretations in terms of risk prediction/associations (Page 8, Line 172)

4. Given the interest in causal interpretations, it may be helpful to see a comment on how a true causal inference study design in future work would strengthen these conclusions, and how this might look in practice to perform, and whether the cohorts and variables here are sufficient to do the analysis.

Interaction of genetic score with education
5. For model 3 (and to some extent model 4) - which is Methods Page 13, Line 266 and Results Page 5, Line 95-105 - the authors consider the strength of PGS on risk of disease (incidence as time-to-event), compared across high/low education strata, which is presented in Figure 2. It is noted that the HR is often stronger for the high education group. The Discussion section says that “educational attainment, often associated with more favorable social and lifestyle factors, may facilitate a stronger expression of genetic predispositions”. Hence the authors are arguing these results could be interpreted as potentially a true biological/mechanistic effect modification. This is quite a striking comment – though is it worth elaborating on why this could plausibly be the case?

6. Related, it might be worth considering the difference between relative and absolute scales – primarily the results here use HRs which is a relative scale. For interaction, if for a given disease outcome the baseline hazard is lower for high educational strata, then the same absolute increase in genetic risk would lead to a larger HR being identified within this educational strata. Hence I think we would expect interactions to go in the direction of HRs being higher for high vs low education strata, as typically this is a lower disease risk group in absolute terms. Perhaps a comment on this needs to be made, as a further possible interpretation, or even additionally/alternatively ensuring and confirming that these current findings hold true on the absolute scale based on e.g. the cumulative risk plots.

Reviewer #3

(Remarks to the Author)
The authors present a study of the joint contribution of socioeconomic status (quantified as educational attainment) and genetic risk on disease status in mostly European individuals from three well-powered samples. The question is an interesting one and results suggest that associations between educational attainment and disease risk are mostly independent of genetics risk and that prediction models can be slightly improved for some disease outcomes if both are included. Overall the paper is well written and the results well presented. However, we have one general comment and a small number of more specific comments we would like to see addressed.

General comment:
Throughout the manuscript the authors refer to SES and educational attainment interchangeably. But we would argue that although SES and education are correlated both phenotypically and genetically these are not exactly the same, and in fact we feel this study is really examining the association between education, PGS and disease as opposed to SES. We feel that this conflation is a major limitation and as an overarching comment educational attainments use as a proxy for SES requires greater justification, particularly when other phenotypes are available. For example, in the UKB the Townsend Deprivation Index at baseline is available for all individuals. It would strike us that this would be more logical as the primary outcome if thinking about SES in the UK Biobank, as opposed to educational attainment. Further, occupation is used as a sensitivity analysis in FinnGen but not in the UKB. Why, when job codes are also recorded in the UKB?
We appreciate that the use of education as a proxy is discussed in the limitations, and agree that it would be of interest to evaluate different indices. However, this could actually be done for this study as other measures, as discussed above, are already available in the samples used here.
Overall, more justification for using educational attainment as the proxy for SES is required and if other measures of SES are available these should also be tested for comparison at minimum.

Specific comments
Introduction
1. The authors state that they set out to test three specific hypothesis, one of which is that genetic risk and SES are independently associated with disease risk. However, it is not clear what motivates this hypothesis, as opposed to say an interaction. Further, earlier in the introduction (line 42-54) they specifically state: “PGSs for educational attainment predict common diseases such as major depression, type 2 diabetes (T2D), and heart disease, and including the PGS for educational attainment, the disease-specific PGS, and their interaction substantially increases disease prediction”. Given there is an interaction between genetic risk for education and disease PGS, would we not expect an interaction between a disease PGS and an education phenotype? Some details as to how this hypothesis was arrived at, with reference to the literature, would be clarifying.

Methods
1. On line 228: Why these 19 diseases and not others? Was a burden or prevalence threshold decided upon prior to selection?
2. On line 234: Were cases allowed to belong to more than one disease category, as in if there were individuals with ICD codes for both alcohol use disorder and gout, were they allowed to be cases in analyses of both disease outcomes? If so we would suggest sensitivity analyses be performed with mutually exclusive cases, which may even strengthen associations.
3. On line 312: When the authors say they validated the findings in the UKB, do they mean they re-ran models in the same logistic regression models from FinnGen in the UKB or they use the models trained in the FinnGen 80% training sample to predict into the UKB?
4. The authors look at the association of PGS and education levels separately first (adjusting for each other in secondary models). Then then stratified the association of PGS on each disease by education level. Although the authors mention interaction it is not clear whether the results of the interaction between PGS and education informed stratification. We would have expected that authors test first for an interaction and then stratify on education based on these results- could the author explain the reasons behind their pipeline?
5. Why did cumulative incidence estimation analyses stratified by PGS and education level was done only for FinnGen?
6. Why were logistic models (as opposed to cox models) used for prediction?

Results and Discussion
1. In Figure 4 and paragraph beginning line 116: The difference between education and the other models appears to be much more spread out (at least for some disease endpoints) in the UKB than in FinnGen. For example, see for Type 1 Diabetes. Addityioanlly, combined models seems to perform better in FinnGen compared to PGS alone than in the UKB. What do the authors think drives these differences? Selection bias in the UKB? We would like to see this discussed at least briefly in the discussion section.

Reviewer #4

(Remarks to the Author)
I co-reviewed this manuscript with one of the reviewers who provided the listed reports. This is part of the Communications Medicine initiative to facilitate training in peer review and to provide appropriate recognition for Early Career Researchers who co-review manuscripts.

Version 1:

Reviewer comments:

Reviewer #1

(Remarks to the Author)
I thank the author for their careful and constructive revisions. I believe the revised manuscript is substantially improved and addresses my previous comments adequately. I have no further substantive concerns and consider the manuscript suitable for publication.

Reviewer #2

(Remarks to the Author)
I am fully satisfied with the revised manuscript: the additional sensitivity analyses, revised discussion, abstract and title, and improved interpretation of causation vs association have further strengthened this work.
I have no further comments, and I greatly appreciate the time and effort the authors have placed in responding to the comments.

Reviewer #3

(Remarks to the Author)
The authors have successfully addressed all our comments

Reviewer #4

(Remarks to the Author)
I co-reviewed this manuscript with one of the reviewers who provided the listed reports. This is part of the Communications Medicine initiative to facilitate training in peer review and to provide appropriate recognition for Early Career Researchers who co-review manuscripts.

Open Access This Peer Review File is licensed under a Creative Commons Attribution 4.0 International License, which permits use, sharing, adaptation, distribution and reproduction in any medium or format, as long as you give appropriate credit to the original author(s) and the source, provide a link to the Creative Commons license, and indicate if changes were made.
In cases where reviewers are anonymous, credit should be given to 'Anonymous Referee' and the source.
The images or other third party material in this Peer Review File are included in the article's Creative Commons license, unless indicated otherwise in a credit line to the material. If material is not included in the article's Creative Commons license and your intended use is not permitted by statutory regulation or exceeds the permitted use, you will need to obtain permission directly from the copyright holder.
To view a copy of this license, visit https://creativecommons.org/licenses/by/4.0/

Point-by-point Reviewer Responses COMMSMED-25-2663-T revisions 1

<u>Reviewer #1:</u>

Summary

This manuscript evaluates how disease-specific polygenic scores (PGSs) and socioeconomic status (SES), operationalized primarily as educational attainment (high vs low), relate to risk of 19 complex diseases across FinnGen, UK Biobank, and

Generation Scotland, with an additional non-European ancestry analysis in UK Biobank. The authors fit Cox proportional hazards models (age as the time scale) and meta-analyze effects across cohorts, testing independent contributions of PGS and education and also PGS×education interaction/stratified effects. They additionally assess whether adding education to PGS improves disease prediction in held-out data, reporting AUC and reclassification metrics.

Overall assessment

This is an interesting and well-powered cross-biobank analysis addressing a broadly relevant question: whether and how SES modifies genetic risk, and whether SES and genetic risk provide complementary information for prediction. Strengths include the multi-cohort design, standardized approach across 19 outcomes, explicit interaction/ stratified analyses, and multiple sensitivity checks. The main areas that would benefit from clarification/strengthening are (i) definition of time-at-risk and handling of diagnoses relative to cohort entry, (ii) interpretation and robustness of the education (SES) operationalization, (iii) potential ascertainment/detection differences by education that could mimic effect modification, and (iv) alignment of the prediction modeling with the time-to-event framework.

We are grateful to the reviewer for their careful reading of our manuscript and for their constructive and detailed feedback. We appreciate the recognition of the strengths of our study and the valuable and detailed suggestions for clarification and improvement. Below, we respond to each comment and describe the corresponding revisions made to the manuscript.

MAJOR COMMENTS

1) Time-at-risk definition and potential left-truncation / selection bias

The Methods state Cox-PH models with age as the time scale and that "follow-up began at birth," with follow-up ending at earliest diagnosis, death, last registry follow-up, or age 80. It is also noted that diagnoses are not limited to information after cohort enrollment. This raises a key interpretability issue: are these Cox models implemented with delayed entry at cohort entry age (left truncation), and are prevalent cases handled consistently? If events prior to cohort entry are included without careful treatment of delayed entry and prevalence, selection mechanisms (survival to enrollment, linkage availability) can bias hazard ratios and, importantly, can induce or distort apparent PGS * education interaction patterns.

Requested action: Please add an explicit schematic/model definition specifying: (a) entry time (age at cohort entry?) and whether delayed entry is used; (b) whether individuals with pre-entry diagnoses are excluded for each outcome; (c) how see-through "cases" are defined relative to entry; and (d) cohort-specific availability of

outcome data. A strong sensitivity analysis would restrict to incident-only events after entry (with delayed entry), excluding prior diagnoses, to test whether key PGS×education conclusions persist.

We thank the reviewer for this important comment regarding the definition of time-at-risk and the handling of diagnoses relative to cohort entry. We agree that careful specification of the time scale, entry conditions, and outcome ascertainment is essential for interpretation. In our primary analyses, we used age as the underlying time scale, with follow-up defined from birth until the earliest of disease diagnosis, death, end of registry follow-up, or age 80. To ensure more comparable exposure to educational attainment, we further restricted follow-up to the age range of 35–80 years. As noted by the reviewer, this approach does not explicitly implement delayed entry at cohort and thus includes events that occurred prior to cohort entry. Outcome ascertainment in FinnGen is based on nationwide health registries, in the UK Biobank from the hospital episode statistics, and in Generation Scotland from primary and secondary health records, with coverage depending on the specific data source. As such, the effective observation window varies by outcome, and we have moved the registry-specific start dates and data sources from the supplementary information to the **Methods** (pages 6-8, lines 117-164).

We acknowledge that inclusion of pre-entry events without delayed entry may introduce selection mechanisms related to survival to cohort enrolment and registry coverage, which could influence hazard ratio estimates and, in principle, interaction patterns. To address this concern, we have included a sensitivity analysis in FinnGen restricted to incident-only events. When comparing the original FinnGen results with those of the sensitivity analyses using two-sided Wald tests (Bonferroni-corrected for 19 outcomes), we observed no significant differences for the disease-specific PGS by educational attainment interactions (**Supplementary Fig. 16**, **Supplementary Data 37-39**). These sensitivity analyses have been added to the manuscript (see **Methods** page 19-20, lines 438-450; **Results** page 26, lines 608-613).

2) Interpretation of "education" (SES) and robustness of the binary split

Education is dichotomized at ISCED ≥5 vs ≤4, with the cutoff chosen based on the FinnGen distribution to preserve sample size/case counts across all outcomes. This is pragmatic for power, but it may (i) mask nonlinear gradients, (ii) create cross-country comparability issues where ISCED categories may not map to equivalent social/ economic meaning, and (iii) contribute to betweencohort heterogeneity.

Requested action: Please add sensitivity analyses using (a) 3–4 education categories (or ordinal ISCED) to assess nonlinearity; and (b) cohort-specific splits (e.g., within-cohort quantiles or country-specific thresholds) to test robustness of interaction/stratified PGS effects. You already explore an alternative SES proxy (occupation) in FinnGen; I suggest

elevating that as a key robustness check and being explicit that the "SES" conclusions are conditional on these operationalizations.

We have followed the reviewer's suggestions and have added two sensitivity analyses, one where we do not dichotomize education and one where we assess cohort-specific dichotomization (see **Methods**, pages 19, lines 420-435). When applying a cohort-specific threshold in the UK Biobank, we still observe significant interaction effects for all seven traits, but now also observe significant interaction for seven additional traits (**Supplementary Data 33**). However, the interaction associations for alcohol use disorder, rheumatoid arthritis, gout, type 1 diabetes, breast and prostate cancer differ significantly when compared with two-sided Wald tests with the results using the same threshold across all three cohorts (**Supplementary Fig. 14**, **Supplementary Data 34**). So, while our conclusion on the significance of the seven diseases showing interactions are robust, we do observe differences in both the significance and effect sizes of the interactions for other traits. Similarly, when we do not dichotomize educational attainment, we still see significant interactions for the seven traits (**Supplementary Data 35**), however, these interactions are significantly attenuated (**Supplementary Fig. 15**, **Supplementary Data 36**). These results have been included in the **Results** (page 26, lines 595-607) and briefly addressed in the **Discussion** (page 28, lines 656-660):

"In addition, although educational attainment was harmonized using ISCED categories, the dichotomization threshold was defined based on the FinnGen distribution and applied across cohorts, which may reduce sensitivity to socioeconomic variation in the UK Biobank. Consistent with this, applying cohort-specific thresholds resulted in differences in interaction estimates, although the overall conclusions remained robust."

We also thank the reviewer for the suggestion to highlight our occupation analyses in FinnGen as a key robustness check. Based on this suggestion and the suggestions of the editor and reviewers 3 and 4, we have chosen to also perform the occupation analyses in the UK Biobank and Generation Scotland and now present the meta-analysed estimates in the manuscript (see **Methods** pages 8-10, lines 171-217; **Methods** page 15-16, lines 338-367; and **Results** pages 24-25, lines 559-586). In the **Discussion** (page 31, lines 726-728), we reiterate that our conclusions for socioeconomic status are conditional on our operationalizations for educational attainment and occupation.

ONE MORE THING (genetics of education / interpretation of interaction):

Educational attainment is partly heritable and genetically correlated with many health-related traits. Therefore, treating education purely as an environmental "SES exposure" can complicate interpretation of the Disease-PGS * Education interaction as a gene–environment interaction. The observed effect modification could reflect gene–

environment correlation and/or shared genetic architecture (pleiotropy), and (if modeled explicitly) could be closer to PGS×PGS (G×G) than a clean G×E contrast. I recommend clarifying the intended causal interpretation and adding a sensitivity analysis that accounts for the genetic component of education—for example, including an educational attainment PGS as a covariate and/or testing Disease-PGS × Edu-PGS alongside Disease-PGS × observed education. At minimum, reporting the association/correlation between Disease PGS and education (and discussing genetic nurture/dynastic effects as alternative explanations) would help readers interpret whether the apparent effect modification reflects environment/SES versus genetic confounding.

We agree with the reviewer that educational attainment itself is a heritable trait showing genetic correlations with many complex diseases and health-related traits and can therefore not been seen as a purely environmental exposure. The modification could indeed more closely resemble a GxG than a GxE effect. We have followed the reviewer's suggestion and have added a sensitivity analysis in FinnGen where we add a PGS for educational attainment (https://doi.org/10.1038/s41588-018-0147-3) as well as the interaction between the disease-specific PGS and the EA PGS (see **Methods** pages 20-21, lines 460-470; **Results** page 27, lines 618-621). In these models the interaction associations of the disease-specific PGS by educational attainment remain significant for each of the seven traits (**Supplementary Data 43**) and these are not significantly attenuated (**Supplementary Fig. 18**, **Supplementary Data 44**). Moreover, the EA PGS itself is significantly associated with 5 out of the 7 diseases for which we observed a significant disease-specific PGS by educational attainment interaction (atrial fibrillation, asthma, coronary heart disease, and hip and knee osteoarthritis) as well as with gout, kin melanoma, lung cancer, breast cancer, and alcohol use disorder (**Supplementary Data 43**). The association directions of the EA PGS are in line with those observed for educational attainment, though effect sizes are smaller (HR range = 0.91-1.08, 95% CI range = 0.90-1.12). In contrast to the disease-specific PGS by educational attainment interaction, we observed no significant EA PGS by disease-specific PGS interactions (**Supplementary Data 43**). In the **Discussion** (Pages 29-30, lines 686-698), we now address the results of this sensitivity analysis in terms of potential genetic confounding where we also discuss that genetic nurture or dynastic effects might still play a role:

"Interpretation of these findings is further complicated by the fact that educational attainment itself is a genetically influenced trait[14,59,82]. As such, we cannot fully exclude genetic confounding in the observed PGS by education interactions. Although these interactions were robust to additional adjustment for an educational attainment PGS and its interaction with the disease-specific PGS, this approach does not fully disentangle environmental from genetic sources of effect modification. Educational attainment PGSs capture not only direct genetic influences, but also gene-

environment correlations, whereby individuals with higher PGSs are more likely to experience favorable social and environmental conditions[83]. In addition, parental genotypes can shape offspring environments through genetic nurture (or dynastic effects), whereby parental alleles influence offspring outcomes via environmental pathways independent of direct genetic transmission[84,85]. Together, these processes suggest that the observed effect modification by education may reflect a combination of environmental stratification and shared genetic architecture, rather than a purely environmental interaction, and should therefore be interpreted with appropriate caution."

3) Main pattern "PGS effects larger among high education" may reflect differential ascertainment/detection

The manuscript reports that for several diseases, the PGS hazard ratio is larger among individuals with high education. While plausible, this pattern can also arise from differential diagnosis/ascertainment by education (e.g., higher health care utilization, screening, or differential coding), especially when outcomes are EHR/registry-based and data sources differ across cohorts.

Requested action: Please add at least one robustness check aimed at ascertainment. For example:

- For diseases sensitive to screening/detection (notably cancers), restrict to more severe endpoints (e.g., hospitalizations/procedures) to reduce detection bias.

- Expand the Discussion to explicitly consider differential detection/healthcare utilization as an alternative explanation for the direction of interaction.

We agree that differential diagnosis, healthcare utilization, and ascertainment across educational strata may contribute to the observed differences in hazard ratios and should be considered as an alternative explanation for the observed disease-specific PGS by educational attainment interaction patterns. In our **Discussion**, we had already acknowledged screening differences as a possible explanation for the associations between SES and most common cancers (page 28, lines 641-645), and differences in e.g., healthcare utilization as one of the possible explanations for the observed interactions (pages 28-29, lines 662-669). On the reviewer's recommendation we have expanded the **Discussion** and acknowledge that differential detection by education may contribute to the direction and magnitude of the observed interactions (page 29, lines 669-677):

"At the same time, these differences in healthcare utilization may also influence disease detection. Individuals with higher SES are more likely to participate in screening programs and undergo more frequent or earlier diagnostic evaluations, which may increase the likelihood of disease detection independent of underlying biological risk[79–

[81]. As a result, some of the observed differences in hazard across educational strata may partly reflect differential ascertainment rather than true effect modification, particularly for conditions with substantial diagnostic discretion. While many of the outcomes in our study are primarily based on hospital and registry data, which may reduce such biases, differential detection cannot be fully excluded and should be considered when interpreting these findings."

4) Meta-analysis strategy: fixed-effects despite non-trivial heterogeneity

Fixed-effects meta-analysis is used, with heterogeneity assessed via Cochran's Q. Several outcomes show meaningful heterogeneity. When heterogeneity is present, fixed-effects estimates can be misleading as a "universal effect," and interpretation should be more cautious.

Requested action: Please provide random-effects meta-analysis estimates (or at minimum prediction intervals) as sensitivity analyses for the main claims, especially for outcomes with significant heterogeneity. If conclusions differ materially, that should be highlighted.

We thank the reviewer for their suggestion to include random-effects meta-analytical effects given the observed heterogeneity for several of our outcomes. We have now repeated all meta-analyses in our manuscript with random-effects and include these results in the relevant supplementary figures and data files. However, since we only meta-analyse across three studies, we have chosen to refer to the fixed-effect meta-analytical results in the main text. Moreover, the random- and fixed-effects estimates have overlapping confidence intervals, consequently, our conclusion do not differ materially. Finally, as we have now added meta-analytical estimates for the occupation analyses, we have also provided the fixed- and random-effect estimates to the supplementary tables for the occupation analyses.

5) Prediction modeling is not fully aligned or consistent with the time-to-event framework

The prediction analysis evaluates discrimination and reclassification (AUC, continuous NRI, continuous IDI) using logistic regression models trained in 80% of FinnGen and tested in 20% (with validation in UK Biobank). However, the primary outcomes are time-to-event with censoring and varying follow-up, and the prediction target/horizon is not fully explicit.

Requested action: Please clarify the prediction target (e.g., "diagnosis by age 80," "ever diagnosed in registry," or another fixed horizon). Consider adding survival-appropriate prediction metrics (e.g., C-index, time-dependent AUC at pre-specified horizons, calibration plots/Brier score), or a sensitivity analysis demonstrating that the logistic

approach yields consistent qualitative conclusions under a clearly defined time horizon.

We thank the reviewer for pointing out that our prediction target was unclear and highlight the difficulty in interpreting association models with time-to-event and prediction models with logistic regression. In our **Methods** section (page 17, lines 389-390), we have now clarified that our prediction target was case status by the end of follow-up. Moreover, to streamline across the association and prediction analyses, we have repeated the training in 80% of the FinnGen participants with Cox proportional hazard models. Thus, the discrimination and reclassification results in the remaining 20% of the FinnGen participants and UK Biobank participants now also reflect time-to-event (**Results**, page 24, lines 540-558). Please note that the prediction results based on Cox-PH models are very similar to those we previously reported based on logistic regression models.

6) Clarify proportional hazards assumption checks across cohorts

Proportional hazards checks are described (scaled Schoenfeld residuals) but appear to be reported for FinnGen specifically. It is unclear whether comparable checks were performed in other cohorts and/or for the outcomes that drive the main interaction claims.

Requested action: Please clarify whether PH assumptions were checked in UK Biobank and Generation Scotland (at least for the main outcomes with significant interactions). If not, provide reason and/or a limited set of additional checks.

We apologize for not including the PH assumption checks for the UK Biobank and Generation Scotland. **Supplementary Figs 1-2** have been updated to now also include those cohorts.

MINOR COMMENTS

1) Please ensure consistent terminology for the meta-analysis approach: figure legends refer to "mixed-effects meta-analyzed" hazard ratios, while Methods describe fixed-effects. Is "mixed-effects" referring to a different model?
   We thank the reviewer for catching these typos, the legends should indeed have read "fixed-effect" and not "mixed-effects" and have now been adjusted.
2) Non-European ancestry analyses in UK Biobank: please emphasize these results as exploratory given limited sample size and known portability limitations of PGS across ancestries, and avoid overgeneralizing.
   We thank the reviewer for their suggestion. We have adapted the relevant limitation section in our **Discussion** (Pages 30-31, lines 712-715) as follows: "However, while our results in non-European ancestry individuals in the UKB were mostly in line with our observations in European ancestry individuals,

further studies are needed to ensure the portability of PGSs developed in European ancestry individuals to non-European ancestries[91,92]".

STATISTICAL AND REPRODUCIBILITY SUMMARY

The overall statistical plan is reasonable (Cox PH with age as time scale, multiple outcomes, stratified/interaction models, multiple testing control). The main statistical concerns are (i) clear definition of time-at-risk and handling of diagnoses relative to cohort entry (left truncation/prevalent cases), (ii) interpretation of PGS×education interactions in the presence of potential ascertainment differences by education, and (iii) alignment of the prediction analysis with censoring/time-to-event outcomes. Addressing these points would substantially strengthen causal interpretation and the robustness of the key conclusions.

We are grateful to the reviewer for their thorough assessment of the strengths and weaknesses of our manuscript and for their detailed recommendations on how we may approve the manuscript. By following the reviewer's recommendations we hope to have sufficiently addressed these key concerns.

Reviewer #2

Summary:

This is an interesting and informative study that systematically evaluates the contrasting associations of socioeconomic status and genetic risk for prediction of disease incidence.

Firstly, and overwhelmingly, there are many strengths to this work: the study design involves implementing a complex statistical genetic workflow for polygenic score evaluation across multiple disease areas, with model training and validation across very large well-characterised cohorts with substantial follow-up (UKB, FinnGen, GS), using time-to-event analysis to assess prediction of disease incidence, with independent and interactive associations. The main manuscript figure designs are particularly strong in communicating the findings across multiple disease areas with precision and clarity, and the overall quality and conciseness of report is impressive. Existing model selection, sensitivity analyses and testing of assumptions within the manuscript (e.g., for competing risks, proportional hazards) are comprehensive.

For developing further – I would suggest the framing into causal language at times exceeds what the study design supports. This would mean reframing conclusions to be primarily (but not necessarily exclusively) predictive/associational. Current literature standards for distinguishing causal/non-causal interpretations are increasingly high. In my view, using causal speculations to interpret the results should be more limited and secondary to not distract from this.

We thank the reviewer for their thorough and highly positive assessment of our study, and for their thoughtful and constructive feedback. We particularly appreciate the recognition of the strengths of our analytical framework and presentation. We also thank the reviewer for highlighting the importance of carefully distinguishing between associational and causal interpretations. In response, we have revised the manuscript to further clarify the interpretational scope and to avoid causal overstatement. Detailed responses to each point are provided below.

Specific comments:

Causal overinterpretation

Title and Abstract

1. The title is potentially inconsistent in using both a causal term "impact" (because in plain English if I say A impacts B that naturally speaks to a causal connection) and the non-causal "predicting risk" (factors can predict risk without being causes of it). Page 2, Line 34, the phrase "contributions... to disease risk" in the Abstract is speaking of causality, because if we are arguing that "factor X (education, genetics) contributes to risk of outcome Y (disease)" this is already stronger that e.g. "contributes to predictive performance or variance explained", but the study design is the latter.

We agree that our title unintentionally used causal language, we have now changed our title from "The impact of polygenic score and socioeconomic status in predicting risk for 19 complex diseases" to "Association and risk prediction of 19 complex diseases with polygenic scores and socioeconomic status".

To confirm with journal policies, we have now rewritten our abstract and in doing so have taken the reviewer's concern about causal language to heart. Consequently, former line 34 in the **Abstract** (now page 2, lines 44-45) now reads as follows: "Genetic variation and social circumstances are independently and partly jointly linked to differences in disease risk".

Results

2. Throughout the results we have both "education effects" and "prediction" e.g. the subtitle "Effects of PGSs, education, and their interaction on disease prediction" which mixes causal language "effects" with non-causal "prediction".

We thank the reviewer for bringing these inconsistencies to light. We have ensured that we use the word "association" whenever we discuss results from Cox proportional hazard models and have similarly adjusted the heading "Effects of PGSs, education, and their interaction on disease prediction" (**Results**, page 24, line 540) to "Predictive value of PGSs, education and their interaction on disease risk".

Discussion

3. In the Discussion we have the primary conclusions immediately drawn in causal language: Page 7, 157 "education attainment, and PGSs both strongly affect the disease risk..", with "affect" being a causal term. Likewise, Page 7, Line 157 "influence of SES on health outcomes.. our findings are consistent…demonstrating how lower SES contributes to susceptibility for premature mortality…". Here, "influence" is causal, and "contributes to susceptibility" also would refer to SES lying on the causal pathway. These statements imply consistency of results with causal literature, though the literature cited is generally more of association tests and prediction. Only after a full account of possible causal explanations is given that the authors return to interpretations in terms of risk prediction/associations (Page 8, Line 172)

We are grateful to the reviewer for highlighting several points in our discussion where we discuss our results with causal language when we did not intent to provide causal explanations for our results. We have addressed the use of causal language throughout the discussion. For example, Page 7 lines 157-160 "The influence of SES on health outcomes has been extensively studied, and our findings are consistent with previous work demonstrating how lower SES contributes to susceptibility for premature mortality[20] and chronic diseases like cardiovascular disease[21,22]." now reads "This is consistent with previous work linking lower SES to increased risk of premature mortality[61] and chronic diseases such as cardiovascular disease[62,63] [...]." (**Discussion**, page 28, lines 641-645).

4. Given the interest in causal interpretations, it may be helpful to see a comment on how a true causal inference study design in future work would strengthen these conclusions, and how this might look in practice to perform, and whether the cohorts and variables here are sufficient to do the analysis.

In the **Discussion** (page 30 lines 6990-706), where we originally ended with only a note on of caution for causal interpretations, we have now added suggestions on study designs that may strengthen causal inferences in future studies:

"Future work aimed at strengthening causal inference could leverage genetically informed family-based designs, such as within-family models, which can help separate direct genetic effects from genetic nurture and shared environmental influences[86]. Extensions incorporating quasi-experimental variation in educational exposure[87], for example policy reforms, or longitudinal designs with time-varying SES measures[88] may further clarify the extent to which SES modifies genetic risk through environmental pathways. While SES contributes to identification of high-risk individuals, its causal role remains uncertain and should be carefully considered when translating these findings into clinical or public health applications."

The cohorts and variables available in our study provide large-scale observational data which is well suited for detecting associations and effect

heterogeneity. However, additional data structures, such as genotyped family members on a large scale, longitudinal SES data, or natural experiments, would be required to more definitively distinguish causal mechanisms. Consequently, these cohorts are less suited to such analyses.

Interaction of genetic score with education

5. For model 3 (and to some extent model 4) - which is Methods Page 13, Line 266 and Results Page 5, Line 95-105 - the authors consider the strength of PGS on risk of disease (incidence as time-to-event), compared across high/low education strata, which is presented in Figure 2. It is noted that the HR is often stronger for the high education group. The Discussion section says that "educational attainment, often associated with more favorable social and lifestyle factors, may facilitate a stronger expression of genetic predispositions". Hence the authors are arguing these results could be interpreted as potentially a true biological/mechanistic effect modification. This is quite a striking comment – though is it worth elaborating on why this could plausibly be the case?

Please note that the sentence in the Discussion that read "Specifically, in the context of our results, high educational attainment, often associated with more favorable social and lifestyle factors, may facilitate a stronger expression of genetic predispositions[29]" has been removed from the current version of the manuscript.

6. Related, it might be worth considering the difference between relative and absolute scales – primarily the results here use HRs which is a relative scale. For interaction, if for a given disease outcome the baseline hazard is lower for high educational strata, then the same absolute increase in genetic risk would lead to a larger HR being identified within this educational strata. Hence I think we would expect interactions to go in the direction of HRs being higher for high vs low education strata, as typically this is a lower disease risk group in absolute terms. Perhaps a comment on this needs to be made, as a further possible interpretation, or even additionally/alternatively ensuring and confirming that these current findings hold true on the absolute scale based on e.g. the cumulative risk plots.

We thank the reviewer for their suggestion. In our **Discussion** (Page 29, lines 678-685), we have now included a note on our primary use of relative estimates and explicitly mention the cumulative risk estimates:

"Importantly, our primary analyses are conducted on the relative scale (hazard ratios), and differences in baseline risk across educational strata may contribute to the observed interaction patterns. For diseases with lower baseline risk among individuals with higher education, a similar absolute increase in risk associated with genetic predisposition may translate into a larger relative effect estimate. This could partly explain why stronger PGS associations are observed in higher-education groups.

However, cumulative incidence analyses showed similar qualitative patterns across educational strata, suggesting that the observed interactions are not solely driven by scaling differences, although effects on the absolute scale were generally smaller."

Reviewer #3 & #4

The authors present a study of the joint contribution of socioeconomic status (quantified as educational attainment) and genetic risk on disease status in mostly European individuals from three well-powered samples. The question is an interesting one and results suggest that associations between educational attainment and disease risk are mostly independent of genetics risk and that prediction models can be slightly improved for some disease outcomes if both are included. Overall the paper is well written and the results well presented. However, we have one general comment and a small number of more specific comments we would like to see addressed.

We sincerely thank the reviewers for their positive and constructive assessment of our work. We appreciate the recognition of the clarity of the manuscript and the importance of the research question. We have carefully addressed the general and specific comments and provide detailed responses below.

General comment:

Throughout the manuscript the authors refer to SES and educational attainment interchangeably. But we would argue that although SES and education are correlated both phenotypically and genetically these are not exactly the same, and in fact we feel this study is really examining the association between education, PGS and disease as opposed to SES. We feel that this conflation is a major limitation and as an overarching comment educational attainments use as a proxy for SES requires greater justification, particularly when other phenotypes are available. For example, in the UKB the Townsend Deprivation Index at baseline is available for all individuals. It would strike us that this would be more logical as the primary outcome if thinking about SES in the UK Biobank, as opposed to educational attainment. Further, occupation is used as a sensitivity analysis in FinnGen but not in the UKB. Why, when job codes are also recorded in the UKB?

We appreciate that the use of education as a proxy is discussed in the limitations, and agree that it would be of interest to evaluate different indices. However, this could actually be done for this study as other measures, as discussed above, are already available in the samples used here. Overall, more justification for using educational attainment as the proxy for SES is required and if other measures of SES are available these should also be tested for comparison at minimum.

In our original manuscript we primarily used educational attainment as proxy as this is available in all three cohorts and was standardizable across countries by converting educational attainment to ISCED 1997. Similarly, while job codes are available in UK Biobank and Generation Scotland, we initially only analysed occupation as an alternative SES measure as no standardized harmonization measure is available for occupation. However, we concur with the reviewers (and editor) that a paper focused on SES should not solely comprise of educational attainment. In the current version of the manuscript we have attempted to harmonize job codes across FinnGen, the UK Biobank, and Generation Scotland to the best of our abilities. Consequently, we now report meta-analytical results across all three cohorts for occupation as well (see **Methods** pages 8-10, lines 171-217; **Methods** page 15-16, lines 338-367; and **Results** pages 24-25, lines 559-586). Finally, while it would have been of great interest to expand the SES indices to e.g., the Townsend Deprivation index or income, we were restricted in which SES measures we are allowed to investigate in conjunction with genetic data in FinnGen. Therefore, we were unable to expand our SES measures further.

Specific comments

Introduction

1. The authors state that they set out to test three specific hypothesis, one of which is that genetic risk and SES are independently associated with disease risk. However, it is not clear what motivates this hypothesis, as opposed to say an interaction. Further, earlier in the introduction (line 42-54) they specifically state: " PGSs for educational attainment predict common diseases such as major depression, type 2 diabetes (T2D), and heart disease, and including the PGS for educational attainment, the disease-specific PGS, and their interaction substantially increases disease prediction". Given there is an interaction between genetic risk for education and disease PGS, would we not expect an interaction between a disease PGS and an education phenotype? Some details as to how this hypothesis was arrived at, with reference to the literature, would be clarifying.

We concur with the reviewer that given the known interactions between a PGS for educational attainment and disease-specific PGSs, we would indeed expect interactions between diseases-specific PGSs and observed educational attainment. Hence, we have phrased this expectation as our second hypothesis. Moreover, as noted in the **Methods** (page 16, lines 354-356) "differences in the strength or direction of association can occur even if the main effects are (largely) independent". Thus, even if we expect to find interaction, the main effects for the disease-specific PGS and SES measures could still be independent, therefore, we included this as our first hypothesis.

Methods

1. On line 228: Why these 19 diseases and not others? Was a burden or prevalence threshold decided upon prior to selection?

The selection of 18 out of 19 diseases (i.e., all diseases except alcohol use disorder, the reasoning for the inclusion of AUD is provided in the **Methods** page 6 lines 108-111) was made in a prior publication (https://doi.org/10.1038/s41467-024-48938-2). There, we chose to include these 18 traits because they had GWAS summary statistics available for the calculation of PGSs and their global burden (based on their disability-adjusted life years [DALYs] from the 2019 Global Burden of Disease). Together these 18 diseases accounted for 17.8% of the total global DALYs and 25% of the total DALY's in high-income countries.

2. On line 234: Were cases allowed to belong to more than one disease category, as in if there were individuals with ICD codes for both alcohol use disorder and gout, were they allowed to be cases in analyses of both disease outcomes? If so we would suggest sensitivity analyses be performed with mutually exclusive cases, which may even strengthen associations.

In our primary analyses we indeed allowed individuals to be cases for more than one of the studied complex diseases. As recommended, we have added sensitivity analyses for our interaction analyses in FinnGen where we removed individuals who were a case for more than one of the studied outcomes. Please note, that we made an exception for individuals with an "any cancer" diagnosis. We removed cases with a non-cancer diagnosis from the "any cancer" cases (and vice versa) and cases with one of the specific cancers we investigate (skin melanoma, prostate, breast, colorectal, and lung cancer) from the "any cancer" cases. However, we did not remove individuals with an "any cancer" diagnosis from the specific cancer group (otherwise no cases would remain as all cases would be removed, see **Methods**, page 20, lines 451-459; **Results** pages 26-27, lines 613-618). Out of the seven diseases for which we observed a significant disease-specific PGS by educational attainment interaction, only the interaction for T2D remains significant when restricting FinnGen analyses to mutually exclusive cases (**Supplementary Data 41**). While we observe attenuation of the interaction effect sizes (and vastly increased 95% confidence intervals) when restricting to mutually exclusive cases only, two-sided Wald tests (Bonferroni-corrected for 19 outcomes) indicate these estimates do not significantly differ from the unrestricted results (**Supplementary Fig. 17, Supplementary Data 42**). In contrast to the reviewers' expectations we do not observe a strengthening of associations, likely the result of the reduction in sample sizes when removing mutual cases.

3. On line 312: When the authors say they validated the findings in the UKB, do they mean they reran models in the same logistic regression models from FinnGen in the UKB or they use the models trained in the FinnGen 80% training sample to predict into the UKB?

We thank the reviewers for pointing out that the prediction pipeline was unclear. We trained models (now Cox proportional hazard models instead of logistic regression models) in 80% of the FinnGen sample, these models were then used to predict into the remaining 20% of the FinnGen sample and the UK Biobank. We have clarified this in the **Methods** (pages 17-18, lines 3890-393).

4. The authors look at the association of PGS and education levels separately first (adjusting for each other in secondary models). Then then stratified the association of PGS on each disease by education level. Although the authors mention interaction it is not clear whether the results of the interaction between PGS and education informed stratification. We would have expected that authors test first for an interaction and then stratify on education based on these results- could the author explain the reasons behind their pipeline?

Our analytical approach combines both interaction modelling and stratified analyses, which serve complementary purposes. Specifically, we formally tested for effect modification by including a disease-specific PGS by educational attainment interaction term in the regression models. The stratified analyses were conducted independently of the statistical significance of the interaction terms and were intended to facilitate interpretation and visualization of effect heterogeneity across educational strata. Stratified estimates and interaction terms reflect the same underlying model parameterization on the multiplicative (hazard ratio) scale. Thus, the interaction term provides a formal statistical test of effect modification, whereas stratified analyses allow for a more intuitive comparison of effect sizes across groups.

5. Why did cumulative incidence estimation analyses stratified by PGS and education level was done only for FinnGen?

As we only performed the cumulative incidence estimations for those diseases for which we observed a significant disease-specific PGS by educational attainment interaction, we were unable to perform these analyses in the UK Biobank, as the disease-specific PGSs for these seven traits included the UK Biobank in the GWAS summary statistics (also see **Methods** page 15, lines 341-343). Moreover, while we did look into the possibility of generating cumulative incidence estimates stratified by PGS and education (or occupation) in Generation Scotland, the stratified sample sizes were too small, resulting in nearly indistinguishable estimates and extremely wide bootstrapped standard errors. Consequently, we have chosen not to include cumulative incidence estimates for Generation Scotland.

6. Why were logistic models (as opposed to cox models) used for prediction?

Based on advice from reviewer 1 (Major comment #5), we have now replaced the logistic regression models with Cox proportional hazard models in training the prediction models.

Results and Discussion

1. In Figure 4 and paragraph beginning line 116: The difference between education and the other models appears to be much more spread out (at least for some disease endpoints) in the UKB than in FinnGen. For example, see for Type 1 Diabetes. Additionally, combined models seems to perform better in FinnGen compared to PGS alone than in the UKB. What do the authors think drives these differences? Selection bias in the UKB? We would like to see this discussed at least briefly in the discussion section.

We agree that the relative contribution of education and the combined models appears more variable in the UK Biobank compared to FinnGen for several outcomes, including Type 1 Diabetes.

We have now expanded the Discussion to address potential sources of these differences. First, differences in cohort characteristics and selection mechanisms likely contribute. The UK Biobank is known to exhibit a "healthy volunteer" selection profile, with participants generally healthier and of higher socioeconomic status than the underlying population, which may attenuate or alter associations involving socioeconomic indicators. In contrast, FinnGen is based on registry-linked population data, which may better capture the full socioeconomic gradient and associated disease burden.

Second, differences in outcome ascertainment and healthcare system structure between cohorts may also play a role. While both studies rely on registry-based data, variation in data sources, follow-up periods, and diagnostic practices can influence both baseline risk and predictive performance.

Third, as noted by reviewer 1 (Major comment #3), the operationalization of educational attainment may contribute to these differences. Although we harmonized education using ISCED categories, the dichotomization threshold was defined based on the FinnGen distribution and applied across cohorts. This may reduce sensitivity to socioeconomic variation in the UK Biobank. Indeed, in additional analyses using cohort-specific thresholds, we observe changes in both the magnitude and statistical significance of several interaction effects, although the main conclusions for the seven primary interaction findings remain robust (**Supplementary Fig. 14**, **Supplementary Data 34**).

Finally, differences in baseline disease prevalence and absolute risk between cohorts may also influence AUC estimates and the relative improvement from adding predictors, which can contribute to the observed variation in model performance.

This reasoning has been summarized in the **Discussion** (pages 28, lines 652-660): "The magnitude of predictive improvement and the relative contribution of

education varied across cohorts, with somewhat greater gains observed in FinnGen compared to the UK Biobank. These differences may reflect variation in cohort composition, including the "healthy volunteer" selection profile of the UK Biobank[70], which may attenuate socioeconomic gradients, as well as differences in outcome ascertainment and baseline disease risk. In addition, although educational attainment was harmonized using ISCED categories, the dichotomization threshold was defined based on the FinnGen distribution and applied across cohorts, which may reduce sensitivity to socioeconomic variation in the UK Biobank. Consistent with this, applying cohort-specific thresholds resulted in differences in interaction estimates, although the overall conclusions remained robust."
