# Supplementary material for: Association and risk prediction of 19 complex diseases with polygenic scores and socioeconomic status
Source: Commun Med (Lond). 2026 Jun 11;6:503. doi: 10.1038/s43856-026-01716-1 (PMC13616955; doi:10.1038/s43856-026-01716-1)

Supplementary Figures

Supplementary Fig. 1. Scaled Schoenfeld residuals of Cox proportional hazard models including the disease-specific polygenic score and educational attainment. **A.** Scaled Schoenfeld residuals in FinnGen study. **B.** Scaled Schoenfeld residuals in the UK Biobank. **C.** Scaled Schoenfeld residuals in Generation Scotland. Note that scaled Schoenfeld residuals could not be generated for Type 1 Diabetes, knee osteoarthritis, and lung cancer due to singularity problems.

A.

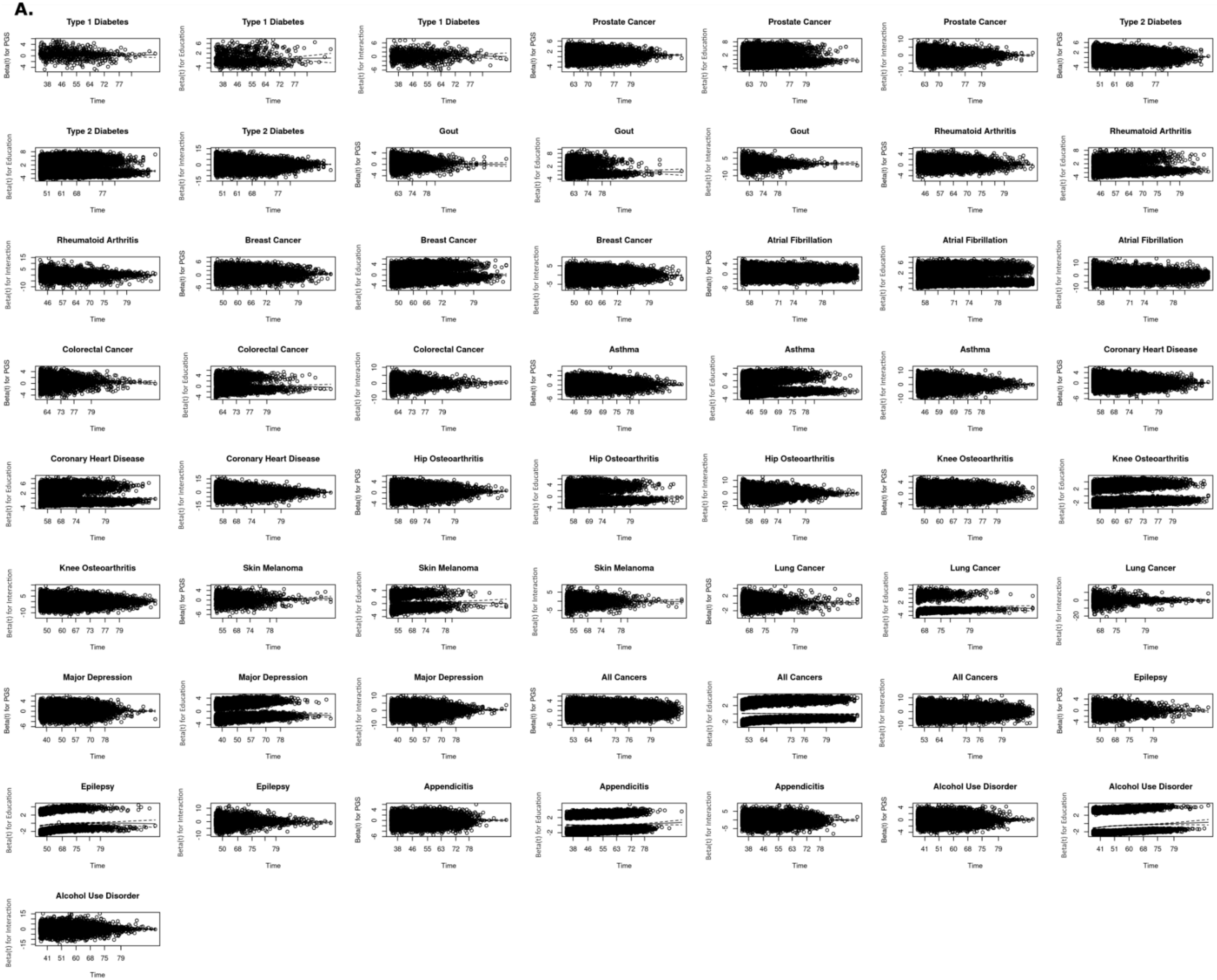

Type 1 Diabetes
Type 1 Diabetes
Type 1 Diabetes
Prostate Cancer
Prostate Cancer
Prostate Cancer
Type 2 Diabetes
Type 2 Diabetes
Type 2 Diabetes
Gout
Gout
Gout
Rheumatoid Arthritis
Rheumatoid Arthritis
Rheumatoid Arthritis
Breast Cancer
Breast Cancer
Breast Cancer
Atrial Fibrillation
Atrial Fibrillation
Atrial Fibrillation
Colorectal Cancer
Colorectal Cancer
Colorectal Cancer
Asthma
Asthma
Asthma
Coronary Heart Disease
Coronary Heart Disease
Coronary Heart Disease
Hip Osteoarthritis
Hip Osteoarthritis
Hip Osteoarthritis
Knee Osteoarthritis
Knee Osteoarthritis
Knee Osteoarthritis
Skin Melanoma
Skin Melanoma
Skin Melanoma
Lung Cancer
Lung Cancer
Lung Cancer
Major Depression
Major Depression
Major Depression
All Cancers
All Cancers
All Cancers
Epilepsy
Epilepsy
Epilepsy
Appendicitis
Appendicitis
Appendicitis
Alcohol Use Disorder
Alcohol Use Disorder
Alcohol Use Disorder
Beta(t) for PGS
Beta(t) for Education
Beta(t) for Interaction
Time

7

B.

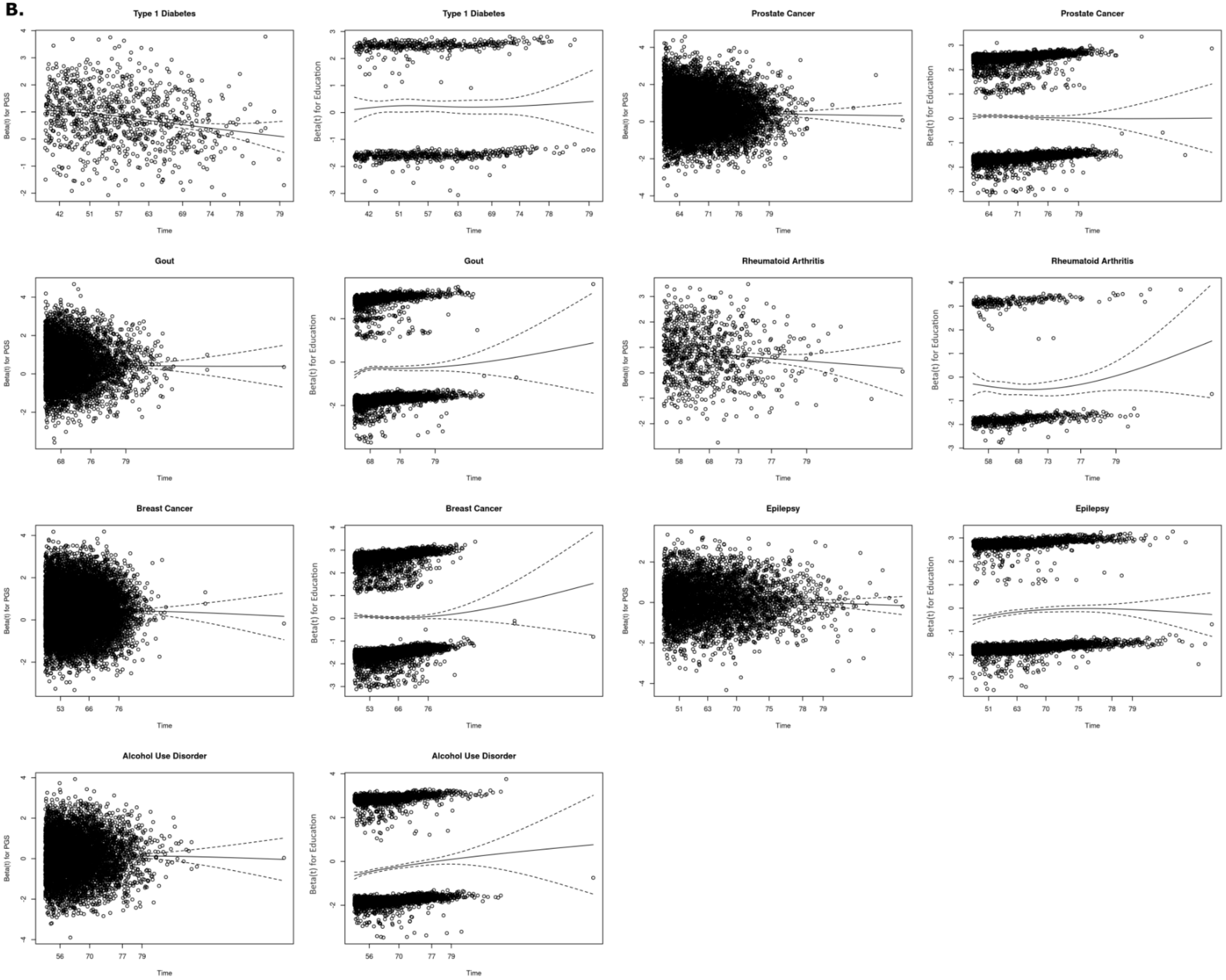

Type 1 Diabetes
Beta(t) for PGS
Time
Type 1 Diabetes
Beta(t) for Education
Time
Prostate Cancer
Beta(t) for PGS
Time
Prostate Cancer
Beta(t) for Education
Time
Gout
Beta(t) for PGS
Time
Gout
Beta(t) for Education
Time
Rheumatoid Arthritis
Beta(t) for PGS
Time
Rheumatoid Arthritis
Beta(t) for Education
Time
Breast Cancer
Beta(t) for PGS
Time
Breast Cancer
Beta(t) for Education
Time
Epilepsy
Beta(t) for PGS
Time
Epilepsy
Beta(t) for Education
Time
Alcohol Use Disorder
Beta(t) for PGS
Time
Alcohol Use Disorder
Beta(t) for Education
Time

8

c.

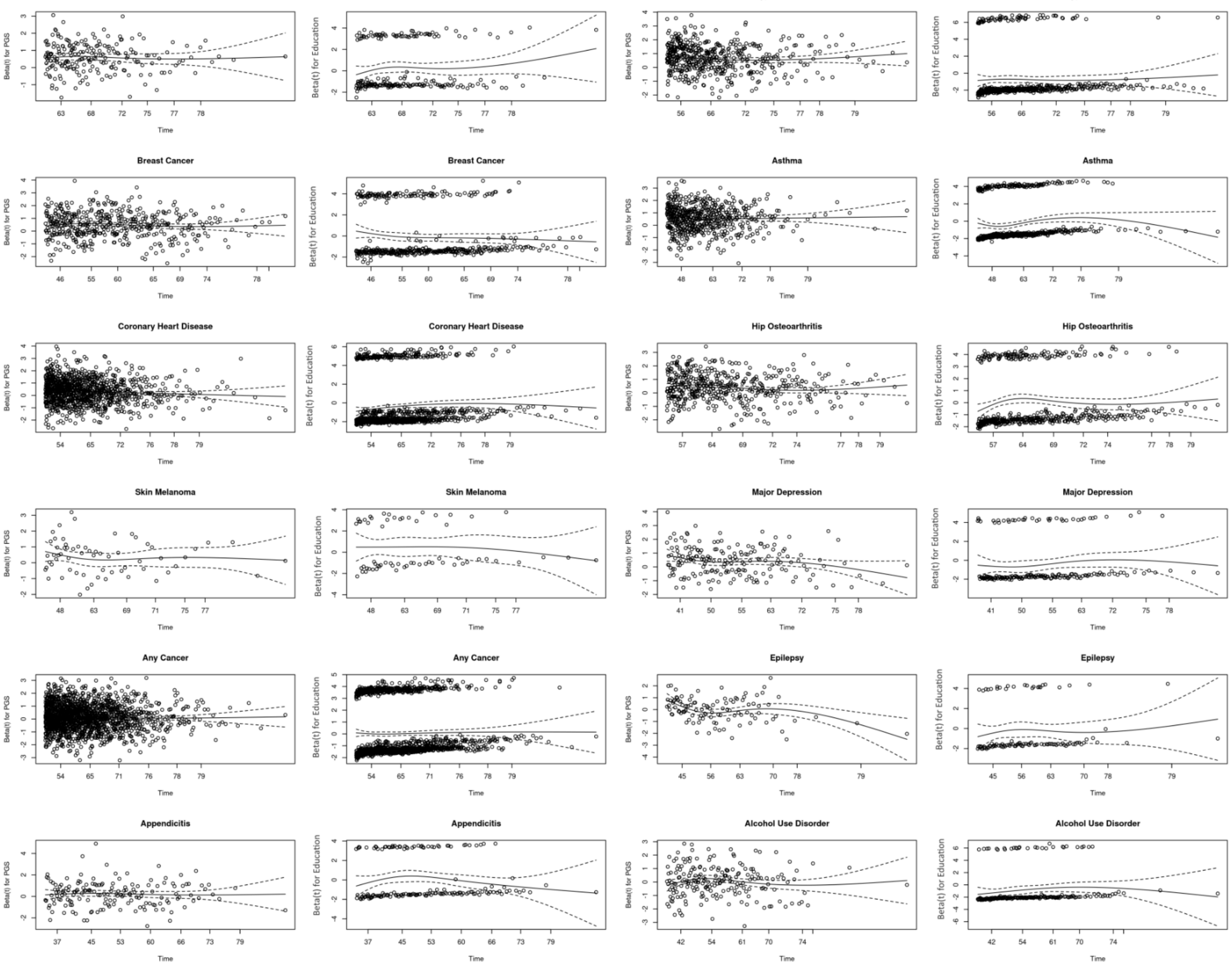

Prostate Cancer
Prostate Cancer
Type 2 Diabetes
Type 2 Diabetes
Breast Cancer
Breast Cancer
Asthma
Asthma
Coronary Heart Disease
Coronary Heart Disease
Hip Osteoarthritis
Hip Osteoarthritis
Skin Melanoma
Skin Melanoma
Major Depression
Major Depression
Any Cancer
Any Cancer
Epilepsy
Epilepsy
Appendicitis
Appendicitis
Alcohol Use Disorder
Alcohol Use Disorder
Beta(t) for PGS
Beta(t) for Education
Time

9

Supplementary Fig. 2. Scaled Schoenfeld residuals of Cox proportional hazard models including the disease-specific polygenic score, educational attainment, and their interaction. **A.** Scaled Schoenfeld residuals in FinnGen study. **B.** Scaled Schoenfeld residuals in the UK Biobank. **C.** Scaled Schoenfeld residuals in Generation Scotland. Note that scaled Schoenfeld residuals could not be generated for Type 1 Diabetes, knee osteoarthritis, and lung cancer due to singularity problems.

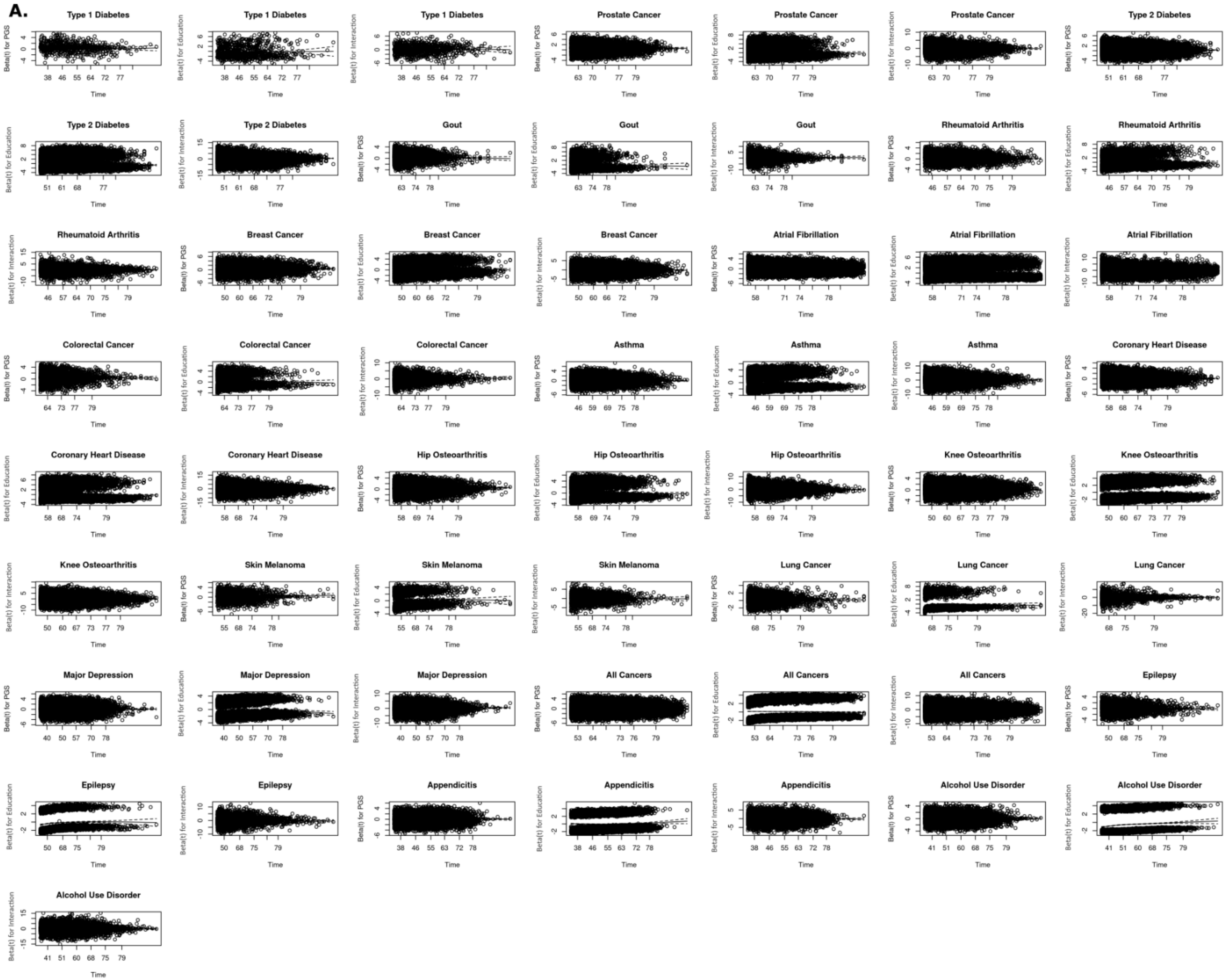

A.
Type 1 Diabetes
Type 1 Diabetes
Type 1 Diabetes
Prostate Cancer
Prostate Cancer
Prostate Cancer
Type 2 Diabetes
Type 2 Diabetes
Type 2 Diabetes
Gout
Gout
Gout
Rheumatoid Arthritis
Rheumatoid Arthritis
Rheumatoid Arthritis
Breast Cancer
Breast Cancer
Breast Cancer
Atrial Fibrillation
Atrial Fibrillation
Atrial Fibrillation
Colorectal Cancer
Colorectal Cancer
Colorectal Cancer
Asthma
Asthma
Asthma
Coronary Heart Disease
Coronary Heart Disease
Coronary Heart Disease
Hip Osteoarthritis
Hip Osteoarthritis
Hip Osteoarthritis
Knee Osteoarthritis
Knee Osteoarthritis
Knee Osteoarthritis
Skin Melanoma
Skin Melanoma
Skin Melanoma
Lung Cancer
Lung Cancer
Lung Cancer
Major Depression
Major Depression
Major Depression
All Cancers
All Cancers
All Cancers
Epilepsy
Epilepsy
Epilepsy
Appendicitis
Appendicitis
Appendicitis
Alcohol Use Disorder
Alcohol Use Disorder
Alcohol Use Disorder

B.

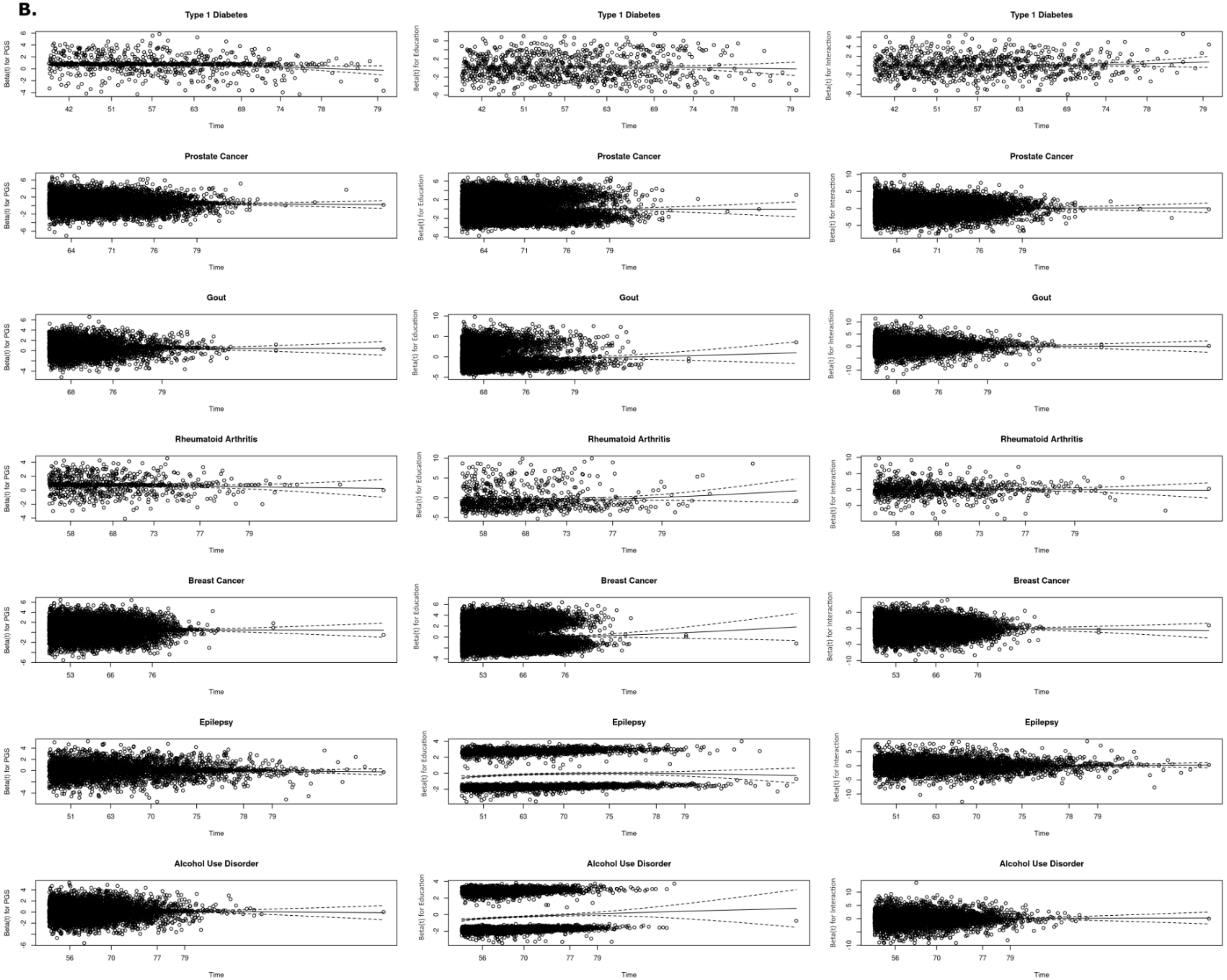
Type 1 Diabetes
Beta(t) for PGS
Beta(t) for Education
Beta(t) for Interaction
Time
Prostate Cancer
Beta(t) for PGS
Beta(t) for Education
Beta(t) for Interaction
Time
Gout
Beta(t) for PGS
Beta(t) for Education
Beta(t) for Interaction
Time
Rheumatoid Arthritis
Beta(t) for PGS
Beta(t) for Education
Beta(t) for Interaction
Time
Breast Cancer
Beta(t) for PGS
Beta(t) for Education
Beta(t) for Interaction
Time
Epilepsy
Beta(t) for PGS
Beta(t) for Education
Beta(t) for Interaction
Time
Alcohol Use Disorder
Beta(t) for PGS
Beta(t) for Education
Beta(t) for Interaction
Time

16

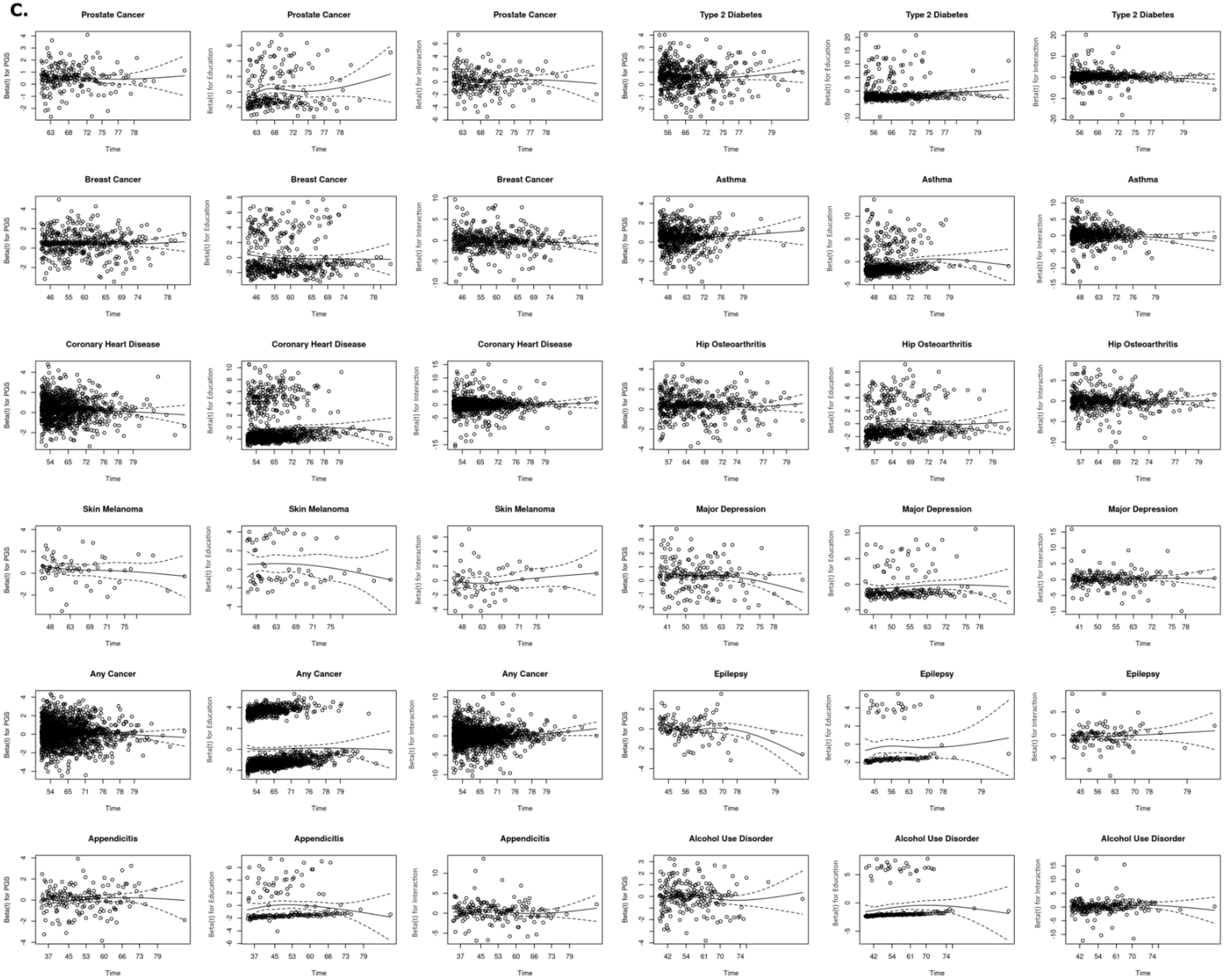
c.
Prostate Cancer
Type 2 Diabetes
Breast Cancer
Asthma
Coronary Heart Disease
Hip Osteoarthritis
Skin Melanoma
Major Depression
Any Cancer
Epilepsy
Appendicitis
Alcohol Use Disorder
Beta(t) for PGS
Beta(t) for Education
Beta(t) for Interaction
Time

17

Supplementary Fig. 3. Fixed- (FE) and random-effect (RE) meta-analyzed and biobank study-specific hazard ratios per standard deviation of the disease-specific polygenic scores (black) and for individuals with high education relative to individuals with low education (purple) on complex disease risk where the disease-specific polygenic scores and education were modelled separately. The asterisk indicates the complex disease was not meta-analyzed and assessed only in the FinnGen study (see **Methods** for details). The exact values are in **Supplementary Data 5**. Sample sizes by case-control status and educational attainment for each complex disease in each biobank study are in **Supplementary Data 4**.

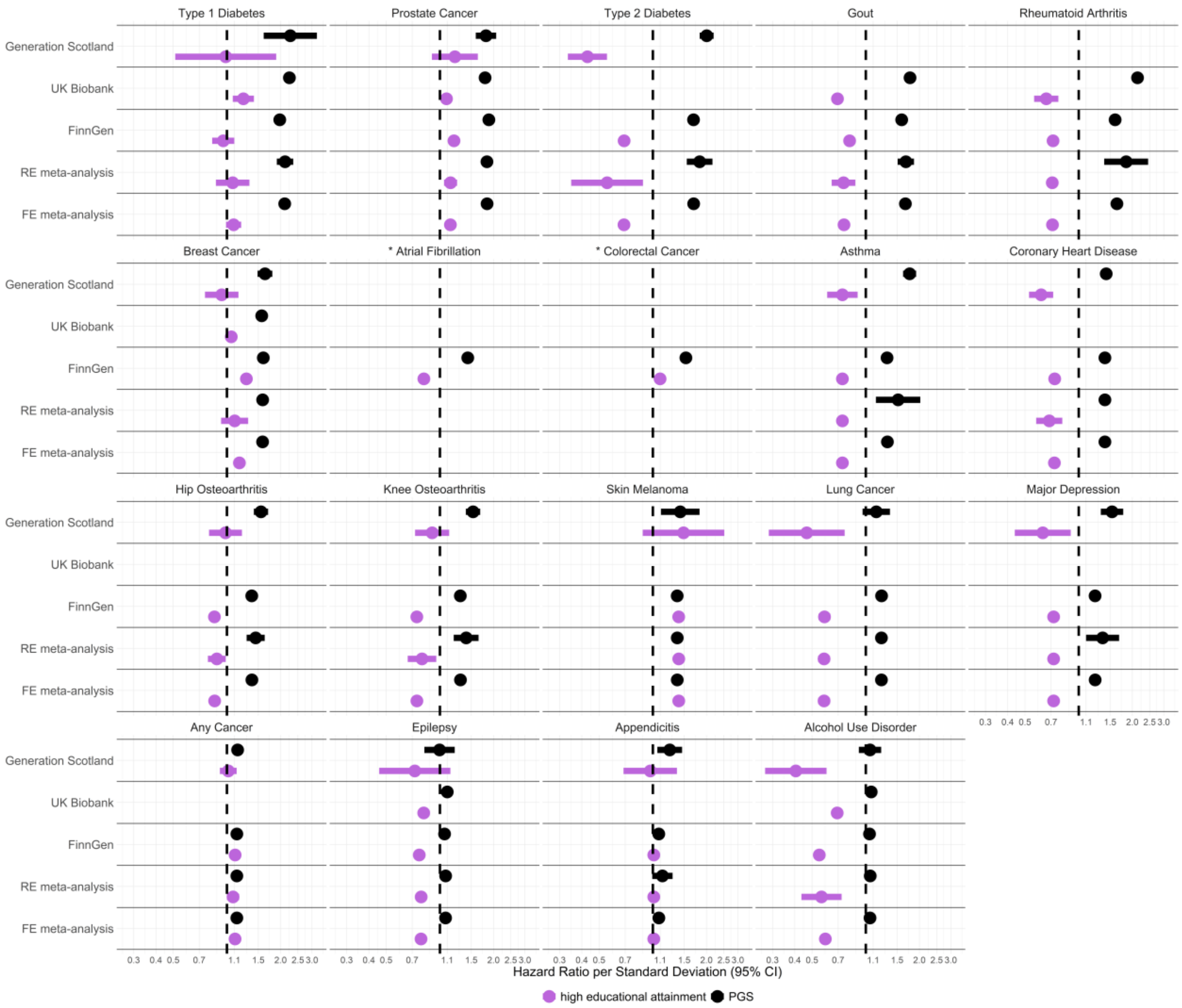

Supplementary Fig. 4. Comparison of the fixed-effect meta-analyzed hazard ratio per standard deviation of the disease-specific polygenic scores on complex disease risk unadjusted (x-axis) and adjusted (y-axis) for the effect of high educational attainment (**A**), comparison of the fixed-effect meta-analyzed hazard ratio for individuals with high education relative to individuals with low education on complex disease risk unadjusted (x-axis) and adjusted (y-axis) for the disease-specific polygenic score (**B**), comparison of the random-effect meta-analyzed hazard ratio per standard deviation of the disease-specific polygenic scores on complex disease risk unadjusted (x-axis) and adjusted (y-axis) for the effect of high educational attainment (**C**), and comparison of the random-effect meta-analyzed hazard ratio for individuals with high education relative to individuals with low education on complex disease risk unadjusted (x-axis) and adjusted (y-axis) for the disease-specific polygenic score (**D**). The asterisk indicates the complex disease was not meta-analyzed and assessed only in the FinnGen study (see **Methods** for details). The exact values are in **Supplementary Data 5-7**. Sample sizes by case-control status and educational attainment for each complex disease in each biobank study are in **Supplementary Data 4**.

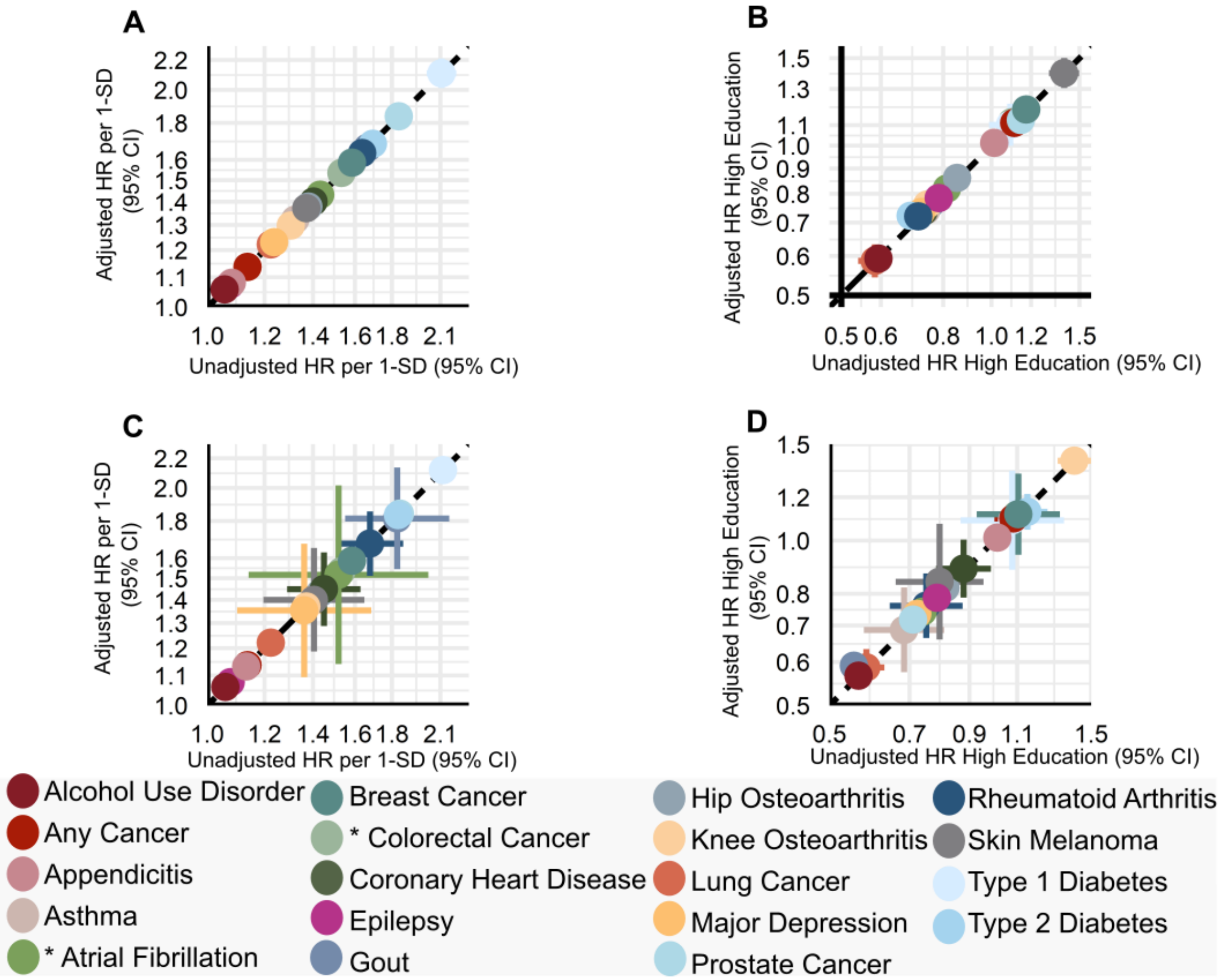

Supplementary Fig. 5. Fixed- (FE) and random-effect (RE) meta-analyzed and biobank study-specific hazard ratios per standard deviation of the disease-specific polygenic scores (black) and for individuals with high education relative to individuals with low education (purple) on complex disease risk where the disease-specific polygenic scores and education were modelled jointly. The asterisk indicates the complex disease was not meta-analyzed and assessed only in the FinnGen study (see **Methods** for details). The exact values are in **Supplementary Data 6**. Sample sizes by case-control status and educational attainment for each complex disease in each biobank study are in **Supplementary Data 4**.

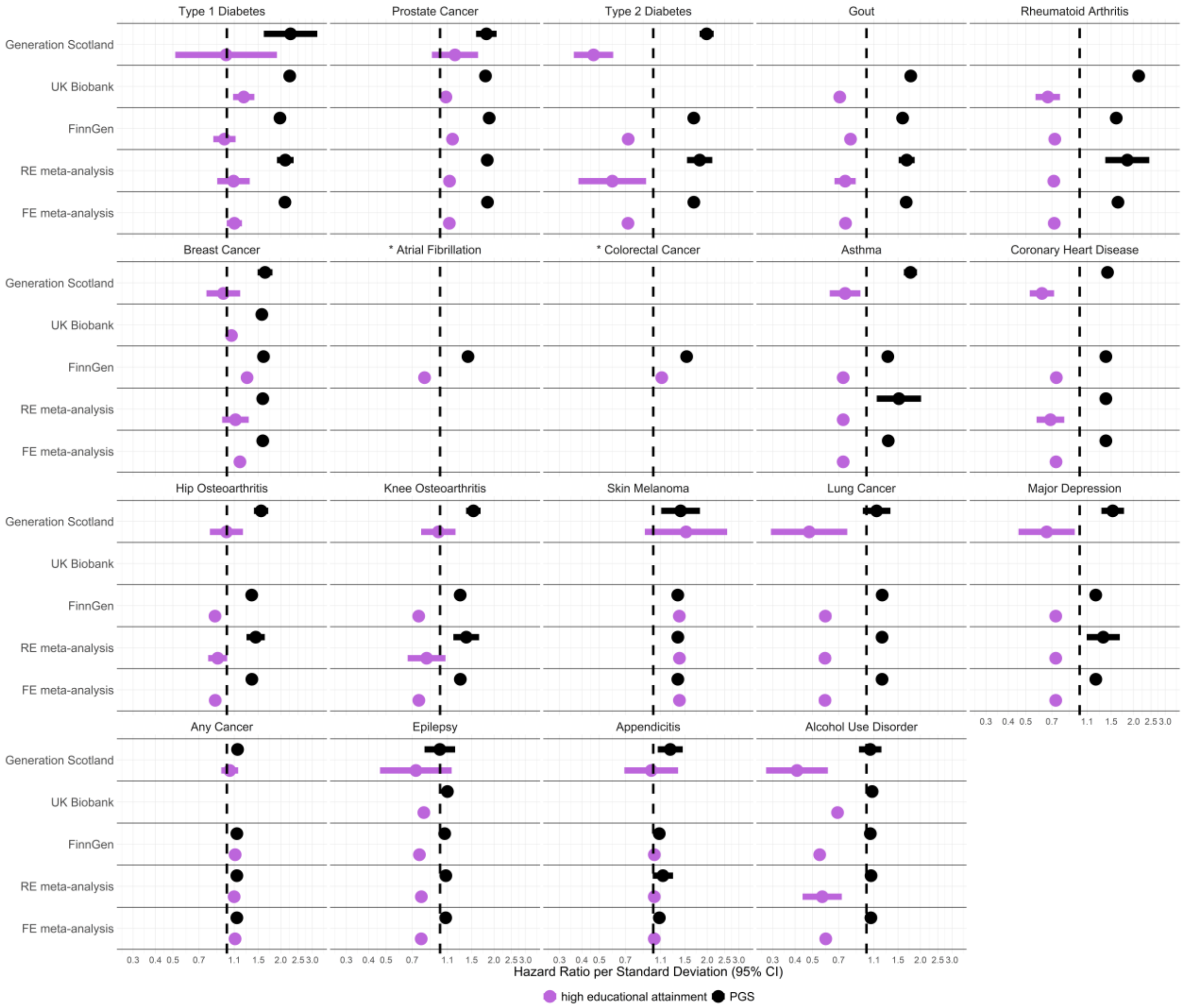

Supplementary Fig. 6. Fixed- (FE) and random-effect (RE) meta-analyzed and biobank study-specific hazard ratios per standard deviation of the disease-specific polygenic scores by educational attainment on complex disease risk. The asterisk indicates the complex disease was not meta-analyzed and assessed only in the FinnGen study (see **Methods** for details). The exact values are in **Supplementary Data 8**. Sample sizes by case-control status and educational attainment for each complex disease in each biobank are in **Supplementary Data 4**.

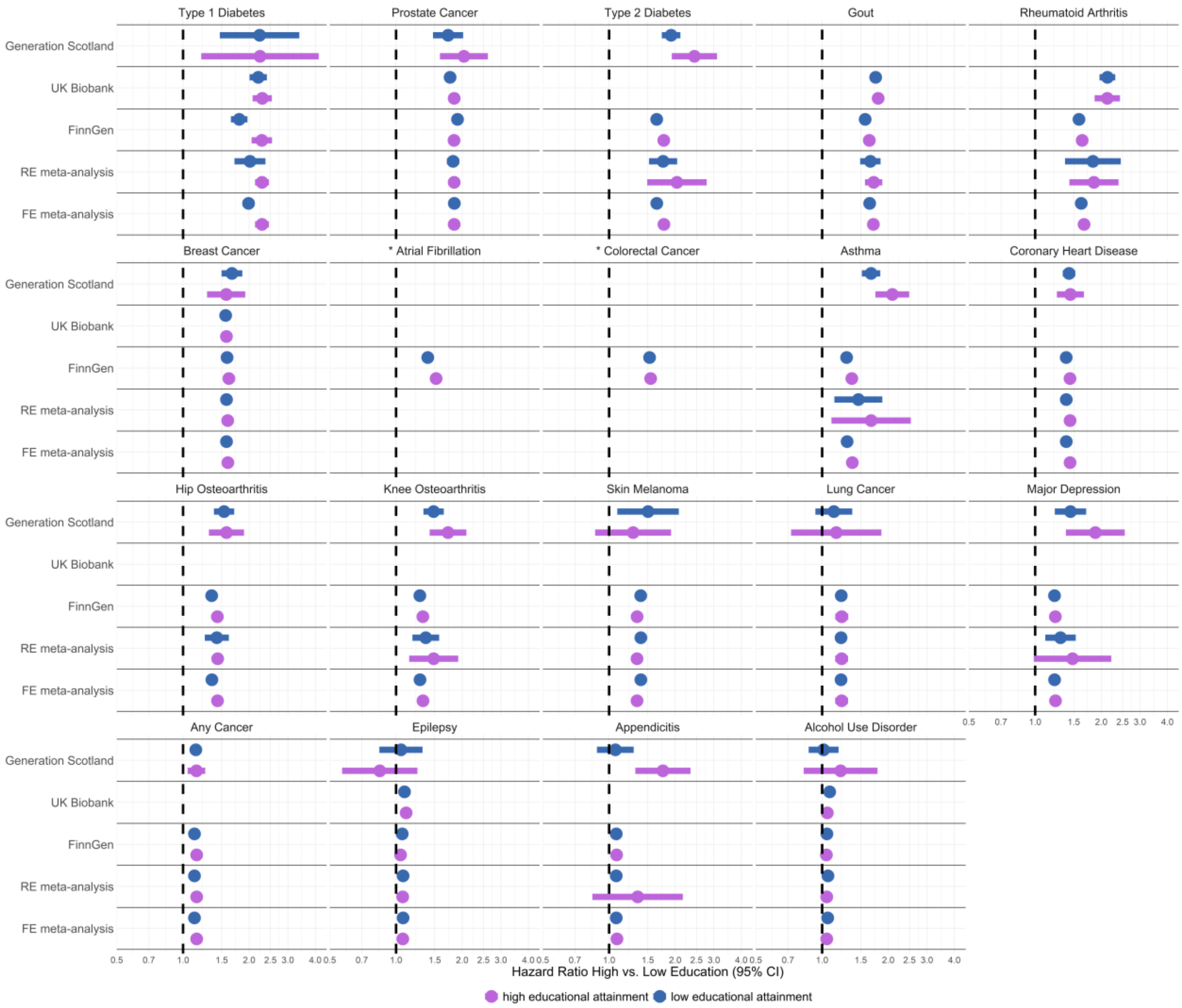

Supplementary Fig. 7. Educational attainment-specific cumulative incidence estimates in FinnGen. Bootstrapped 95% confidence intervals reflect the uncertainty of the cumulative incidence estimates for the top, median, and bottom of the disease-specific PGS distribution for (**A**) asthma, (**B**) any cancer, (**C**) coronary heart disease, (**D**) hip osteoarthritis, (**E**) knee osteoarthritis, (**F**) prostate cancer, and (**G**) type 1 diabetes. The exact values are in **Supplementary Data 12**, the exact values of the underlying Cox proportional hazard model are in **Supplementary Data 11**, and the sample sizes by case-control status and educational attainment are in **Supplementary Data 10**.

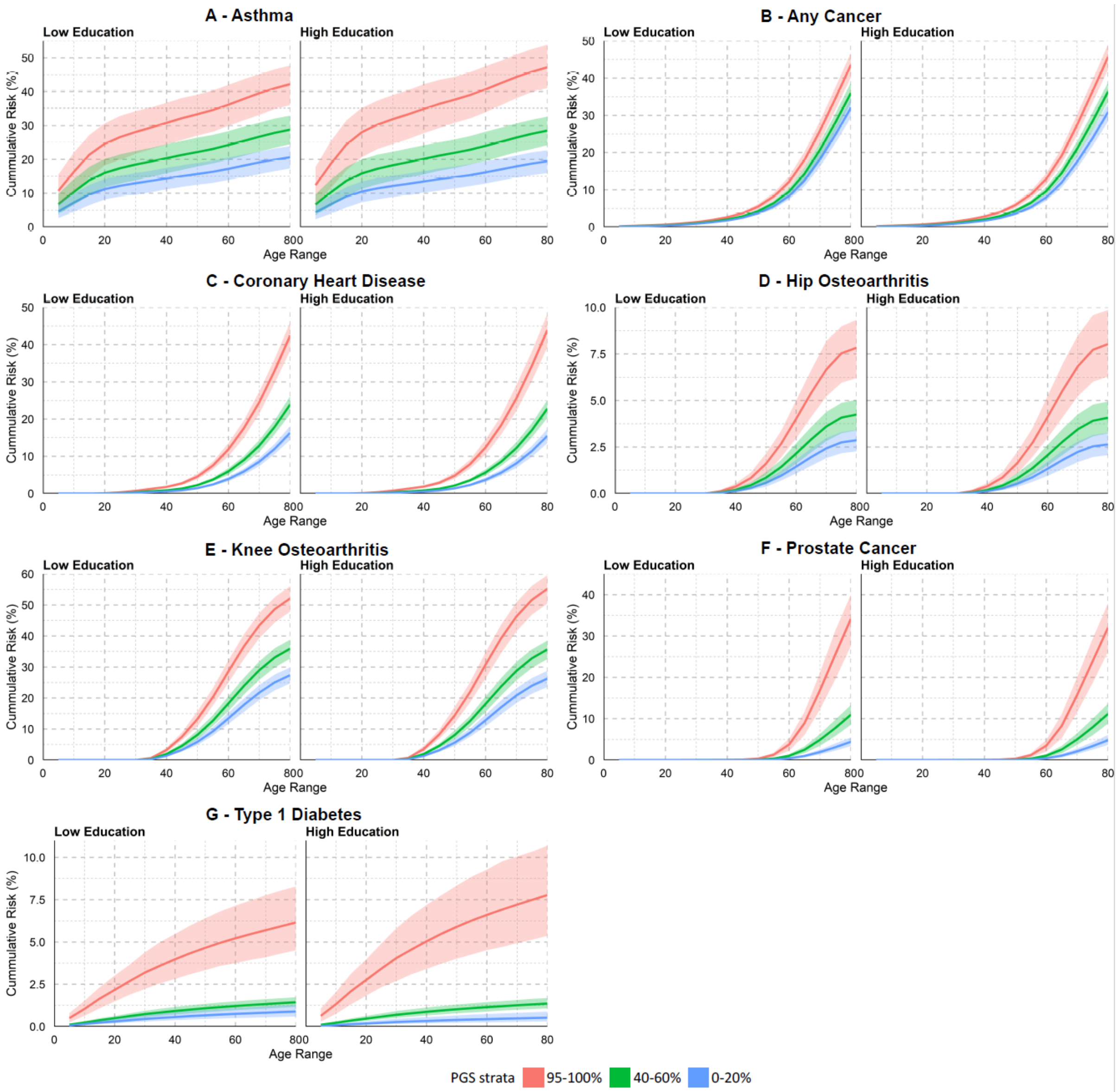

Supplementary Fig. 8. Fixed- (FE) and random-effect (RE) meta-analyzed and biobank study-specific hazard ratios per standard deviation of the disease-specific polygenic scores (black) and for individuals with upper-level occupation relative to individuals with lower-level occupation (purple) on complex disease risk where the disease-specific polygenic scores and occupation were modelled separately. The asterisk indicates the complex disease was not meta-analyzed and assessed only in the FinnGen study (see **Methods** for details). The exact values are in **Supplementary Data 22**. Sample sizes by case-control status and occupation for each complex disease in each biobank study are in **Supplementary Data 21**.

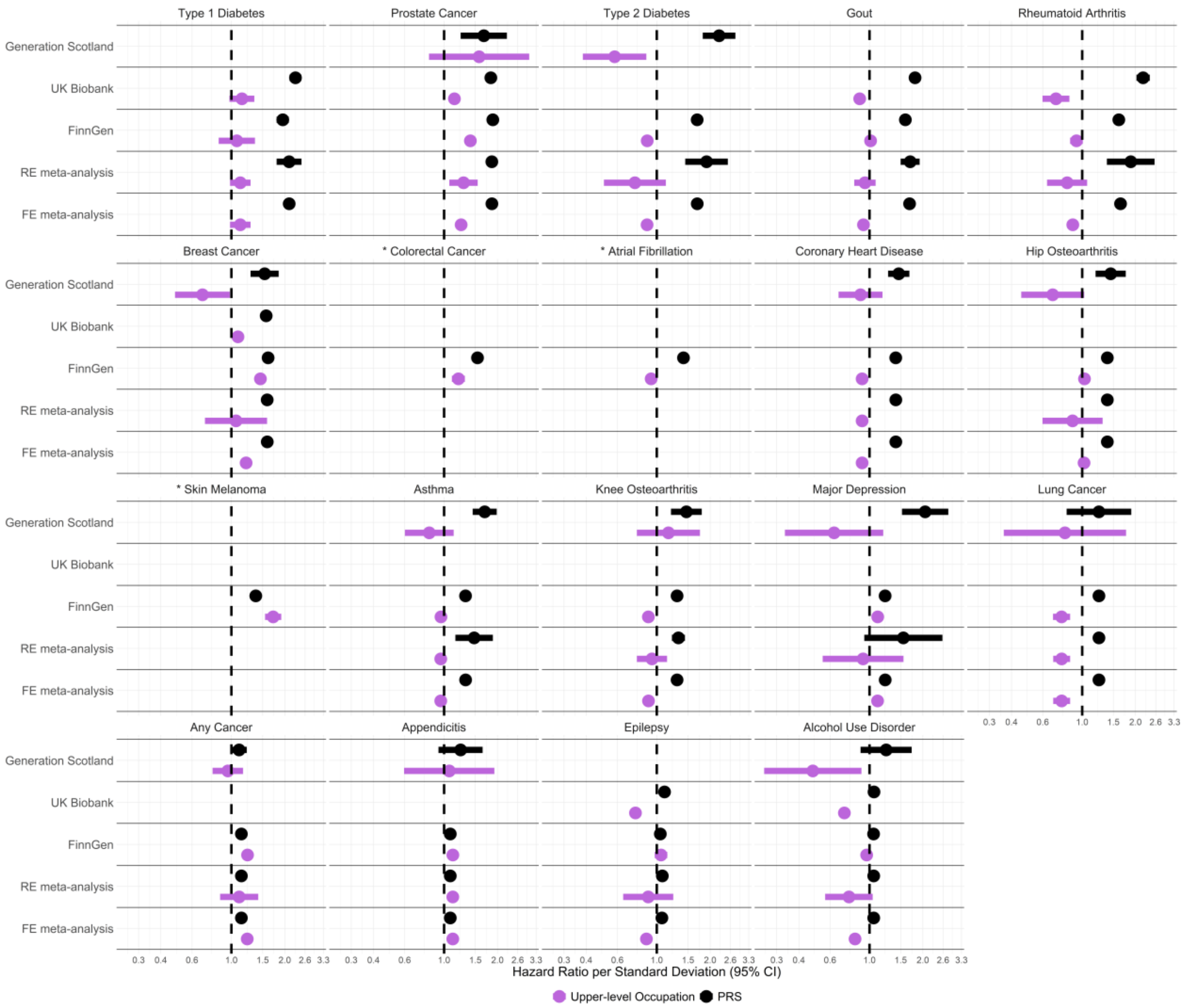

Supplementary Fig. 9. Comparison of the fixed-effect meta-analyzed hazard ratio per standard deviation of the disease-specific polygenic scores on complex disease risk unadjusted (x-axis) and adjusted (y-axis) for the effect of upper-level occupation (**A**), comparison of the fixed-effect meta-analyzed hazard ratio for individuals with upper-level occupation relative to individuals with lower-level occupation on complex disease risk unadjusted (x-axis) and adjusted (y-axis) for the disease-specific polygenic score (**B**), comparison of the random-effect meta-analyzed hazard ratio per standard deviation of the disease-specific polygenic scores on complex disease risk unadjusted (x-axis) and adjusted (y-axis) for the effect of upper-level occupation (**C**), and comparison of the random-effect meta-analyzed hazard ratio for individuals with upper-level occupation relative to individuals with lower-level occupation on complex disease risk unadjusted (x-axis) and adjusted (y-axis) for the disease-specific polygenic score (**D**). The asterisk indicates the complex disease was not meta-analyzed and assessed only in the FinnGen study (see **Methods** for details). The exact values are in **Supplementary Data 22-24**. Sample sizes by case-control status and educational attainment for each complex disease in each biobank study are in **Supplementary Data 21**.

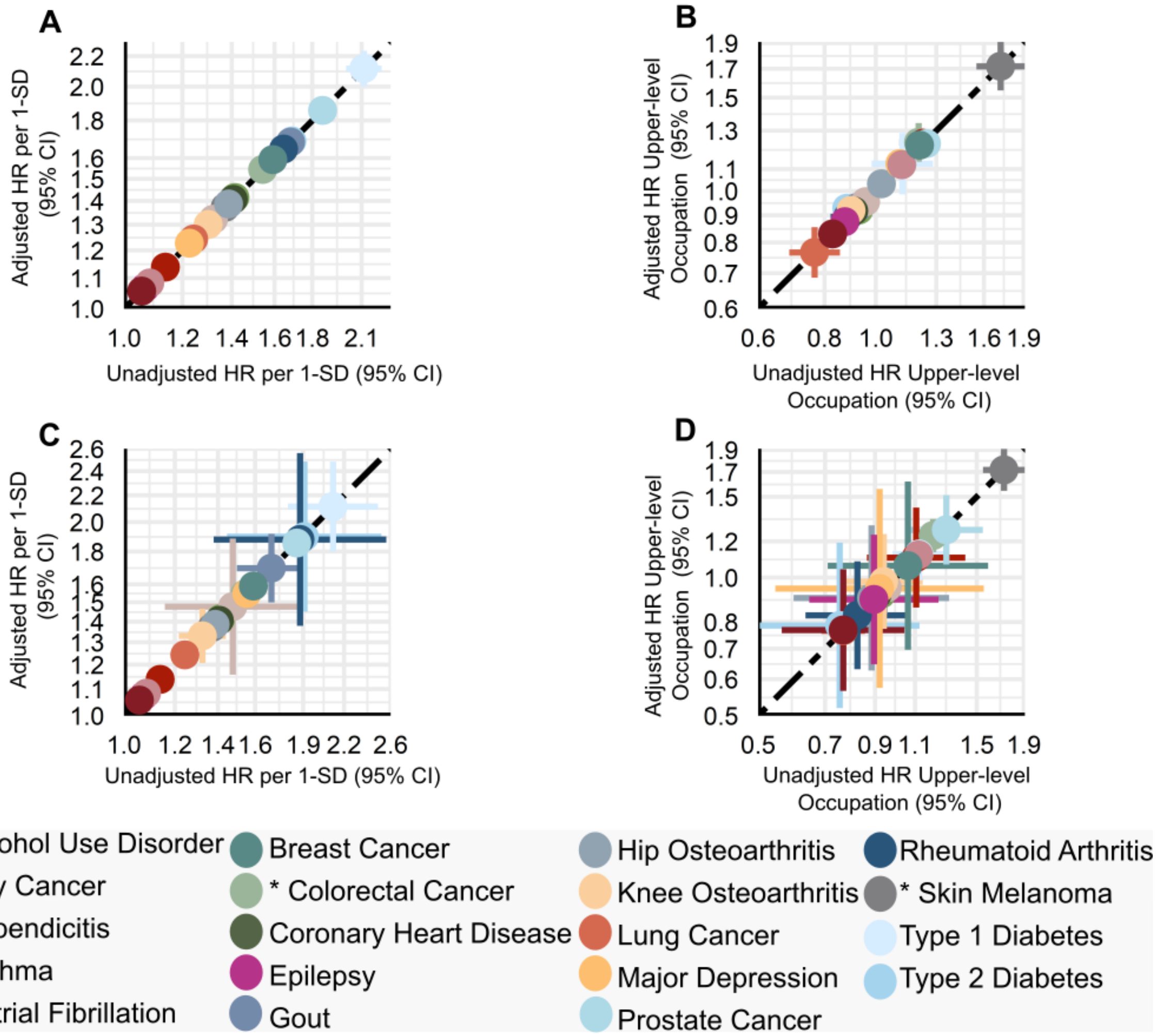

Supplementary Fig. 10. Comparison of the fixed-effect meta-analyzed hazard ratios across the educational attainment and occupation models. **A.** Comparison of the fixed-effect meta-analyzed hazard ratio for individuals with high education relative to individuals with low education on complex disease risk (x-axis) against fixed-effect meta-analyzed hazard ratio for individuals with upper-level occupation relative to individuals with lower-level occupation on complex disease risk (y-axis). **B. F**ixed-effect meta-analyzed hazard ratio per standard deviation of the disease-specific polygenic scores on complex disease risk in individuals with educational attainment available (x-axis) compared to individuals with occupation available (y-axis). **C**. Comparison of the fixed-effect meta-analyzed hazard ratio per standard deviation of the disease-specific polygenic score in the low education (x-axis) versus lower-level occupation (y-axis) groups. **D.** Comparison of the fixed-effect meta-analyzed hazard ratio per standard deviation of the disease-specific polygenic score for individuals in the high education (x-axis) versus upper-level occupation (y-axis) group. The exact values are in **Supplementary Data 5**, **8**, **22, 25-26**, and **28**. Sample sizes by case-control status and educational attainment or occupation for each complex disease in each biobank study are in **Supplementary Data 4** and **21**.

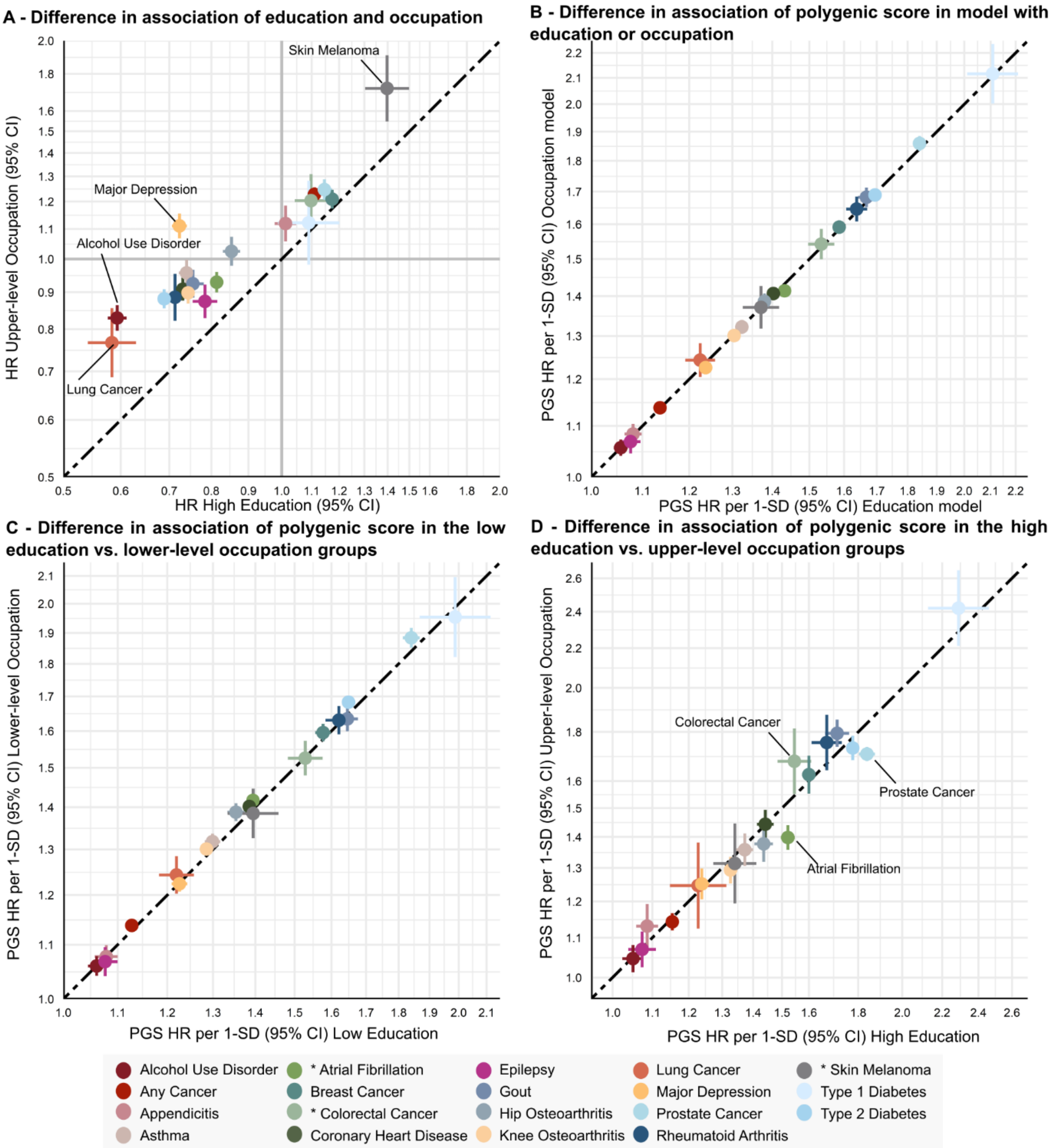
A - Difference in association of education and occupation
HR Upper-level Occupation (95% CI)
HR High Education (95% CI)
Skin Melanoma
Major Depression
Alcohol Use Disorder
Lung Cancer
B - Difference in association of polygenic score in model with education or occupation
PGS HR per 1-SD (95% CI) Occupation model
PGS HR per 1-SD (95% CI) Education model
C - Difference in association of polygenic score in the low education vs. lower-level occupation groups
PGS HR per 1-SD (95% CI) Lower-level Occupation
PGS HR per 1-SD (95% CI) Low Education
D - Difference in association of polygenic score in the high education vs. upper-level occupation groups
PGS HR per 1-SD (95% CI) Upper-level Occupation
PGS HR per 1-SD (95% CI) High Education
Colorectal Cancer
Prostate Cancer
Atrial Fibrillation
Alcohol Use Disorder
Any Cancer
Appendicitis
Asthma
* Atrial Fibrillation
Breast Cancer
* Colorectal Cancer
Coronary Heart Disease
Epilepsy
Gout
Hip Osteoarthritis
Knee Osteoarthritis
Lung Cancer
Major Depression
Prostate Cancer
Rheumatoid Arthritis
* Skin Melanoma
Type 1 Diabetes
Type 2 Diabetes

Supplementary Fig. 11. Comparison of the random-effect meta-analyzed hazard ratios across the educational attainment and occupation models. **A.** Comparison of the random-effect meta-analyzed hazard ratio for individuals with high education relative to individuals with low education on complex disease risk (x-axis) against random-effect meta-analyzed hazard ratio for individuals with upper-level occupation relative to individuals with lower-level occupation on complex disease risk (y-axis). **B.** Random-effect meta-analyzed hazard ratio per standard deviation of the disease-specific polygenic scores on complex disease risk in individuals with educational attainment available (x-axis) compared to individuals with occupation available (y-axis). **C**. Comparison of the random-effect meta-analyzed hazard ratio per standard deviation of the disease-specific polygenic score in the low education (x-axis) versus lower-level occupation (y-axis) groups. **D.** Comparison of the random-effect meta-analyzed hazard ratio per standard deviation of the disease-specific polygenic score for individuals in the high education (x-axis) versus upper-level occupation (y-axis) group. The exact values are in **Supplementary Data 5**, **8**, **22, 25-26**, and **28**. Sample sizes by case-control status and educational attainment or occupation for each complex disease in each biobank study are in **Supplementary Data 4** and **21**.

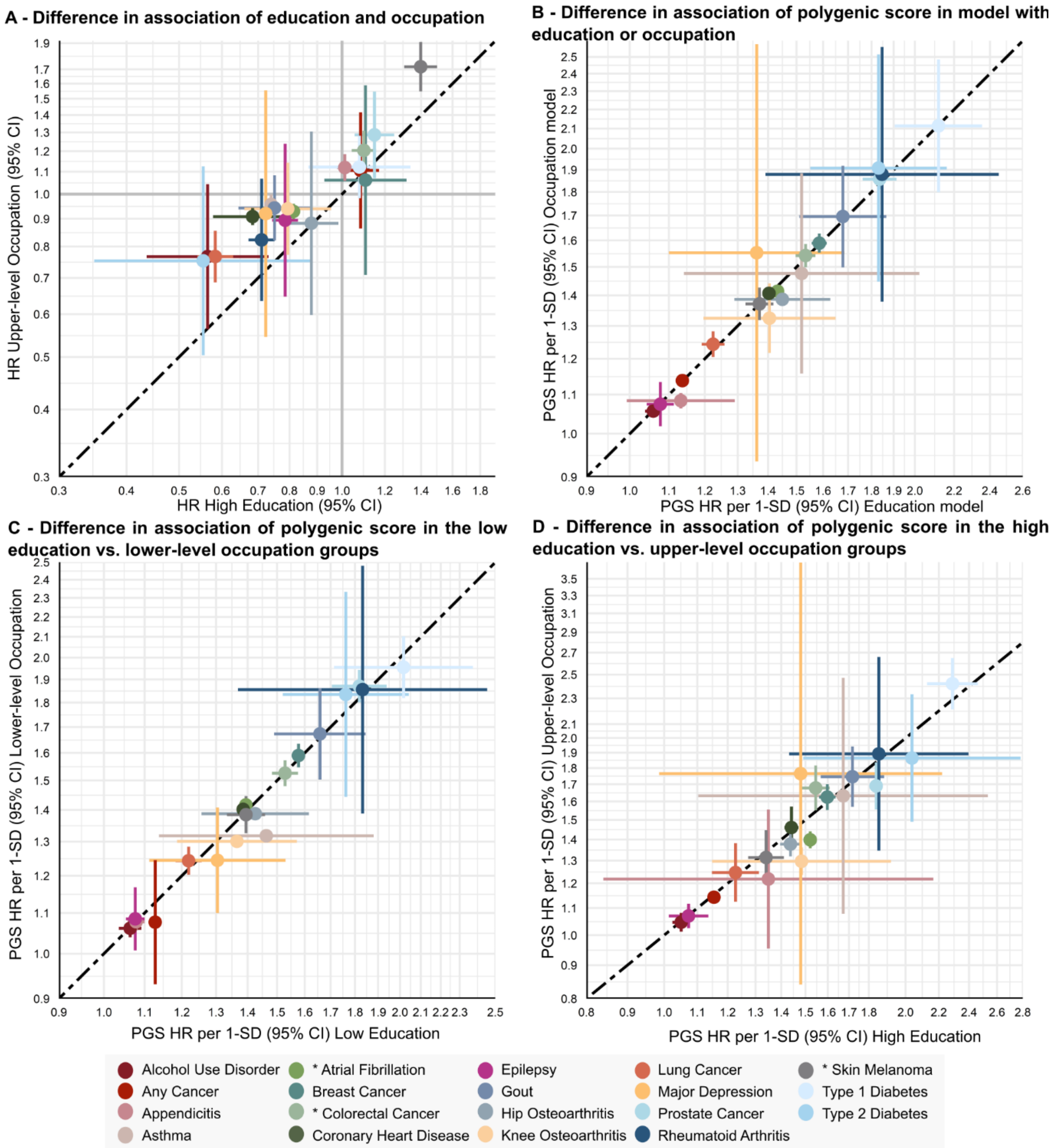
A - Difference in association of education and occupation
HR Upper-level Occupation (95% CI)
HR High Education (95% CI)
B - Difference in association of polygenic score in model with education or occupation
PGS HR per 1-SD (95% CI) Occupation model
PGS HR per 1-SD (95% CI) Education model
C - Difference in association of polygenic score in the low education vs. lower-level occupation groups
PGS HR per 1-SD (95% CI) Lower-level Occupation
PGS HR per 1-SD (95% CI) Low Education
D - Difference in association of polygenic score in the high education vs. upper-level occupation groups
PGS HR per 1-SD (95% CI) Upper-level Occupation
PGS HR per 1-SD (95% CI) High Education
Alcohol Use Disorder
Any Cancer
Appendicitis
Asthma
* Atrial Fibrillation
Breast Cancer
* Colorectal Cancer
Coronary Heart Disease
Epilepsy
Gout
Hip Osteoarthritis
Knee Osteoarthritis
Lung Cancer
Major Depression
Prostate Cancer
Rheumatoid Arthritis
* Skin Melanoma
Type 1 Diabetes
Type 2 Diabetes

121

Supplementary Fig. 12. Fixed- (FE) and random-effect (RE) meta-analyzed and biobank study-specific hazard ratios per standard deviation of the disease-specific polygenic scores by occupation on complex disease risk. The asterisk indicates the complex disease was not meta-analyzed and assessed only in the FinnGen study (see **Methods** for details). The exact values are in **Supplementary Data 26**. Sample sizes by case-control status and educational attainment for each complex disease in each biobank are in **Supplementary Data 21**.

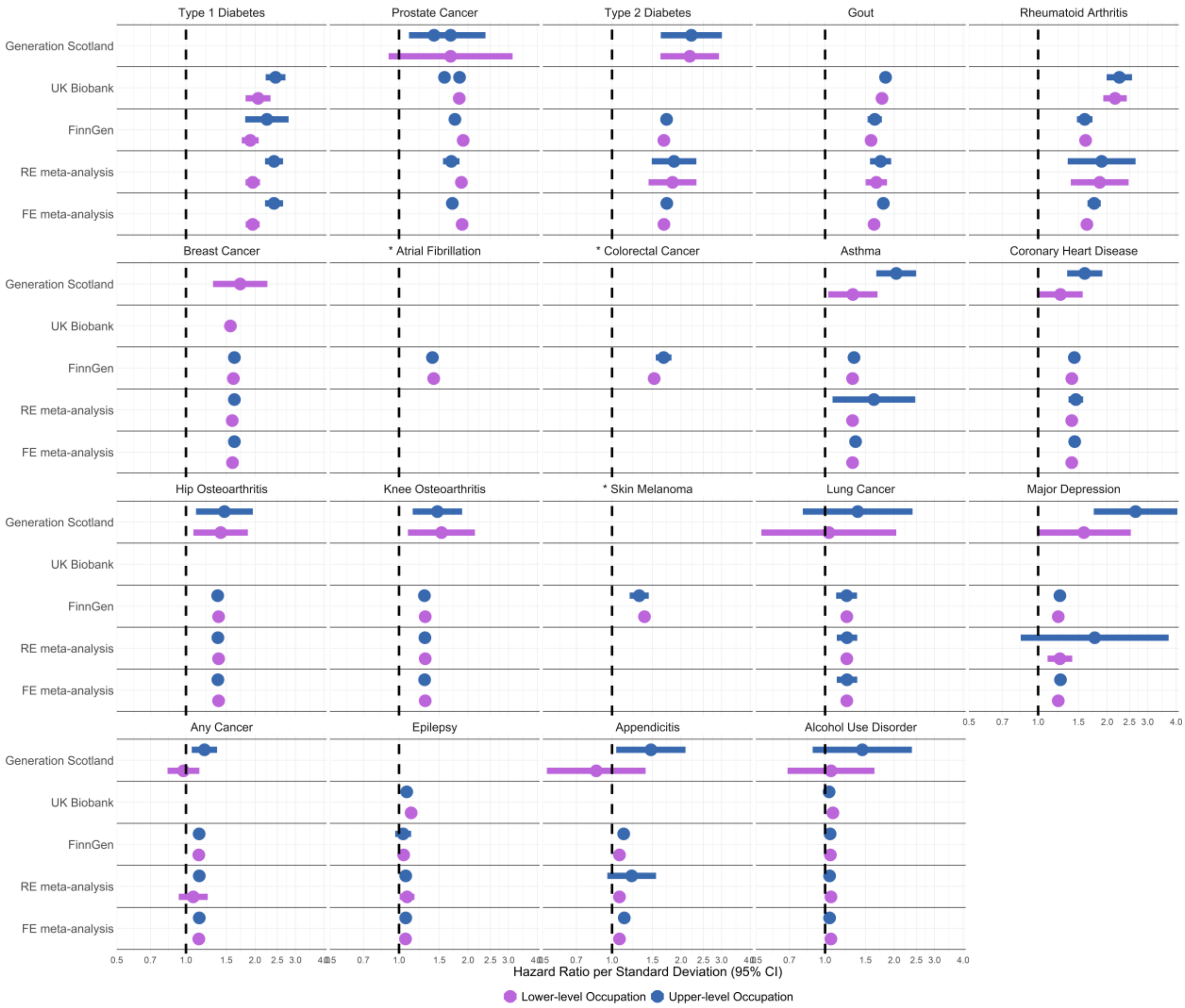

Supplementary Fig. 13. Occupation-specific cumulative incidence estimates in FinnGen. Bootstrapped 95% confidence intervals reflect the uncertainty of the cumulative incidence estimates for the top, median, and bottom of the disease-specific PGS distribution for (**A**) asthma, (**B**) any cancer, (**C**) coronary heart disease, (**D**) hip osteoarthritis, (**E**) knee osteoarthritis, (**F**) prostate cancer, (**G**) type 2 diabetes, and (**H**) atrial fibrillation. The exact values are in **Supplementary Data 31**, the exact values of the underlying Cox proportional hazard model are in **Supplementary Data 30**, and the sample sizes by case-control status and occupation are in **Supplementary Data 29**.

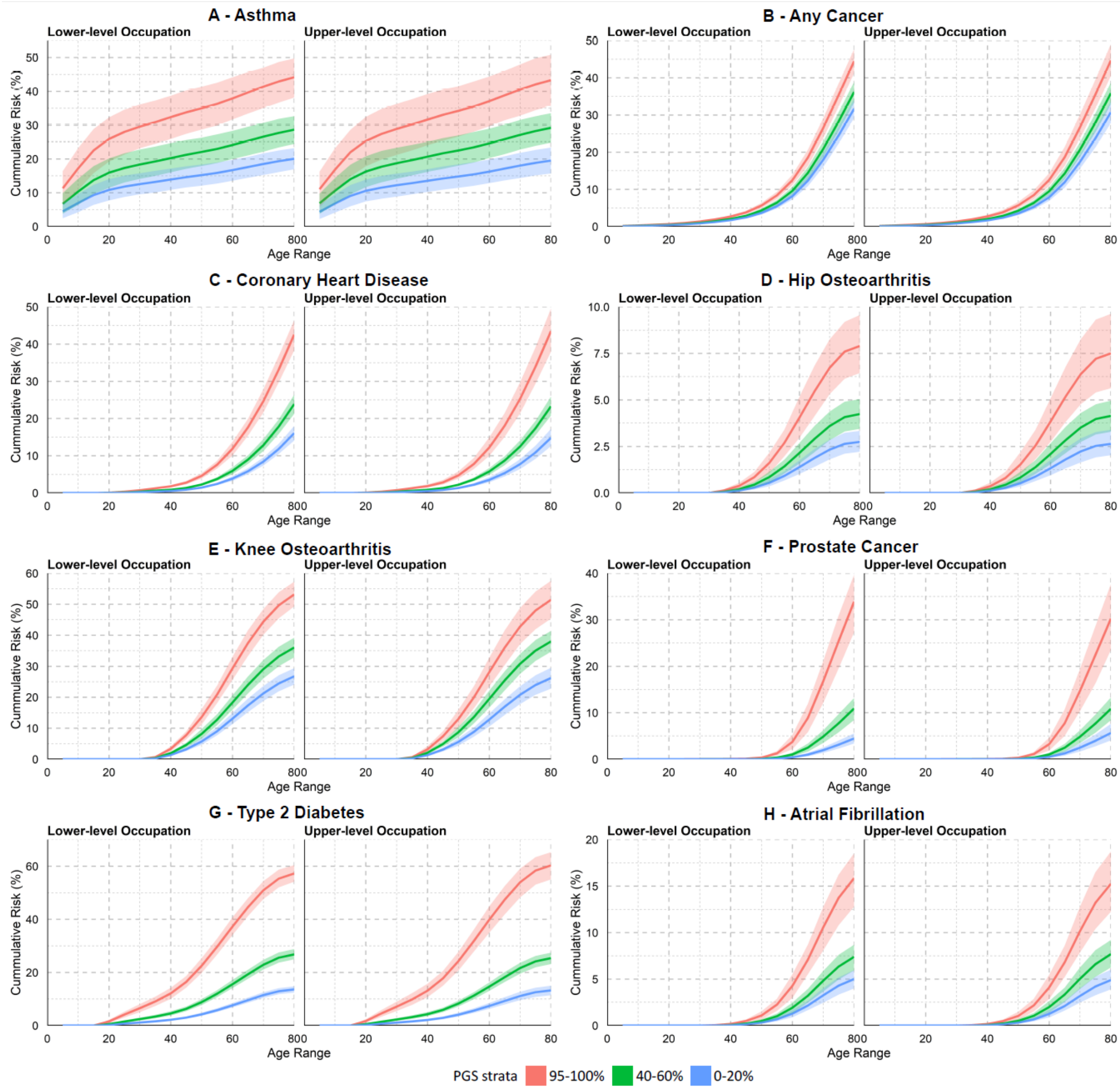

Supplementary Fig. 14. Comparison of the meta-analyzed hazard ratios per standard deviation of the disease-specific polygenic scores, for individuals with high education relative to individuals with low education, and for the interaction between the disease-specific polygenic scores and educational attainment when applying a uniform dichotomization cutoff for educational attainment (ISCED 5) versus cohort-specific cutoffs. **A.** Comparison of the meta-analyzed fixed-effect hazard ratio for individuals with high education relative to low education with the same cutoff (x-axis) versus the cohort-specific cutoff (y-axis). **B.** Comparison of the meta-analyzed fixed-effect hazard ratios per standard deviation of the disease-specific polygenic scores on disease risk with the same cutoff (x-axis) versus the cohort-specific cutoff (y-axis). **C.** Comparison of the meta-analyzed fixed-effect hazard ratio of the interaction between the disease-specific polygenic score and educational attainment with the same cutoff (x-axis) versus the cohort-specific cutoff (y-axis). **D.** Comparison of the meta-analyzed random-effect hazard ratio for individuals with high education relative to low education with the same cutoff (x-axis) versus the cohort-specific cutoff (y-axis). **E.** Comparison of the meta-analyzed random-effect hazard ratios per standard deviation of the disease-specific polygenic scores on disease risk with the same cutoff (x-axis) versus the cohort-specific cutoff (y-axis). **F.** Comparison of the meta-analyzed random-effect hazard ratio of the interaction between the disease-specific polygenic score and educational attainment with the same cutoff (x-axis) versus the cohort-specific cutoff (y-axis). The exact values are in **Supplementary Data 9** and **33-34**. Sample sizes by case-control status and educational attainment for each complex disease are in **Supplementary Data 4** and **32**.

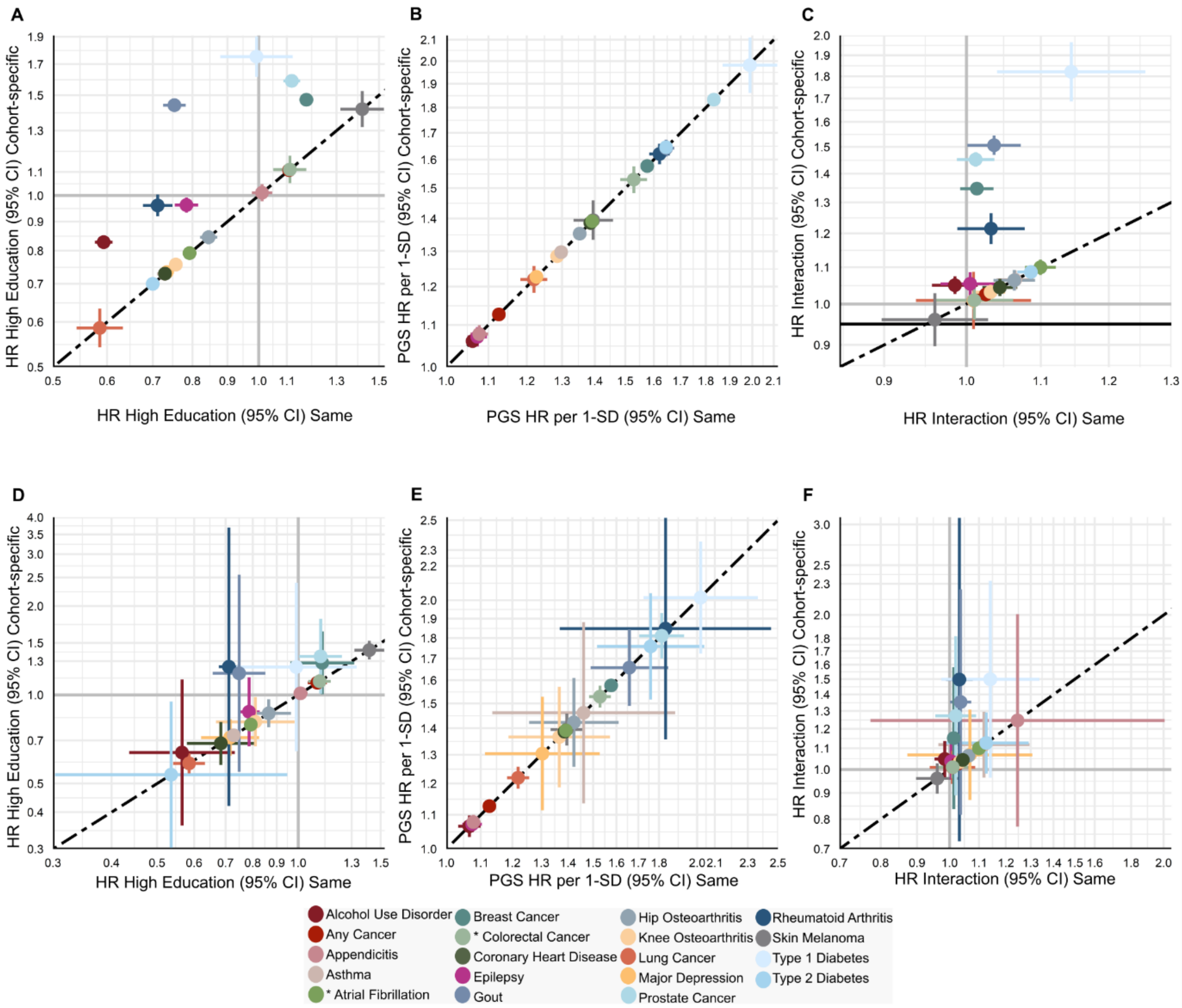
A
HR High Education (95% CI) Cohort-specific
HR High Education (95% CI) Same
B
PGS HR per 1-SD (95% CI) Cohort-specific
PGS HR per 1-SD (95% CI) Same
C
HR Interaction (95% CI) Cohort-specific
HR Interaction (95% CI) Same
D
HR High Education (95% CI) Cohort-specific
HR High Education (95% CI) Same
E
PGS HR per 1-SD (95% CI) Cohort-specific
PGS HR per 1-SD (95% CI) Same
F
HR Interaction (95% CI) Cohort-specific
HR Interaction (95% CI) Same
Alcohol Use Disorder
Any Cancer
Appendicitis
Asthma
* Atrial Fibrillation
Breast Cancer
* Colorectal Cancer
Coronary Heart Disease
Epilepsy
Gout
Hip Osteoarthritis
Knee Osteoarthritis
Lung Cancer
Major Depression
Prostate Cancer
Rheumatoid Arthritis
Skin Melanoma
Type 1 Diabetes
Type 2 Diabetes

Supplementary Fig. 15. Comparison of the meta-analyzed hazard ratios per standard deviation of the disease-specific polygenic scores, for individuals with high education relative to individuals with low education, and for the interaction between the disease-specific polygenic scores and educational attainment when dichotomizing educational attainment versus when educational attainment is not dichotomized. **A.** Comparison of the meta-analyzed fixed-effect hazard ratio for individuals with high education relative to low education when dichotomizing educational attainment (x-axis) versus when educational attainment is not dichotomized (y-axis). **B.** Comparison of the meta-analyzed fixed-effect hazard ratios per standard deviation of the disease-specific polygenic scores on disease risk when dichotomizing educational attainment (x-axis) versus when educational attainment is not dichotomized (y-axis). **C.** Comparison of the meta-analyzed fixed-effect hazard ratio of the interaction between the disease-specific polygenic score and educational attainment when dichotomizing educational attainment (x-axis) versus when educational attainment is not dichotomized (y-axis). **D.** Comparison of the meta-analyzed random-effect hazard ratio for individuals with high education relative to low education when dichotomizing educational attainment (x-axis) versus when educational attainment is not dichotomized (y-axis). **E.** Comparison of the meta-analyzed random-effect hazard ratios per standard deviation of the disease-specific polygenic scores on disease risk when dichotomizing educational attainment (x-axis) versus when educational attainment is not dichotomized (y-axis). **F.** Comparison of the meta-analyzed random-effect hazard ratio of the interaction between the disease-specific polygenic score and educational attainment when dichotomizing educational attainment (x-axis) versus when educational attainment is not dichotomized (y-axis). The exact values are in **Supplementary Data 9** and **35-36**. Sample sizes by case-control status and educational attainment for each complex disease are in **Supplementary Data 4**.

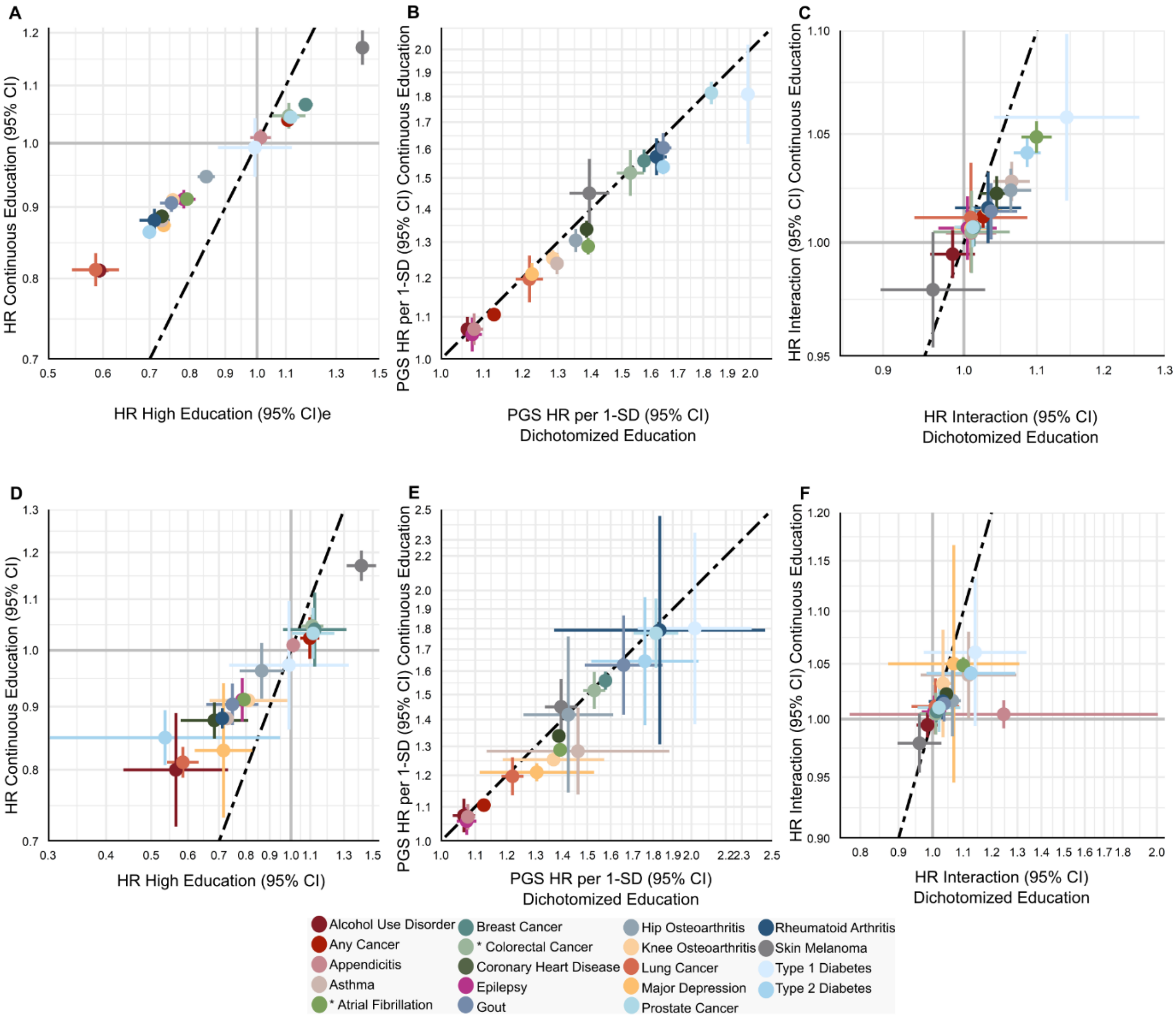
A
HR Continuous Education (95% CI)
HR High Education (95% CI)e
B
PGS HR per 1-SD (95% CI) Continuous Education
PGS HR per 1-SD (95% CI)
Dichotomized Education
C
HR Interaction (95% CI) Continuous Education
HR Interaction (95% CI)
Dichotomized Education
D
HR Continuous Education (95% CI)
HR High Education (95% CI)
E
PGS HR per 1-SD (95% CI) Continuous Education
PGS HR per 1-SD (95% CI)
Dichotomized Education
F
HR Interaction (95% CI) Continuous Education
HR Interaction (95% CI)
Dichotomized Education
Alcohol Use Disorder
Any Cancer
Appendicitis
Asthma
* Atrial Fibrillation
Breast Cancer
* Colorectal Cancer
Coronary Heart Disease
Epilepsy
Gout
Hip Osteoarthritis
Knee Osteoarthritis
Lung Cancer
Major Depression
Prostate Cancer
Rheumatoid Arthritis
Skin Melanoma
Type 1 Diabetes
Type 2 Diabetes

Supplementary Fig. 16. Comparison of the hazard ratios per standard deviation of the disease-specific polygenic scores, for individuals with high education relative to individuals with low education, and for the interaction between the disease-specific polygenic scores and educational attainment when including the full FinnGen sample versus after exclusion of cases before cohort entry in FinnGen. **A.** Comparison of the hazard ratio for individuals with high education relative to low education in the full FinnGen sample (x-axis) versus after exclusion of cases before cohort entry (y-axis). **B.** Comparison of the hazard ratios per standard deviation of the disease-specific polygenic scores on disease risk in the full FinnGen sample (x-axis) versus after exclusion of cases before cohort entry (y-axis). **C.** Comparison of the hazard ratio of the interaction between the disease-specific polygenic score and educational attainment in the full FinnGen sample (x-axis) versus after exclusion of cases before cohort entry (y-axis). The exact values are in **Supplementary Data 9** and **38-39**. Sample sizes by case-control status and educational attainment for each complex disease are in **Supplementary Data 4** and **37**.

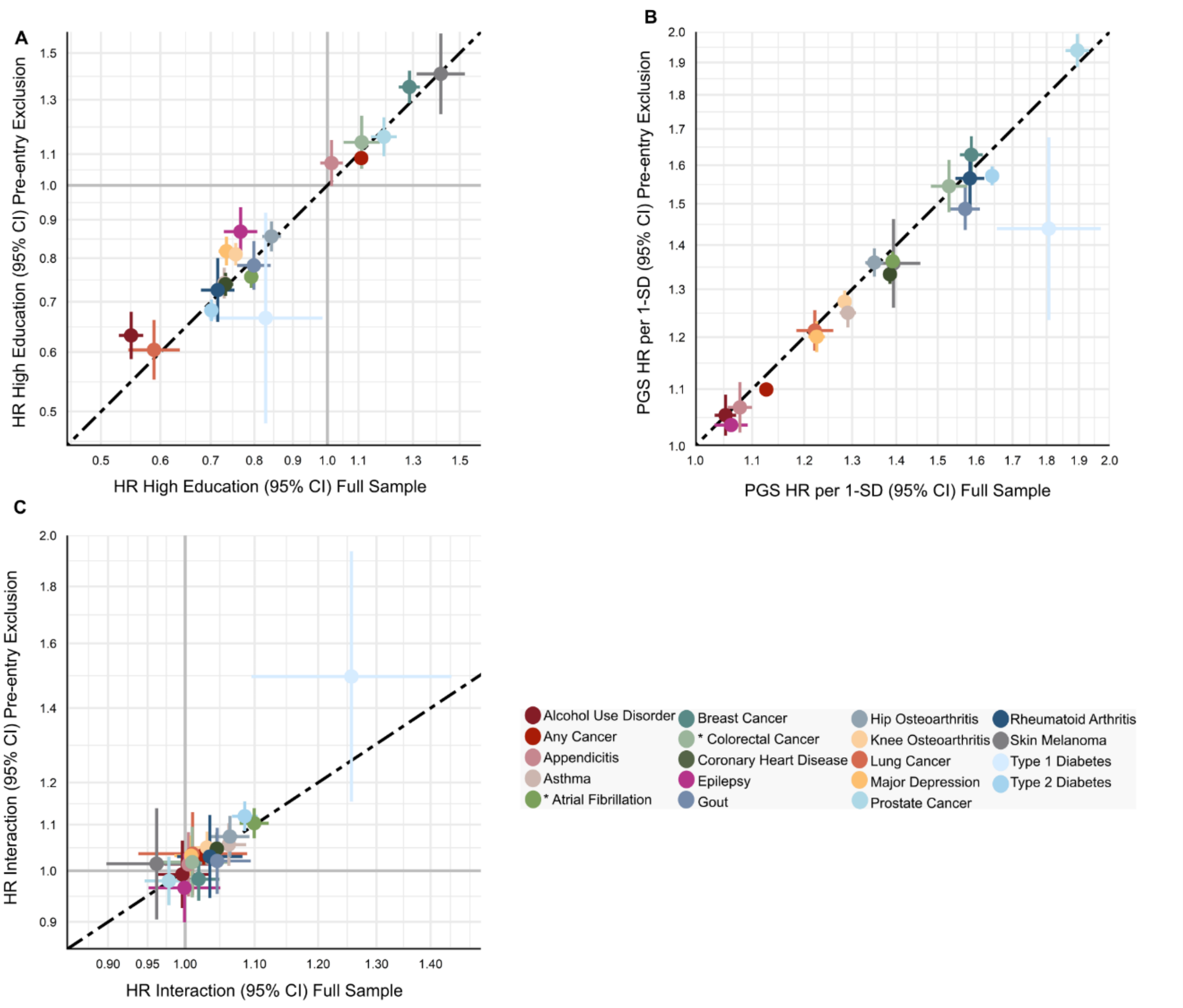

Supplementary Fig. 17. Comparison of the hazard ratios per standard deviation of the disease-specific polygenic scores, for individuals with high education relative to individuals with low education, and for the interaction between the disease-specific polygenic scores and educational attainment when including the full FinnGen sample versus after exclusion of mutual cases in FinnGen. **A.** Comparison of the hazard ratio for individuals with high education relative to low education in the full FinnGen sample (x-axis) versus after exclusion of mutual cases (y-axis). **B.** Comparison of the hazard ratios per standard deviation of the disease-specific polygenic scores on disease risk in the full FinnGen sample (x-axis) versus after exclusion of mutual cases (y-axis). **C.** Comparison of the hazard ratio of the interaction between the disease-specific polygenic score and educational attainment in the full FinnGen sample (x-axis) versus after exclusion of mutual cases (y-axis). The exact values are in **Supplementary Data 9** and **41-42**. Sample sizes by case-control status and educational attainment for each complex disease are in **Supplementary Data 4** and **40**.

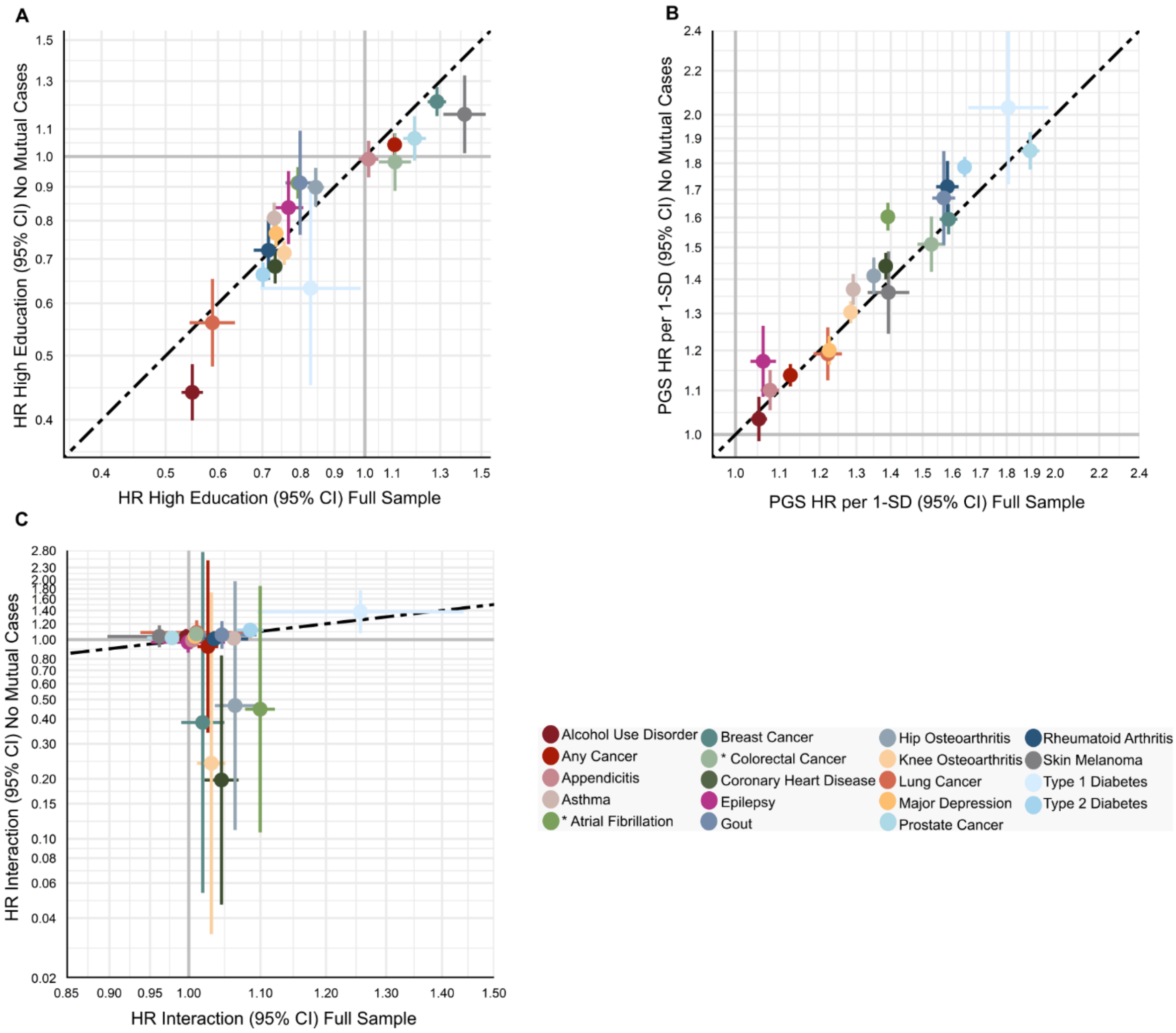

Supplementary Fig. 18. Comparison of the hazard ratios per standard deviation of the disease-specific polygenic scores, for individuals with high education relative to individuals with low education, and for the interaction between the disease-specific polygenic scores and educational attainment without versus with including a polygenic score for educational attainment and its interaction with the disease-specific polygenic scores. **A.** Comparison of the hazard ratio for individuals with high education relative to low education without (x-axis) versus with (y-axis) including a polygenic score for educational attainment and its interaction with the disease-specific polygenic scores in FinnGen. **B.** Comparison of the hazard ratios per standard deviation of the disease-specific polygenic scores on disease risk without (x-axis) versus with (y-axis) including a polygenic score for educational attainment and its interaction with the disease-specific polygenic scores in FinnGen. **C.** Comparison of the hazard ratio of the interaction between the disease-specific polygenic score and educational attainment without (x-axis) versus with (y-axis) including a polygenic score for educational attainment and its interaction with the disease-specific polygenic scores in FinnGen. The exact values are in **Supplementary Data 9** and **43-44**. Sample sizes by case-control status and educational attainment for each complex disease are in **Supplementary Data 4**.

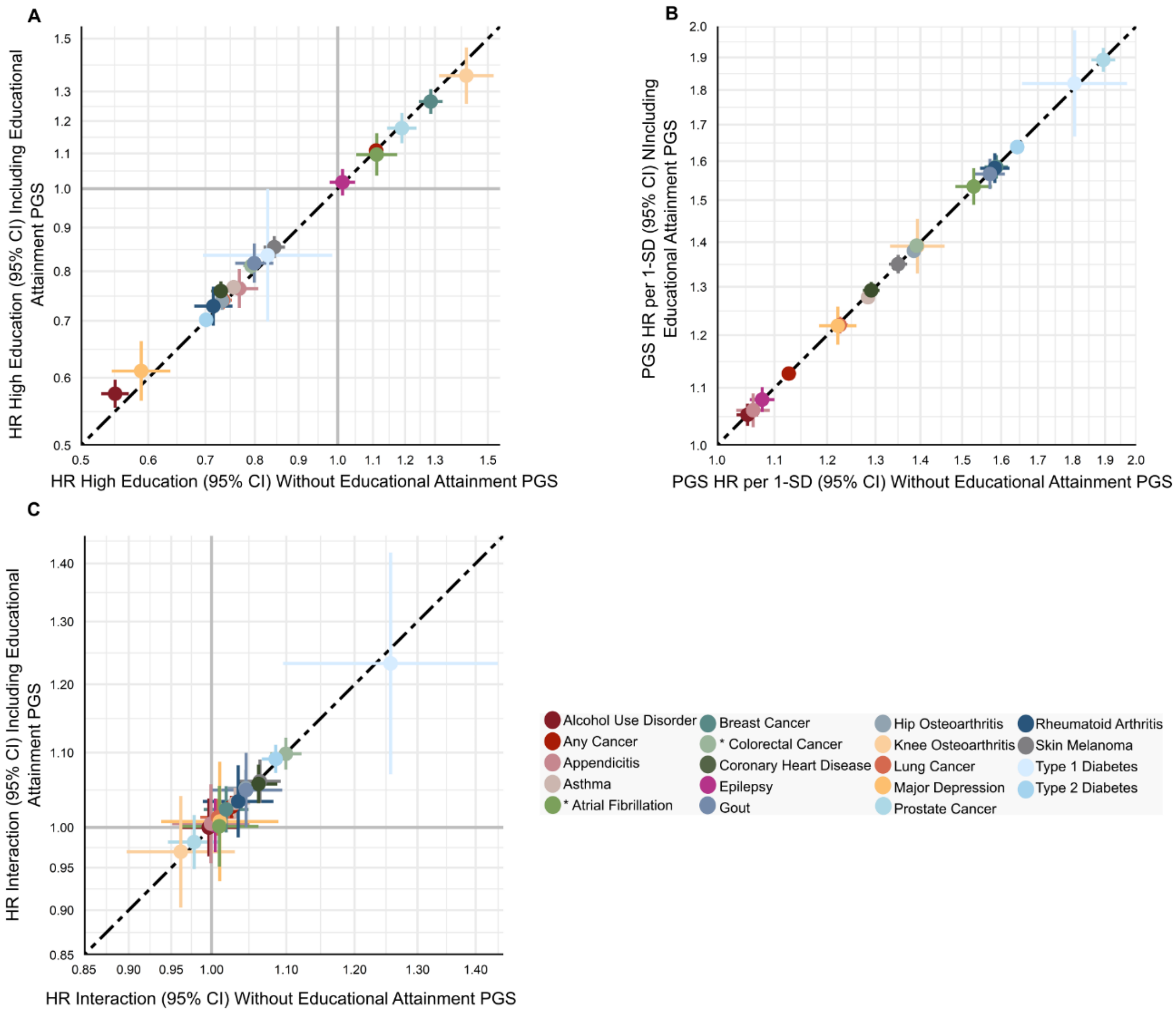
A
HR High Education (95% CI) Including Educational Attainment PGS
HR High Education (95% CI) Without Educational Attainment PGS
B
PGS HR per 1-SD (95% CI) NIncluding Educational Attainment PGS
PGS HR per 1-SD (95% CI) Without Educational Attainment PGS
C
HR Interaction (95% CI) Including Educational Attainment PGS
HR Interaction (95% CI) Without Educational Attainment PGS
Alcohol Use Disorder
Any Cancer
Appendicitis
Asthma
* Atrial Fibrillation
Breast Cancer
* Colorectal Cancer
Coronary Heart Disease
Epilepsy
Gout
Hip Osteoarthritis
Knee Osteoarthritis
Lung Cancer
Major Depression
Prostate Cancer
Rheumatoid Arthritis
Skin Melanoma
Type 1 Diabetes
Type 2 Diabetes

224

Supplementary Fig. 19. Comparison of the hazard ratios per standard deviation of the disease-specific polygenic scores, for individuals with high education relative to individuals with low education, and for the interaction between the disease-specific polygenic scores and educational attainment from Cox proportional hazard (Cox-PH) regression versus Fine-Gray (FG) competing risk models in FinnGen. **A.** Comparison of the hazard ratio for individuals with high education relative to low education in the Cox-PH models (x-axis) versus the FG models (y-axis). **B.** Comparison of the hazard ratios per standard deviation of the disease-specific polygenic scores on disease risk in the Cox-PH models (x-axis) versus the FG models (y-axis). **C.** Comparison of the hazard ratio of the interaction between the disease-specific polygenic score and educational attainment in the Cox-PH models (x-axis) versus the FG models (y-axis). The exact values are in **Supplementary Data 9** and **46-47**. Sample sizes by case-control status and educational attainment for each complex disease are in **Supplementary Data 4** and **45**.

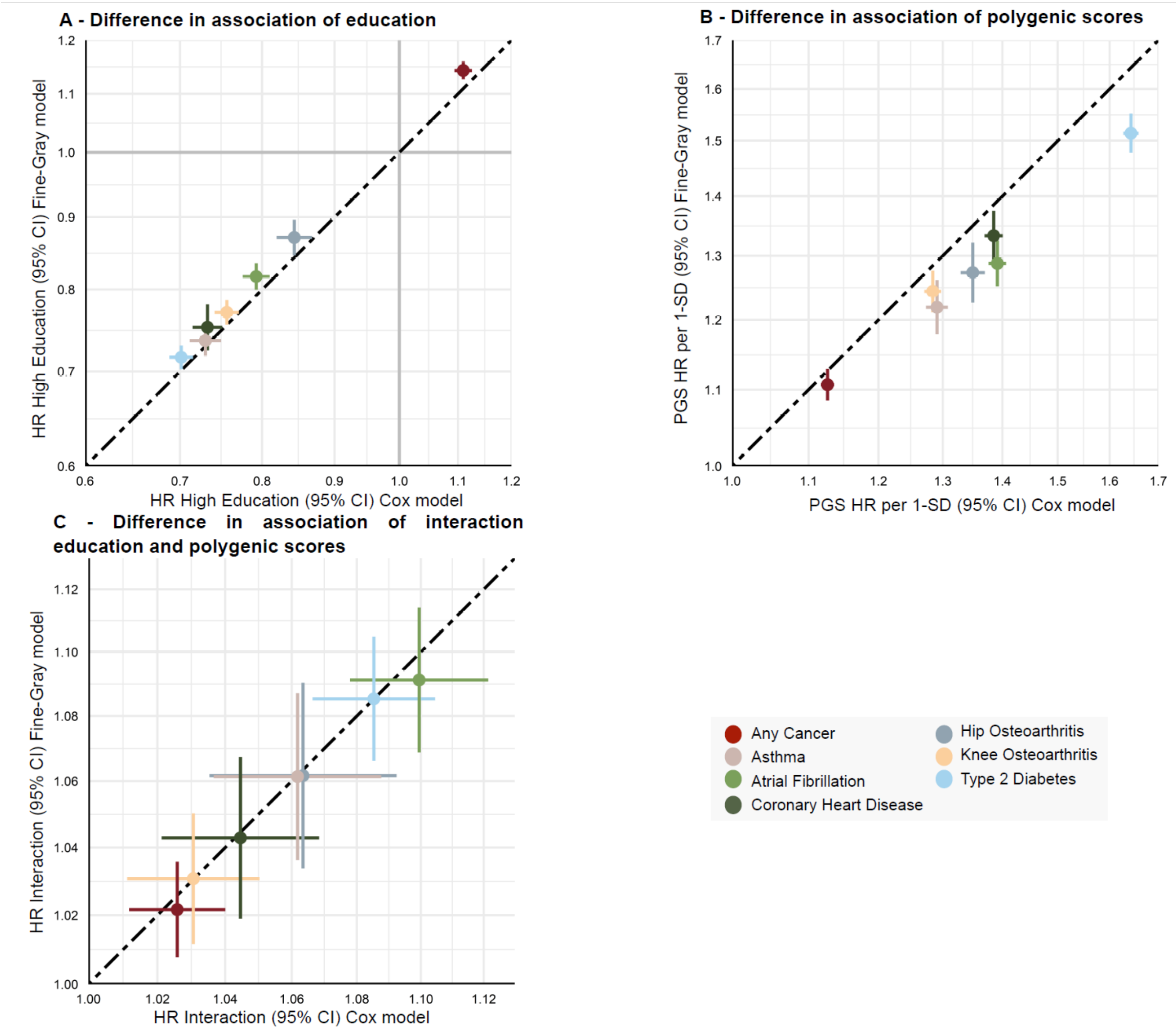

Supplementary Fig. 20. Ancestry-specific hazard ratios per standard deviation of the disease-specific polygenic scores (black) and for individuals with high education relative to individuals with low education (purple) on complex disease risk where the disease-specific polygenic scores and education were modelled separately in the UK Biobank. The exact values are in **Supplementary Data 5** and **49**. Sample sizes by case-control status and educational attainment for each complex disease in are in **Supplementary Data 4** and **48**.

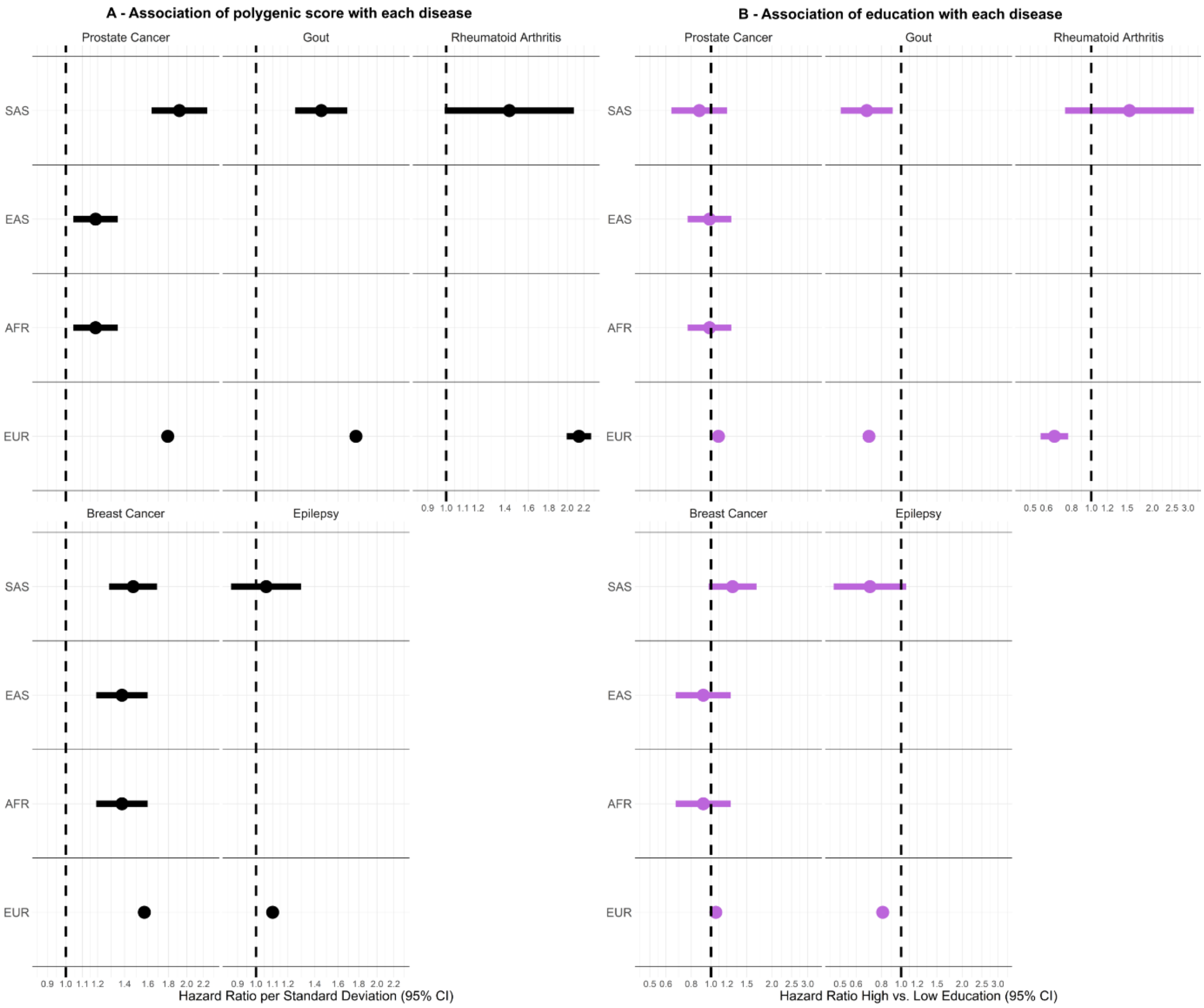

Supplement: Supplementary file 2 [file 43856_2026_1716_MOESM2_ESM.pdf]
