# Supplementary material for: Association and risk prediction of 19 complex diseases with polygenic scores and socioeconomic status
Source: Commun Med (Lond). 2026 Jun 11;6:503. doi: 10.1038/s43856-026-01716-1 (PMC13616955; doi:10.1038/s43856-026-01716-1)
Supplement: Supplementary file 3 [file 43856_2026_1716_MOESM3_ESM.pdf]

Legends Supplementary Data Files

Supplementary Data 1. Mapping of raw education data per biobank to 1997 International Standard Classification of Education.

Supplementary Data 2. Overview of sample and variant QC per genotyping batch in the data release 11 of the FinnGen Study.

Supplementary Data 3. Genome-wide association study summary statistics used to compute polygenic scores and sample overlap with each biobank study.

Supplementary Data 4. Descriptive statistics (prevalence, follow-up and age at onset) for each complex disease by biobank study.

Supplementary Data 5. Meta-analyzed and biobank study-specific hazard ratios per standard deviation of the association of high educational attainment and the disease-specific polygenic scores modelled separately.

Supplementary Data 6. Meta-analyzed and biobank study-specific hazard ratios per standard deviation of the association of high educational attainment and the disease-specific polygenic scores modelled jointly.

Supplementary Data 7. Differences in the association of educational attainment and the disease-specific polygenic scores when modelled separately or jointly as estimated with two-sided Wald test.

Supplementary Data 8. Meta-analyzed and biobank study-specific hazard ratios per standard deviation for the relative risk of the disease-specific polygenic scores by educational attainment.

Supplementary Data 9. Meta-analyzed and biobank study-specific hazard ratios per standard deviation for the association of high educational attainment, the disease-specific polygenic scores, and their interaction.

Supplementary Data 10. Descriptive statistics (prevalence, follow-up and age at onset) for each complex disease and polygenic score strata in FinnGen.

Supplementary Data 11. Hazard ratios per standard deviation for the association of each disease-specific polygenic score strata stratified by educational attainment in FinnGen.

Supplementary Data 12. Bootstrapped cumulative risk incidences for each complex disease by educational attainment in FinnGen.

Supplementary Data 13. Descriptive statistics (prevalence, follow-up and age at onset) for each complex disease in 80% of the FinnGen study.

Supplementary Data 14. Hazard ratio per standard deviation of the association of sex and birth year in 80% of the FinnGen study.

Supplementary Data 15. Hazard ratios per standard deviation of the association of high educational attainment and the disease-specific polygenic scores modelled separately in 80% of the FinnGen study.

Supplementary Data 16. Hazard ratio per standard deviation of the association of high educational attainment and the disease-specific polygenic scores modelled jointly in 80% of the FinnGen study.

Supplementary Data 17. Hazard ratio per standard of the association of high educational attainment, the disease-specific polygenic scores, and their interaction in 80% of the FinnGen study.

Supplementary Data 18. Descriptive statistics (prevalence, follow-up and age at onset) for each complex disease in 20% of the FinnGen study and for all 19 complex diseases in the UK Biobank.

Supplementary Data 19. Comparisons of the biobank-specific Areas Under the Curves (AUC) for a model with basic covariates (model 0b, including sex, birth year, and the first 10 genetic PCs) vs. a model including the disease-specific polygenic scores (model 1b); for a model with basic covariates (model 1a, including sex, birth year, and the first 5 genetic PCs) vs. a model including high educational attainment (model 1a); for a model with the disease-specific polygenic scores only (model 1b) vs. a model with high educational attainment and disease-specific polygenic scores modelled jointly (model 2); for a model with high educational attainment only (model 1a) vs. a model with high educational attainment and disease-specific polygenic scores modelled jointly (model 2); and for a model with high educational attainment and disease-specific polygenic scores modelled jointly (model 2) vs. a model with high educational attainment, disease-specific polygenic scores, and their interaction (model 4). DeLong's test was applied to test whether the AUCs were significantly different.

Supplementary Data 20. Comparisons of the biobank-specific Net Reclassification Indices (NRI) and Integrated Discrimination Indices (IDI) for a model with basic covariates (model 0b, including sex, birth year, and the first 10 genetic PCs) vs. a model including the disease-specific polygenic scores (model 1b); for a model with basic covariates (model 1a, including sex, birth year, and the first 5 genetic PCs) vs. a model including high educational attainment (model 1a); for a model with the disease-specific polygenic scores only (model 1b) vs. a model with high educational attainment and disease-specific polygenic scores modelled jointly (model 2); for a model with high educational attainment only (model 1a) vs. a model with high educational attainment and disease-specific polygenic scores modelled jointly (model 2); and for a model with high educational attainment and disease-specific polygenic scores modelled jointly

(model 2) vs. a model with high educational attainment, disease-specific polygenic scores, and their interaction (model 4).

Supplementary Data 21. Descriptive statistics (prevalence, follow-up and age at onset) for each complex trait in the occupation analyses by biobank study.

Supplementary Data 22. Meta-analyzed and biobank study-specific hazard ratios per standard deviation for the association of upper-level occupation and the disease-specific polygenic scores modelled separately.

Supplementary Data 23. Meta-analyzed and biobank study-specific hazard ratios per standard deviation for the association of upper-level occupation and the disease-specific polygenic scores modelled jointly.

Supplementary Data 24. Differences in the association of occupation and the disease-specific polygenic scores when modelled separately or jointly as estimated with two-sided Wald test.

Supplementary Data 25. Comparison of the meta-analysed hazard ratios per standard deviation for the association of high educational attainment or the disease-specific polygenic scores with the associations of upper-level occupation or the disease-specific polygenic scores.

Supplementary Data 26. Meta-analyzed and biobank study-specific hazard ratios per standard deviation for the relative risk of the disease-specific polygenic scores by occupation.

Supplementary Data 27. Meta-analyzed and biobank study-specific hazard ratios per standard deviation for the association of upper-level occupation, the disease-specific polygenic scores, and their interaction.

Supplementary Data 28. Comparison of the meta-analysed hazard ratios per standard deviation for the association of the disease-specific polygenic scores stratified by education with the associations the disease-specific polygenic scores stratified by occupation.

Supplementary Data 29. Descriptive statistics (prevalence, follow-up and age at onset) for each complex disease and polygenic score strata for the occupation analyses in the FinnGen study.

Supplementary Data 30. Hazard ratios per standard deviation for the association of each disease-specific polygenic score strata for the occupation analyses in the FinnGen study.

Supplementary Data 31. Bootstrapped cumulative risk incidences for each complex disease by occupation in the FinnGen study.

Supplementary Data 32. Descriptive statistics (prevalence, follow-up and age at onset) for each complex disease by biobank study when applying cohort-specific cutoffs for the dichotomization of educational attainment.

Supplementary Data 33. Meta-analyzed and biobank study-specific hazard ratios per standard deviation for the association of high educational attainment, the disease-specific polygenic scores, and their interaction when applying cohort-specific cutoffs for the dichotomization of educational attainment.

Supplementary Data 34. Comparison of the meta-analyzed hazard ratios per standard deviation for the association of high educational attainment, the disease-specific polygenic scores, and their interaction when applying the same cutoff versus cohort-specific cutoffs for the dichotomization of educational attainment.

Supplementary Data 35. Meta-analyzed and biobank study-specific hazard ratios per standard deviation for the association of high educational attainment, the disease-specific polygenic scores, and their interaction when educational attainment is not dichotomized.

Supplementary Data 36. Comparison of the meta-analyzed hazard ratios per standard deviation for the association of high educational attainment, the disease-specific polygenic scores, and their interaction when dichotomizing educational attainment versus when educational attainment is not dichotomized.

Supplementary Data 37. Descriptive statistics (prevalence, follow-up and age at onset) for each complex disease in FinnGen when excluding cases before cohort entry.

Supplementary Data 38. Hazard ratios per standard deviation for the association of high educational attainment, the disease-specific polygenic scores, and their interaction in FinnGen when excluding cases before cohort entry.

Supplementary Data 39. Comparison of the hazard ratios per standard deviation for the association of high educational attainment, the disease-specific polygenic scores, and their interaction in the full FinnGen sample versus when excluding cases before cohort entry.

Supplementary Data 40. Descriptive statistics (prevalence, follow-up and age at onset) for each complex disease in FinnGen when excluding mutual cases.

Supplementary Data 41. Hazard ratios per standard deviation for the association of high educational attainment, the disease-specific polygenic scores, and their interaction in FinnGen when excluding mutual cases.

Supplementary Data 42. Comparison of the hazard ratios per standard deviation for the association of high educational attainment, the disease-specific polygenic scores, and their interaction in the full FinnGen sample versus when excluding mutual cases.

Supplementary Data 43. Hazard ratios per standard deviation for the association of high educational attainment, the disease-specific polygenic scores, and their interaction in FinnGen when including an educational attainment polygenic score and its interaction with the disease-specific polygenic score in the model.

Supplementary Data 44. Comparison of the hazard ratios per standard deviation for the association of high educational attainment, the disease-specific polygenic scores, and their interaction in FinnGen when including an educational attainment polygenic score and its interaction with the disease-specific polygenic score in the model.

Supplementary Data 45. Descriptive statistics (prevalence, follow-up and age at onset) for each complex disease, including information on non-cause-specific deaths (deathsother) in the FinnGen study.

Supplementary Data 46. Subdistribution hazard ratios per standard deviation for the association of high educational attainment, the disease-specific polygenic scores, and their interaction in the FinnGen study.

Supplementary Data 47. Comparison of the hazard ratios per standard deviation (Cox proportional hazard models) and subdistribution hazard ratios per standard deviation (Fine-Gray models) for the association of high educational attainment, the disease-specific polygenic scores, and their interaction in the FinnGen study.

Supplementary Data 48. Descriptive statistics (prevalence, follow-up and age at onset) for each complex disease in each non-European ancestry in the UK Biobank.

Supplementary Data 49. Hazard ratios per standard deviation for the association of high educational attainment and the disease-specific polygenic scores modelled separately in each non-European ancestry in the UK Biobank.
